# New records of semi-aquatic bugs (Hemiptera, Heteroptera, Gerromorpha) from the South Region of Brazil

**DOI:** 10.3897/BDJ.14.e163787

**Published:** 2026-02-05

**Authors:** Carla FB Floriano, Nathália de Oliveira Paiva, Isabelle da Rocha Silva Cordeiro, Cleilton Lima Franco, Juliana Mourão dos Santos Rodrigues, Felipe Ferraz Figueiredo Moreira

**Affiliations:** 1 Fundação Oswaldo Cruz, Instituto Oswaldo Cruz, Laboratório de Entomologia, Rio de Janeiro, Brazil Fundação Oswaldo Cruz, Instituto Oswaldo Cruz, Laboratório de Entomologia Rio de Janeiro Brazil; 2 Universidade Federal Rural do Rio de Janeiro, Programa de Pós-graduação em Biologia Animal, Seropédica, Brazil Universidade Federal Rural do Rio de Janeiro, Programa de Pós-graduação em Biologia Animal Seropédica Brazil

**Keywords:** aquatic insects, faunistics, Neotropical Region, true bugs

## Abstract

**Background:**

Semi-aquatic bug (Hemiptera, Heteroptera, Gerromorpha) constitute a monophyletic group of predatory insects that play crucial roles in limnic environments. The Oriental and Neotropical regions harbor the richest faunas, but many species from these areas remain undescribed, and large regions are still poorly explored. In Brazil, semi-aquatic bugs have been more thoroughly sampled in the North and Southeast regions, while other regions have been neglected. The Southern Region of the country comprises the states of Paraná, Santa Catarina, and Rio Grande do Sul, from which 20, 44, and 28 species of Gerromorpha have been recorded, respectively. In total, 61 species are known from the region, which is likely far from being an accurate representation of the local fauna.

**New information:**

Here, we present new state records of 24 species of Gerromorpha from the State of Paraná, nine from Santa Catarina and six from Rio Grande do Sul. Furthermore, of the 45 species that we studied, 36 are recorded for the first time from at least one of the three States. Our results also include one first record from Brazil and 14 from the South Region. Additionally, we identified and provide illustrations of a male of *Mesovelia amoena* Uhler, 1894, which is extremely rare in the Neotropics. Finally, the new data presented here increases the number of semi-aquatic bug species known from the South Region from 60 to 75.

## Introduction

Aquatic and semi-aquatic bugs (Hemiptera, Heteroptera, Nepomorpha & Gerromorpha) constitute monophyletic groups of mostly predatory insects that play crucial roles in limnic environments (Barbosa and Rodrigues 2015, Moreira 2015). The Oriental and Neotropical Regions harbour the richest faunas, but many species from these areas remain undescribed and large regions are still poorly explored (Polhemus and Polhemus 2007, Polhemus and Polhemus 2008). Although there is no updated checklist or catalogue of the Neotropical fauna, Colombia and Brazil are likely the two countries in the region with the highest numbers of species, as has been demonstrated for some of the included taxa (Molano et al. 2005, Moreira et al. 2011a, Padilla-Gil and Moreira 2013).

The latest article compiling information on the aquatic and semi-aquatic bugs recorded from Brazil listed 479 species of Gerromorpha and Nepomorpha from the country (Moreira et al. 2011a). Out of them, 145 had been recorded from the Brazilian South Region, which comprises the States of Paraná (50 species), Santa Catarina (89) and Rio Grande do Sul (61). The higher diversity found in the State of Santa Catarina State can probably be explained by the huge collecting efforts of the naturalist Fritz Plaumann in the early 20^th^ century, concentrated in the surroundings of the locality of Nova Teutônia, now part of the Municipality of Seara (Moreira et al. 2011a).

Although stagnant for decades, collecting effort targeting Gerromorpha in southern Brazil has increased since 2011 and the Taxonomic Catalogue of the Brazilian Fauna now indicates the presence of 61 species in the region. From this total, 20 species have been recorded from the State of Paraná, 44 from Santa Catarina and 28 from Rio Grande do Sul (Moreira 2025a, Moreira 2025b, Moreira 2025c, Moreira 2025d, Moreira 2025e). However, there is only one recent study focusing on the semi-aquatic bugs from the region (Rodrigues et al. 2021), whereas all other records are sparse in literature (e.g. Moreira and Campos (2012), Rodrigues et al. (2012), Floriano et al. (2017), Rodrigues and Moreira (2016), Moreira et al. (2020), Rodrigues et al. (2021), Armisén et al. (2022), Heiss et al. (2022)). Here, we present new records of Gerromorpha from southern Brazil, based mostly on recently collected material, as well as an updated list of the species known from the region.

## Materials and methods

**Figure 1.**
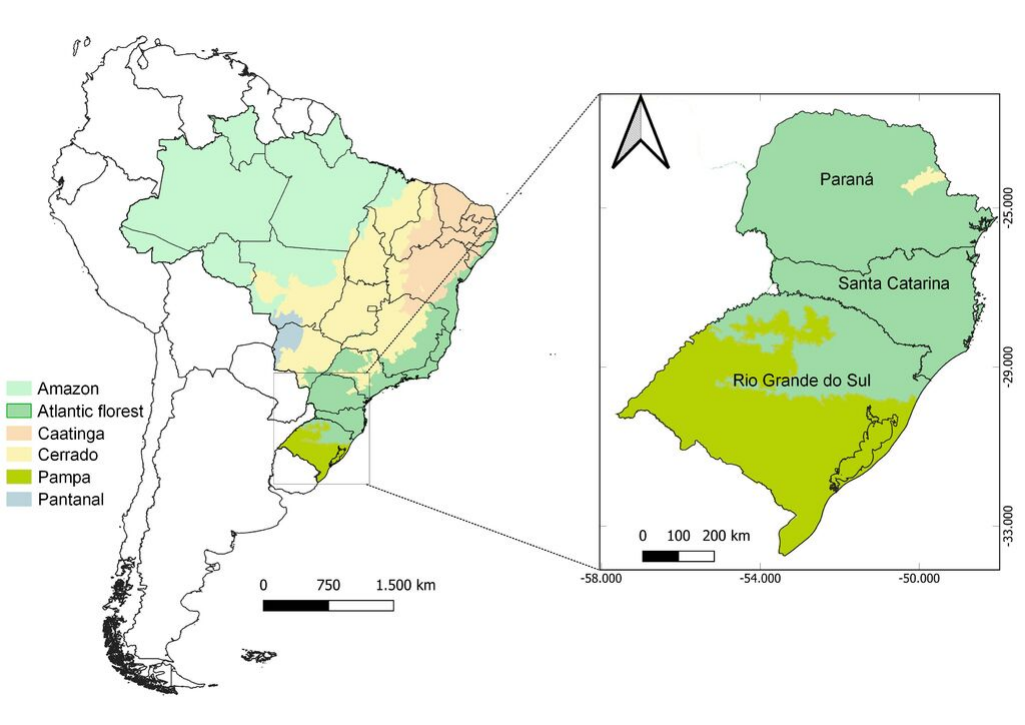
Maps showing the Brazilian biomes and the South Region of Brazil (study area).

For this study, we performed a series of field trips to the South Region (Fig. 1), from 2016 to 2024. We collected in 21 municipalities of the States of Paraná (PR) (Balsa Nova, Cianorte, Faxinal, Marialva, Morretes, Quatro Barras, Ponta Grossa, Tibagi, Tuneiras do Oeste), Santa Catarina (SC) (Águas Mornas, Blumenau, Corupá, Indaial, Mafra, Praia Grande, Rancho Queimado, Rio Negrinho, Santo Amaro da Imperatriz, São Bonifácio, Urubici) and Rio Grande do Sul (RS) (Cambará do Sul). Amongst the collecting stations, we included four federal conservation areas (States between parentheses): Parque Nacional da Serra de Itajaí (SC), Parque Nacional de Aparados da Serra (SC, RS), Parque Nacional da Serra Geral (SC, RS) and Reserva Biológica das Perobas (PR). This recently collected material is preserved in ethanol and deposited in the Coleção Entomológica do Instituto Oswaldo Cruz, Fundação Oswaldo Cruz, Rio de Janeiro, Brazil (CEIOC).

We examined additional material deposited in the following institutions: Coleção Entomológica Prof. José Alfredo Pinheiro Dutra, Laboratório de Entomologia, Departamento de Zoologia, Universidade Federal do Rio de Janeiro, Rio de Janeiro, Brazil (DZRJ); Museu de Zoologia da Universidade de São Paulo, São Paulo, Brazil (MZUSP); and National Museum of Natural History, Smithsonian Institution, Washington D.C., United States (NMNH).

We obtained photographs of the specimens using a Leica M205 C stereomicroscope coupled with a Leica DMC 2900 digital camera and combined the images into single composite figures using the Leica Application Suite V.4.7 extended depth of focus module. We made enhancements to the images using Adobe Photoshop CS5. We produced maps using Qgis 2.6.1. The distribution presented for each species is according to Moreira (2025a), Moreira (2025b), Moreira (2025c), Moreira (2025d), Moreira (2025e) and abbreviations of Brazilian states are according to the official standard (Instituto Brasileiro de Geografia e Estatística 2025). We identified specimens using keys provided by Nieser and Melo (1997), Moreira (2015), Rodrigues and Moreira (2016), Moreira et al. (2020), Magalhães et al. (2021) and Rodrigues et al. (2021) and, whenever possible, by comparison with type series specimens.

## Checklists

### Gerromorpha from the South Region of Brazil

#### Gerridae

Leach, 1815

7561FDB3-EE1D-5C52-A582-318DBC1951F4

#### Charmatometrinae

Matsuda, 1960

15A1FAF5-1304-5947-9375-E37B1B7FCE8F

#### Brachymetra

Mayr, 1865

A8F8B852-7268-5CC6-9572-EDE428DEFC84

#### Brachymetra albinervus

(Amyot & Serville, 1843)

56B66A03-3E3C-5B52-AE58-CCD408C415AF

#### Brachymetra albinervus albinervus

(Amyot & Serville, 1843)

29E9E352-A425-5A53-BB47-78A04A884C5E

##### Materials

- **Type status:** Other material. **Occurrence:** recordedBy: T. Roeli & T. Polizei; individualCount: 1; sex: male; lifeStage: adult; occurrenceID: 0FE88511-3664-56FC-963E-57FBFC28BFD6; **Taxon:** genus: Brachymetra; specificEpithet: albinervus; infraspecificEpithet: albinervus; taxonRank: Subspecies; scientificNameAuthorship: (Amyot & Serville, 1843); **Location:** stateProvince: Santa Catarina; municipality: Rio Negrinho; locality: Rio dos Bugres; decimalLatitude: -26.2772; decimalLongitude: -49.5344; **Identification:** identificationID: Brachymetra albinervus albinervus; identifiedBy: I. R. S. Cordeiro; **Event:** samplingProtocol: "D" Net; year: 2020; month: III; **Record Level:** institutionCode: CEIOC; basisOfRecord: PreservedSpecimen
- **Type status:** Other material. **Occurrence:** recordedBy: C. F. B. Floriano; individualCount: 4; sex: male; lifeStage: adult; occurrenceID: A2BDC6AF-68B1-5B15-9B1D-ED79FFD12248; **Taxon:** genus: Brachymetra; specificEpithet: albinervus; infraspecificEpithet: albinervus; taxonRank: Subspecies; scientificNameAuthorship: (Amyot & Serville, 1843); **Location:** stateProvince: Paraná; municipality: Marialva; locality: Ribeirão Sarandi, Cachoeira Marialva; decimalLatitude: -23.4423; decimalLongitude: -51.7695; **Identification:** identificationID: Brachymetra albinervus albinervus; identifiedBy: I. R. S. Cordeiro; **Event:** samplingProtocol: "D" Net; year: 2024; month: III; **Record Level:** institutionCode: CEIOC; basisOfRecord: PreservedSpecimen

##### Distribution

Guatelama, Honduras, Guadeloupe, Dominica, Martinique, St. Lucia, Costa Rica, Panama, St. Vincent & the Grenadines, Grenada, Trinidad & Tobago, Colombia, Venezuela, Suriname, French Guiana, Brazil, Ecuador, Peru and Paraguay.

##### Notes

**Figure 2.**
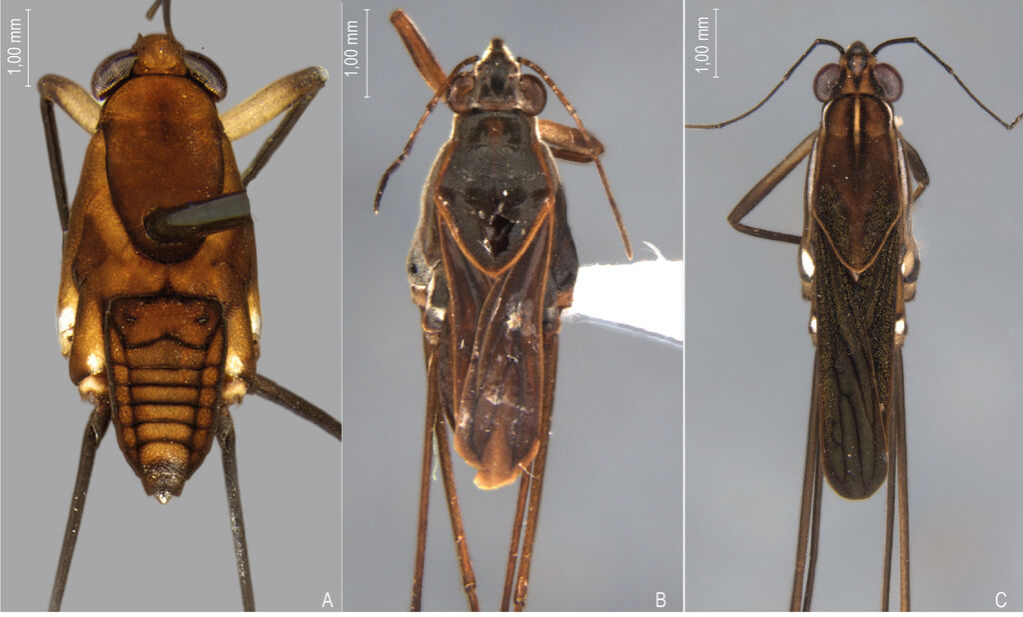
Gerridae from the South Region of Brazil, male, habitus, dorsal view. **A** *Brachymetra albinervus alvinervus*; **B** *Neogerris lubricus*; **C** *Tachygerris adamsoni*.

First records from Paraná and Santa Catarina. Previously reported from Rio Grande do Sul by Rodrigues et al. (2021), (Fig. 2A, Fig. 4A).

#### Gerrinae

Leach, 1815

C2428BDD-5DBF-5A8D-9E4C-36EF31239258

#### Limnogonus

Stål, 1868

903892C4-31EE-5ED8-9FC6-6222D03E3EE6

#### Limnogonus aduncus

Drake & Harris, 1933

8EC3FD75-737F-5346-BC2D-64A1A8D9F6B1

#### Limnogonus aduncus aduncus

Drake & Harris, 1933

CBE0BC6A-B483-5DDB-BC05-1B52AD3616F5

##### Materials

- **Type status:** Other material. **Occurrence:** recordedBy: C. F. B. Floriano & T. Malacarne; individualCount: 1; sex: male; lifeStage: adult; occurrenceID: 94AD254F-9BA0-5390-AB80-D403197FE937; **Taxon:** genus: Limnogonus; specificEpithet: aduncus; infraspecificEpithet: aduncus; taxonRank: Subspecies; scientificNameAuthorship: Drake & Harris, 1933; **Location:** stateProvince: Santa Catarina; municipality: Blumenau; locality: Parque Nacional da Serra do Itajaí, Parque Natural Municipal Nascentes do Garcia, Lagoa Negra; decimalLatitude: -27.0532; decimalLongitude: -49.0878; **Identification:** identificationID: Limnogonus aduncus aduncus; identifiedBy: C. F. B. Floriano; **Event:** samplingProtocol: "D" Net; year: 2017; month: IV; **Record Level:** institutionCode: CEIOC; basisOfRecord: PreservedSpecimen
- **Type status:** Other material. **Occurrence:** recordedBy: C. F. B. Floriano; individualCount: 3; sex: male; lifeStage: adult; occurrenceID: E900FC7C-6866-5B79-BA8A-3ECDCB2CEFE9; **Taxon:** genus: Limnogonus; specificEpithet: aduncus; infraspecificEpithet: aduncus; taxonRank: Subspecies; scientificNameAuthorship: Drake & Harris, 1933; **Location:** stateProvince: Paraná; municipality: Paranaguá; locality: Ilha do Mel, pousada Ephira; decimalLatitude: -25.5656; decimalLongitude: -48.3153; **Identification:** identificationID: Limnogonus aduncus aduncus; identifiedBy: C. F. B. Floriano; **Event:** samplingProtocol: "D" Net; year: 2016; month: X; **Record Level:** institutionCode: CEIOC; basisOfRecord: PreservedSpecimen
- **Type status:** Other material. **Occurrence:** recordedBy: C. F. B. Floriano; individualCount: 2; sex: female; lifeStage: adult; occurrenceID: 80458F0D-B36C-5FF7-B6A2-8DBEAE5B078C; **Taxon:** genus: Limnogonus; specificEpithet: aduncus; infraspecificEpithet: aduncus; taxonRank: Subspecies; scientificNameAuthorship: Drake & Harris, 1933; **Location:** stateProvince: Paraná; municipality: Paranaguá; locality: Ilha do Mel, pousada Ephira; decimalLatitude: -25.5656; decimalLongitude: -48.3153; **Identification:** identificationID: Limnogonus aduncus aduncus; identifiedBy: C. F. B. Floriano; **Event:** samplingProtocol: "D" Net; year: 2016; month: X; **Record Level:** institutionCode: CEIOC; basisOfRecord: PreservedSpecimen

##### Distribution

Costa Rica, Panama, Trinidad & Tobago, Colombia, Venezuela, Guyana, Suriname, Brazil, Ecuador, Peru, Bolivia, Paraguay and Argentina.

##### Notes

Previously reported from Paraná and Santa Catarina by Moreira and Campos (2012)

#### Neogerris

Matsumura, 1913

DCC460A9-B320-56FF-ADD2-22EE13A07981

#### Neogerris lubricus

(White, 1879)

93019D54-66AE-546C-A724-76231A9E196B

##### Materials

- **Type status:** Other material. **Occurrence:** recordedBy: C. F. B. Floriano & T. Malacarne; individualCount: 1; sex: male; lifeStage: adult; occurrenceID: 465B6736-3273-5D9A-90D4-8021FC6DE546; **Taxon:** genus: Neogerris; specificEpithet: lubricus; taxonRank: species; scientificNameAuthorship: (White, 1879); **Location:** stateProvince: Santa Catarina; municipality: Blumenau; locality: Parque Nacional da Serra do Itajaí, Parque Natural Municipal Nascentes do Garcia, Lagoa da Sede; decimalLatitude: -27.0531; decimalLongitude: -49.0868; **Identification:** identificationID: Neogerris lubricus; identifiedBy: C. F. B. Floriano; **Event:** samplingProtocol: "D" Net; year: 2017; month: IV; **Record Level:** institutionCode: CEIOC; basisOfRecord: PreservedSpecimen

##### Distribution

Costa Rica, Panama, Trinidad & Tobago, Colombia, Venezuela, Guyana, Suriname, French Guiana, Brazil, Ecuador, Peru, Bolivia, Paraguay and Argentina.

##### Notes

First record from Santa Catarina. Previously recorded from Rio Grande do Sul by Rodrigues et al. (2021), (Fig. 2B, Fig. 4A).

#### Tachygerris

Drake, 1957

16157683-7F56-5869-A53A-4F9CA89721FA

#### Tachygerris adamsoni

(Drake, 1942)

8ADD1B11-5A6F-5825-94C8-94E49F3E6C3F

##### Materials

- **Type status:** Other material. **Occurrence:** recordedBy: C. F. B. Floriano; individualCount: 1; sex: male; lifeStage: adult; occurrenceID: E3368CD7-BEF3-57D3-8D3F-D80459279E60; **Taxon:** genus: Tachygerris; specificEpithet: adamsoni; taxonRank: species; scientificNameAuthorship: (Drake, 1942); **Location:** stateProvince: Paraná; municipality: Cianorte; locality: Reserva Biológica das Perobas, Córrego Adelaide; verbatimElevation: 457 m; decimalLatitude: -23.8472; decimalLongitude: -52.7389; **Identification:** identificationID: Tachygerris adamsoni; identifiedBy: C. F. B. Floriano; **Event:** samplingProtocol: "D" Net; year: 2024; month: IV; **Record Level:** institutionCode: CEIOC; basisOfRecord: PreservedSpecimen

##### Distribution

Trinidad & Tobago, Colombia, Venezuela, Suriname, French Guiana, Brazil, Peru, Bolivia, Paraguay.

##### Notes

First record from Paraná and the South Region of Brazil (Fig. 2C, Fig. 4A).

#### Microveliinae

China & Usinger, 1949

1DF6ECC2-AAA9-514F-A426-6D55F0E3AB9F

#### Microvelia

Westwood, 1834

D829FDE3-9EB5-5C77-982C-3E0A1C3062BE

#### Microvelia arca

Drake, 1958

59748E23-B1E8-54D2-8680-50D3611AD719

##### Materials

- **Type status:** Holotype. **Occurrence:** recordedBy: F. Plaumann; individualCount: 1; sex: macho; lifeStage: adult; occurrenceID: 33399C42-4E3B-5C41-BD47-76D1654A45C0; **Taxon:** genus: Microvelia; specificEpithet: arca; taxonRank: species; scientificNameAuthorship: Drake, 1958; **Location:** stateProvince: Santa Catarina; municipality: Chapecó; locality: Linha facão; decimalLatitude: -27.1; decimalLongitude: -52.6; **Identification:** identificationID: Microvelia arca; identifiedBy: C. Drake; **Event:** samplingProtocol: "D" Net; year: 1957; month: V; **Record Level:** institutionCode: NMNH; basisOfRecord: PreservedSpecimen
- **Type status:** Other material. **Occurrence:** recordedBy: F. Plaumann; individualCount: 3; sex: male; lifeStage: adult; occurrenceID: 9E6B7E4F-C617-521A-99CB-A35CEB4BA6DC; **Taxon:** genus: Microvelia; specificEpithet: arca; taxonRank: species; scientificNameAuthorship: Drake, 1958; **Location:** stateProvince: Santa Catarina; municipality: Chapecó; locality: Linha facão; decimalLatitude: -27.1; decimalLongitude: -52.6; **Identification:** identificationID: Microvelia arca; identifiedBy: C. F. B. Floriano; **Event:** samplingProtocol: "D" Net; year: 1956; **Record Level:** institutionCode: MZUSP; basisOfRecord: PreservedSpecimen
- **Type status:** Other material. **Occurrence:** recordedBy: F. Plaumann; individualCount: 3; sex: female; lifeStage: adult; occurrenceID: 3E697C63-2652-5A2E-9EB8-DD9DA150B060; **Taxon:** genus: Microvelia; specificEpithet: arca; taxonRank: species; scientificNameAuthorship: Drake, 1958; **Location:** stateProvince: Santa Catarina; municipality: Chapecó; locality: Linha facão; decimalLatitude: -27.1; decimalLongitude: -52.6; **Identification:** identificationID: Microvelia arca; identifiedBy: C. F. B. Floriano; **Event:** samplingProtocol: "D" Net; year: 1956; **Record Level:** institutionCode: MZUSP; basisOfRecord: PreservedSpecimen

##### Distribution

Brazil.

##### Notes

Originally described from Santa Catarina (Drake 1958a).

#### Microvelia braziliensis

McKinstry, 1937

C94EEE10-2BF0-5811-9F2E-4810B63955AB

##### Materials

- **Type status:** Other material. **Occurrence:** recordedBy: C. F. B. Floriano; individualCount: 3; sex: male; lifeStage: adult; occurrenceID: 516E493F-A831-5AD7-941D-BEB539A0E3C1; **Taxon:** genus: Microvelia; specificEpithet: braziliensis; taxonRank: species; scientificNameAuthorship: McKinstry, 1937; **Location:** stateProvince: Paraná; municipality: Marialva; locality: Ribeirão Sarandi, Cachoeira Marialva; decimalLatitude: -23.4423; decimalLongitude: -51.7695; **Identification:** identificationID: Microvelia braziliensis; identifiedBy: C. F. B. Floriano; **Event:** samplingProtocol: "D" Net; year: 2024; **Record Level:** institutionCode: CEIOC; basisOfRecord: PreservedSpecimen
- **Type status:** Other material. **Occurrence:** recordedBy: C. F. B. Floriano; individualCount: 5; sex: female; lifeStage: adult; occurrenceID: 68975BEB-0405-5295-8A85-8B6C4ED3910A; **Taxon:** genus: Microvelia; specificEpithet: braziliensis; taxonRank: species; scientificNameAuthorship: McKinstry, 1937; **Location:** stateProvince: Paraná; municipality: Marialva; locality: Ribeirão Sarandi, Cachoeira Marialva; decimalLatitude: -23.4423; decimalLongitude: -51.7695; **Identification:** identificationID: Microvelia braziliensis; identifiedBy: C. F. B. Floriano; **Event:** samplingProtocol: "D" Net; year: 2024; **Record Level:** institutionCode: CEIOC; basisOfRecord: PreservedSpecimen
- **Type status:** Other material. **Occurrence:** recordedBy: C. F. B. Floriano; individualCount: 3; sex: male; lifeStage: adult; occurrenceID: 1FF508B8-4AB1-595F-89C7-D8147471E74A; **Taxon:** genus: Microvelia; specificEpithet: braziliensis; taxonRank: species; scientificNameAuthorship: McKinstry, 1937; **Location:** stateProvince: Paraná; municipality: Tuneiras do Oeste; locality: Reserva Biológica das Perobas, brejo na trilha; decimalLatitude: -23.8883; decimalLongitude: -52.8086; **Identification:** identificationID: Microvelia braziliensis; identifiedBy: C. F. B. Floriano; **Event:** samplingProtocol: "D" Net; year: 2024; month: IV; **Record Level:** institutionCode: CEIOC; basisOfRecord: PreservedSpecimen
- **Type status:** Other material. **Occurrence:** recordedBy: C. F. B. Floriano; individualCount: 2; sex: female; lifeStage: adult; occurrenceID: 7D9A11CC-8A84-5927-982B-CF686E81BBA5; **Taxon:** genus: Microvelia; specificEpithet: braziliensis; taxonRank: species; scientificNameAuthorship: McKinstry, 1937; **Location:** stateProvince: Paraná; municipality: Tuneiras do Oeste; locality: Reserva Biológica das Perobas, brejo na trilha; decimalLatitude: -23.8883; decimalLongitude: -52.8086; **Identification:** identificationID: Microvelia braziliensis; identifiedBy: C. F. B. Floriano; **Event:** samplingProtocol: "D" Net; year: 2024; month: IV; **Record Level:** institutionCode: CEIOC; basisOfRecord: PreservedSpecimen
- **Type status:** Other material. **Occurrence:** recordedBy: C. F. B. Floriano; individualCount: 1; sex: male; lifeStage: adult; occurrenceID: D4DBCA8C-26AA-5619-AF50-89A9321BDBC7; **Taxon:** genus: Microvelia; specificEpithet: braziliensis; taxonRank: species; scientificNameAuthorship: McKinstry, 1937; **Location:** stateProvince: Paraná; municipality: Tuneiras do Oeste; locality: Reserva Biológica das Perobas, Rio Saquarema; decimalLatitude: -23.8725; decimalLongitude: -52.7864; **Identification:** identificationID: Microvelia braziliensis; identifiedBy: C. F. B. Floriano; **Event:** samplingProtocol: "D" Net; year: 2024; month: IV; **Record Level:** institutionCode: CEIOC; basisOfRecord: PreservedSpecimen
- **Type status:** Other material. **Occurrence:** recordedBy: C. F. B. Floriano; individualCount: 1; sex: female; lifeStage: adult; occurrenceID: E4E2650B-790A-5DB0-8BE4-5F661C41175C; **Taxon:** genus: Microvelia; specificEpithet: braziliensis; taxonRank: species; scientificNameAuthorship: McKinstry, 1937; **Location:** stateProvince: Paraná; municipality: Tuneiras do Oeste; locality: Reserva Biológica das Perobas, Rio Saquarema; decimalLatitude: -23.8725; decimalLongitude: -52.7864; **Identification:** identificationID: Microvelia braziliensis; identifiedBy: C. F. B. Floriano; **Event:** samplingProtocol: "D" Net; year: 2024; month: IV; **Record Level:** institutionCode: CEIOC; basisOfRecord: PreservedSpecimen
- **Type status:** Other material. **Occurrence:** recordedBy: C. F. B. Floriano; individualCount: 4; sex: male; lifeStage: adult; occurrenceID: 012F5413-393E-52F4-8C0E-3DD7866D7550; **Taxon:** genus: Microvelia; specificEpithet: braziliensis; taxonRank: species; scientificNameAuthorship: McKinstry, 1937; **Location:** stateProvince: Paraná; municipality: Cianorte; locality: Reserva Biológica das Perobas, Córrego Adelaide; decimalLatitude: -23.8472; decimalLongitude: -52.7389; **Identification:** identificationID: Microvelia braziliensis; identifiedBy: C. F. B. Floriano; **Event:** samplingProtocol: "D" Net; year: 2024; month: IV; **Record Level:** institutionCode: CEIOC; basisOfRecord: PreservedSpecimen
- **Type status:** Other material. **Occurrence:** recordedBy: C. F. B. Floriano; individualCount: 3; sex: female; lifeStage: adult; occurrenceID: C9E1EEF3-516F-5D35-871E-0E65EFD1CA47; **Taxon:** genus: Microvelia; specificEpithet: braziliensis; taxonRank: species; scientificNameAuthorship: McKinstry, 1937; **Location:** stateProvince: Paraná; municipality: Cianorte; locality: Reserva Biológica das Perobas, Córrego Adelaide; decimalLatitude: -23.8472; decimalLongitude: -52.7389; **Identification:** identificationID: Microvelia braziliensis; identifiedBy: C. F. B. Floriano; **Event:** samplingProtocol: "D" Net; year: 2024; month: IV; **Record Level:** institutionCode: CEIOC; basisOfRecord: PreservedSpecimen

##### Distribution

Brazil, Ecuador, Peru, Bolivia, Paraguay and Argentina.

##### Notes

**Figure 3.**
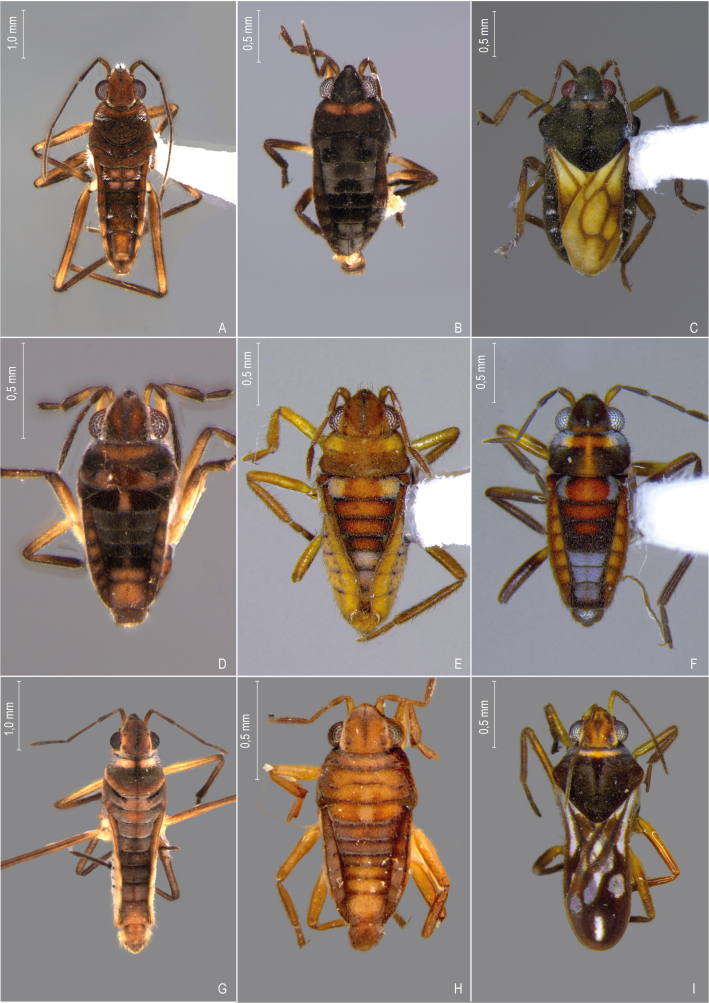
Gerridae from the South Region of Brazil, habitus, dorsal view. **A**–**B, D, F**–**I**: Male. **C, E** Female. **A** *Microvelia braziliensis*; **B** *Microvelia hamadae*; **C** *Microvelia hambletoni*; **D** *Microvelia hinei*; **E** *Microvelia ioana*; **F** *Microvelia limaiana*; **G** *Microvelia longipes*; **H** *Microvelia lujanana*; **I** *Microvelia mimula*.

**Figure 4.**
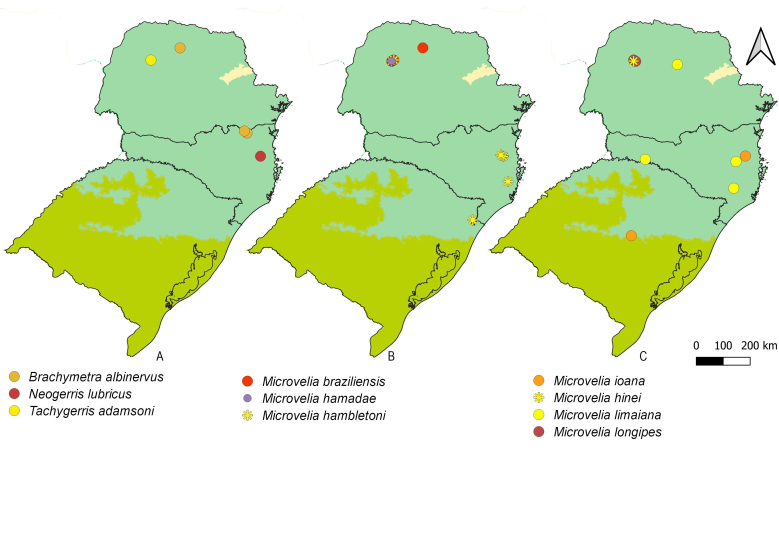
Maps showing new records of Gerridae (*Brachymetra*, *Microvelia*, *Neogerris* and *Tachygerris*) from the South Region of Brazil.

First records from Paraná (Fig. 3A, Fig. 4B). Previously reported from Santa Catarina and Rio Grande do Sul by Drake and Plaumann (1955) and Rodrigues et al. (2012).

#### Microvelia hamadae

dos Santos, Rodrigues, Couceiro & Moreira, 2021

5CAA31C5-F9A3-5A23-B621-EFD969C07BF8

##### Materials

- **Type status:** Other material. **Occurrence:** recordedBy: C. F. B. Floriano; individualCount: 18; sex: male; lifeStage: adult; occurrenceID: 16F9F2AF-6EA9-5775-A7DF-86FCE6F10FA5; **Taxon:** genus: Microvelia; specificEpithet: hamadae; taxonRank: species; scientificNameAuthorship: dos Santos, Rodrigues, Couceiro & Moreira, 2021; **Location:** stateProvince: Paraná; municipality: Tuneiras do Oeste; locality: Reserva Biológica das Perobas, brejo na trilha; decimalLatitude: -23.8883; decimalLongitude: -52.8086; **Identification:** identificationID: Microvelia hamadae; identifiedBy: C. F. B. Floriano; **Event:** samplingProtocol: "D" Net; year: 2024; month: IV; **Record Level:** institutionCode: CEIOC; basisOfRecord: PreservedSpecimen
- **Type status:** Other material. **Occurrence:** recordedBy: C. F. B. Floriano; individualCount: 21; sex: female; lifeStage: adult; occurrenceID: B70C5EDF-1153-5F95-BA7B-C357881BFE0F; **Taxon:** genus: Microvelia; specificEpithet: hamadae; taxonRank: species; scientificNameAuthorship: dos Santos, Rodrigues, Couceiro & Moreira, 2021; **Location:** stateProvince: Paraná; municipality: Tuneiras do Oeste; locality: Reserva Biológica das Perobas, brejo na trilha; decimalLatitude: -23.8883; decimalLongitude: -52.8086; **Identification:** identificationID: Microvelia hamadae; identifiedBy: C. F. B. Floriano; **Event:** samplingProtocol: "D" Net; year: 2024; month: IV; **Record Level:** institutionCode: CEIOC; basisOfRecord: PreservedSpecimen
- **Type status:** Other material. **Occurrence:** recordedBy: C. F. B. Floriano; individualCount: 1; sex: male; lifeStage: adult; occurrenceID: 291290EA-96CF-5D85-B7C3-D58FC2FB10AF; **Taxon:** genus: Microvelia; specificEpithet: hamadae; taxonRank: species; scientificNameAuthorship: dos Santos, Rodrigues, Couceiro & Moreira, 2021; **Location:** stateProvince: Paraná; municipality: Tuneiras do Oeste; locality: Reserva Biológica das Perobas; decimalLatitude: -23.8797; decimalLongitude: -52.8194; **Identification:** identificationID: Microvelia hamadae; identifiedBy: C. F. B. Floriano; **Event:** samplingProtocol: "D" Net; year: 2024; month: IV; **Record Level:** institutionCode: CEIOC; basisOfRecord: PreservedSpecimen

##### Distribution

Brazil.

##### Notes

First records from Paraná and the South Region of Brazil (Fig. 3B, Fig. 4B).

#### Microvelia hambletoni

Drake, 1951

FFC0426B-9C63-55AC-9BD5-77D0D8D6AF05

##### Materials

- **Type status:** Other material. **Occurrence:** recordedBy: C. F. B. Floriano & T. Malacarne; individualCount: 19; sex: male; lifeStage: adult; occurrenceID: A133A7E6-8441-507F-A4C7-5F451036E34D; **Taxon:** genus: Microvelia; specificEpithet: hambletoni; taxonRank: species; scientificNameAuthorship: Drake, 1951; **Location:** stateProvince: Santa Catarina; municipality: Blumenau; locality: Parque Nacional da Serra do Itajaí, Parque Natural Municipal Nascentes do Garcia, Rio Garcia; decimalLatitude: -27.0538; decimalLongitude: -49.0863; **Identification:** identificationID: Microvelia hambletoni; identifiedBy: C. F. B. Floriano; **Event:** samplingProtocol: "D" Net; year: 2017; month: IV; **Record Level:** institutionCode: CEIOC; basisOfRecord: PreservedSpecimen
- **Type status:** Other material. **Occurrence:** recordedBy: C. F. B. Floriano & T. Malacarne; individualCount: 26; sex: female; lifeStage: adult; occurrenceID: 38D5D8AE-2A70-5B0F-97A0-1659FF4616E7; **Taxon:** genus: Microvelia; specificEpithet: hambletoni; taxonRank: species; scientificNameAuthorship: Drake, 1951; **Location:** stateProvince: Santa Catarina; municipality: Blumenau; locality: Parque Nacional da Serra do Itajaí, Parque Natural Municipal Nascentes do Garcia, Rio Garcia; decimalLatitude: -27.0538; decimalLongitude: -49.0863; **Identification:** identificationID: Microvelia hambletoni; identifiedBy: C. F. B. Floriano; **Event:** samplingProtocol: "D" Net; year: 2017; month: IV; **Record Level:** institutionCode: CEIOC; basisOfRecord: PreservedSpecimen
- **Type status:** Other material. **Occurrence:** recordedBy: C. F. B. Floriano & T. Malacarne; individualCount: 5; sex: male; lifeStage: adult; occurrenceID: 296986AB-5C3F-515D-A97C-AA48D7736D4B; **Taxon:** genus: Microvelia; specificEpithet: hambletoni; taxonRank: species; scientificNameAuthorship: Drake, 1951; **Location:** stateProvince: Santa Catarina; municipality: Blumenau; locality: Parque Nacional da Serra do Itajaí, Parque Natural Municipal Nascentes do Garcia, Curva do Rio Garcia; decimalLatitude: -27.0610; decimalLongitude: -49.0951; **Identification:** identificationID: Microvelia hambletoni; identifiedBy: C. F. B. Floriano; **Event:** samplingProtocol: "D" Net; year: 2017; month: IV; **Record Level:** institutionCode: CEIOC; basisOfRecord: PreservedSpecimen
- **Type status:** Other material. **Occurrence:** recordedBy: C. F. B. Floriano & T. Malacarne; individualCount: 8; sex: female; lifeStage: adult; occurrenceID: D4EAA63A-4B4A-543D-A440-198462DF056A; **Taxon:** genus: Microvelia; specificEpithet: hambletoni; taxonRank: species; scientificNameAuthorship: Drake, 1951; **Location:** stateProvince: Santa Catarina; municipality: Blumenau; locality: Parque Nacional da Serra do Itajaí, Parque Natural Municipal Nascentes do Garcia, Curva do Rio Garcia; decimalLatitude: -27.0610; decimalLongitude: -49.0951; **Identification:** identificationID: Microvelia hambletoni; identifiedBy: C. F. B. Floriano; **Event:** samplingProtocol: "D" Net; year: 2017; month: IV; **Record Level:** institutionCode: CEIOC; basisOfRecord: PreservedSpecimen
- **Type status:** Other material. **Occurrence:** recordedBy: C. F. B. Floriano & T. Malacarne; individualCount: 2; sex: male; lifeStage: adult; occurrenceID: 2D8C599D-560A-587D-BF46-1E9719ED0D37; **Taxon:** genus: Microvelia; specificEpithet: hambletoni; taxonRank: species; scientificNameAuthorship: Drake, 1951; **Location:** stateProvince: Santa Catarina; municipality: Indaial; locality: Parque Nacional da Serra do Itajaí, Pesque Pague do Wili; decimalLatitude: -27.0158; decimalLongitude: -49.1660; **Identification:** identificationID: Microvelia hambletoni; identifiedBy: C. F. B. Floriano; **Event:** samplingProtocol: "D" Net; year: 2017; month: IV; **Record Level:** institutionCode: CEIOC; basisOfRecord: PreservedSpecimen
- **Type status:** Other material. **Occurrence:** recordedBy: C. F. B. Floriano & T. Malacarne; individualCount: 5; sex: female; lifeStage: adult; occurrenceID: 3779911C-9E18-5F1E-B6F9-ABC49016315B; **Taxon:** genus: Microvelia; specificEpithet: hambletoni; taxonRank: species; scientificNameAuthorship: Drake, 1951; **Location:** stateProvince: Santa Catarina; municipality: Indaial; locality: Parque Nacional da Serra do Itajaí, Pesque Pague do Wili; decimalLatitude: -27.0158; decimalLongitude: -49.1660; **Identification:** identificationID: Microvelia hambletoni; identifiedBy: C. F. B. Floriano; **Event:** samplingProtocol: "D" Net; year: 2017; month: IV; **Record Level:** institutionCode: CEIOC; basisOfRecord: PreservedSpecimen
- **Type status:** Other material. **Occurrence:** recordedBy: C. F. B. Floriano & T. Polizei; individualCount: 1; sex: male; lifeStage: adult; occurrenceID: 4DDFAC2A-0C09-5F23-B2CF-CA78C2B82EB6; **Taxon:** genus: Microvelia; specificEpithet: hambletoni; taxonRank: species; scientificNameAuthorship: Drake, 1951; **Location:** stateProvince: Santa Catarina; municipality: São Bonifácio; locality: Rio Broemer; decimalLatitude: -27.89; decimalLongitude: -48.92; **Identification:** identificationID: Microvelia hambletoni; identifiedBy: C. F. B. Floriano; **Event:** samplingProtocol: "D" Net; year: 2016; month: IX; **Record Level:** institutionCode: CEIOC; basisOfRecord: PreservedSpecimen
- **Type status:** Other material. **Occurrence:** recordedBy: C. F. B. Floriano & T. Polizei; individualCount: 1; sex: female; lifeStage: adult; occurrenceID: CAE79DED-3049-5F58-ABDF-C2056055DC8C; **Taxon:** genus: Microvelia; specificEpithet: hambletoni; taxonRank: species; scientificNameAuthorship: Drake, 1951; **Location:** stateProvince: Santa Catarina; municipality: São Bonifácio; locality: Rio Broemer; decimalLatitude: -27.89; decimalLongitude: -48.92; **Identification:** identificationID: Microvelia hambletoni; identifiedBy: C. F. B. Floriano; **Event:** samplingProtocol: "D" Net; year: 2016; month: IX; **Record Level:** institutionCode: CEIOC; basisOfRecord: PreservedSpecimen
- **Type status:** Other material. **Occurrence:** recordedBy: C. F. B. Floriano & T. Polizei; individualCount: 3; sex: male; lifeStage: adult; occurrenceID: CBF14CE5-AC31-5307-AE3F-62E249988CFA; **Taxon:** genus: Microvelia; specificEpithet: hambletoni; taxonRank: species; scientificNameAuthorship: Drake, 1951; **Location:** stateProvince: Rio Grande do Sul; municipality: Cambará do Sul; locality: Parque Nacional de Aparados da Serra, Córrego Preá; decimalLatitude: -29.17; decimalLongitude: -50.09; **Identification:** identificationID: Microvelia hambletoni; identifiedBy: C. F. B. Floriano; **Event:** samplingProtocol: "D" Net; year: 2016; month: IX; **Record Level:** institutionCode: CEIOC; basisOfRecord: PreservedSpecimen
- **Type status:** Other material. **Occurrence:** recordedBy: C. F. B. Floriano & T. Polizei; individualCount: 19; sex: female; lifeStage: adult; occurrenceID: 8E50284F-6524-5C00-99DE-7C8AF7B68D96; **Taxon:** genus: Microvelia; specificEpithet: hambletoni; taxonRank: species; scientificNameAuthorship: Drake, 1951; **Location:** stateProvince: Rio Grande do Sul; municipality: Cambará do Sul; locality: Parque Nacional de Aparados da Serra, Córrego Preá; decimalLatitude: -29.17; decimalLongitude: -50.09; **Identification:** identificationID: Microvelia hambletoni; identifiedBy: C. F. B. Floriano; **Event:** samplingProtocol: "D" Net; year: 2016; month: IX; **Record Level:** institutionCode: CEIOC; basisOfRecord: PreservedSpecimen
- **Type status:** Other material. **Occurrence:** recordedBy: C. F. B. Floriano & T. Polizei; individualCount: 2; sex: male; lifeStage: adult; occurrenceID: 6C1438D5-9C76-59F4-9714-FD44E0D4E2F1; **Taxon:** genus: Microvelia; specificEpithet: hambletoni; taxonRank: species; scientificNameAuthorship: Drake, 1951; **Location:** stateProvince: Rio Grande do Sul; municipality: Cambará do Sul; locality: Parque Nacional de Aparados da Serra, Córrego Marcel; decimalLatitude: -29.17; decimalLongitude: -50.09; **Identification:** identificationID: Microvelia hambletoni; identifiedBy: C. F. B. Floriano; **Event:** samplingProtocol: "D" Net; year: 2016; month: IX; **Record Level:** institutionCode: CEIOC; basisOfRecord: PreservedSpecimen
- **Type status:** Other material. **Occurrence:** recordedBy: C. F. B. Floriano & T. Polizei; individualCount: 4; sex: female; lifeStage: adult; occurrenceID: 6DAFCB3A-5F58-518F-8111-1F5DEE28C11A; **Taxon:** genus: Microvelia; specificEpithet: hambletoni; taxonRank: species; scientificNameAuthorship: Drake, 1951; **Location:** stateProvince: Rio Grande do Sul; municipality: Cambará do Sul; locality: Parque Nacional de Aparados da Serra, Córrego Marcel; decimalLatitude: -29.17; decimalLongitude: -50.09; **Identification:** identificationID: Microvelia hambletoni; identifiedBy: C. F. B. Floriano; **Event:** samplingProtocol: "D" Net; year: 2016; month: IX; **Record Level:** institutionCode: CEIOC; basisOfRecord: PreservedSpecimen
- **Type status:** Other material. **Occurrence:** recordedBy: C. F. B. Floriano; individualCount: 4; sex: male; lifeStage: adult; occurrenceID: A7609B88-5F62-5A9D-AA49-E471C78FA76A; **Taxon:** genus: Microvelia; specificEpithet: hambletoni; taxonRank: species; scientificNameAuthorship: Drake, 1951; **Location:** stateProvince: Paraná; municipality: Tuneiras do Oeste; locality: Reserva Biológica das Perobas, Rio Mouro; decimalLatitude: -23.8850; decimalLongitude: -52.8072; **Identification:** identificationID: Microvelia hambletoni; identifiedBy: C. F. B. Floriano; **Event:** samplingProtocol: "D" Net; year: 2024; month: IV; **Record Level:** institutionCode: CEIOC; basisOfRecord: PreservedSpecimen
- **Type status:** Other material. **Occurrence:** recordedBy: C. F. B. Floriano; individualCount: 2; sex: female; lifeStage: adult; occurrenceID: A62B9B7F-A2E1-50F2-BC55-0F66C3314E27; **Taxon:** genus: Microvelia; specificEpithet: hambletoni; taxonRank: species; scientificNameAuthorship: Drake, 1951; **Location:** stateProvince: Paraná; municipality: Tuneiras do Oeste; locality: Reserva Biológica das Perobas, Rio Mouro; decimalLatitude: -23.8850; decimalLongitude: -52.8072; **Identification:** identificationID: Microvelia hambletoni; identifiedBy: C. F. B. Floriano; **Event:** samplingProtocol: "D" Net; year: 2024; month: IV; **Record Level:** institutionCode: CEIOC; basisOfRecord: PreservedSpecimen
- **Type status:** Other material. **Occurrence:** recordedBy: C. F. B. Floriano; individualCount: 1; sex: male; lifeStage: adult; occurrenceID: AE0D4C87-17B0-5CC7-8D57-C822EAA366C0; **Taxon:** genus: Microvelia; specificEpithet: hambletoni; taxonRank: species; scientificNameAuthorship: Drake, 1951; **Location:** stateProvince: Paraná; municipality: Tuneiras do Oeste; locality: Reserva Biológica das Perobas; decimalLatitude: -23.8797; decimalLongitude: -52.8194; **Identification:** identificationID: Microvelia hambletoni; identifiedBy: C. F. B. Floriano; **Event:** samplingProtocol: "D" Net; year: 2024; month: IV; **Record Level:** institutionCode: CEIOC; basisOfRecord: PreservedSpecimen
- **Type status:** Other material. **Occurrence:** recordedBy: C. F. B. Floriano; individualCount: 3; sex: male; lifeStage: adult; occurrenceID: 05F1D701-6012-582C-A365-3BAF55C8041E; **Taxon:** genus: Microvelia; specificEpithet: hambletoni; taxonRank: species; scientificNameAuthorship: Drake, 1951; **Location:** stateProvince: Paraná; municipality: Cianorte; locality: Reserva Biológica das Perobas, Córrego Adelaide; decimalLatitude: -23.8472; decimalLongitude: -52.7389; **Identification:** identificationID: Microvelia hambletoni; identifiedBy: C. F. B. Floriano; **Event:** samplingProtocol: "D" Net; year: 2024; month: IV; **Record Level:** institutionCode: CEIOC; basisOfRecord: PreservedSpecimen
- **Type status:** Other material. **Occurrence:** recordedBy: C. F. B. Floriano; individualCount: 5; sex: female; lifeStage: adult; occurrenceID: 6B9A0E4E-7609-5BA2-B6EE-5D5FB2B52503; **Taxon:** genus: Microvelia; specificEpithet: hambletoni; taxonRank: species; scientificNameAuthorship: Drake, 1951; **Location:** stateProvince: Paraná; municipality: Cianorte; locality: Reserva Biológica das Perobas, Córrego Adelaide; decimalLatitude: -23.8472; decimalLongitude: -52.7389; **Identification:** identificationID: Microvelia hambletoni; identifiedBy: C. F. B. Floriano; **Event:** samplingProtocol: "D" Net; year: 2024; month: IV; **Record Level:** institutionCode: CEIOC; basisOfRecord: PreservedSpecimen

##### Distribution

Brazil and Peru.

##### Notes

First records from Paraná and Rio Grande do Sul (Fig. 3C, Fig. 4B). Previously recorded from Santa Catarina (Drake 1951).

#### Microvelia hinei

Drake, 1920

B5ED3720-0058-5E8D-BF3E-91C59A377F61

##### Materials

- **Type status:** Other material. **Occurrence:** recordedBy: C. F. B. Floriano; individualCount: 1; sex: male; lifeStage: adult; occurrenceID: 75894A8F-1531-5366-9384-550EB4A31538; **Taxon:** genus: Microvelia; specificEpithet: hinei; taxonRank: species; scientificNameAuthorship: Drake, 1920; **Location:** stateProvince: Paraná; municipality: Tuneiras do Oeste; locality: Reserva Biológica das Perobas, Lagoa; decimalLatitude: -23.8894; decimalLongitude: -52.8144; **Identification:** identificationID: Microvelia hinei; identifiedBy: C. F. B. Floriano; **Event:** samplingProtocol: "D" Net; year: 2024; month: IV; **Record Level:** institutionCode: CEIOC; basisOfRecord: PreservedSpecimen

##### Distribution

Canada, United States, Mexico, Dominan Republic, Puerto Rico, Brazil, Peru and Argentina.

##### Notes

First record from Paraná and the South Region of Brazil (Fig. 3D, Fig. 4C).

#### Microvelia ioana

Drake & Hottes, 1952

2F3BA36E-1F27-5C65-993D-CB60A50D7426

##### Materials

- **Type status:** Other material. **Occurrence:** recordedBy: C. F. B. Floriano & T. Malacarne; individualCount: 4; sex: male; lifeStage: adult; occurrenceID: 34AED6E5-78D5-5962-9AB6-DAF8A8D917F0; **Taxon:** genus: Microvelia; specificEpithet: ioana; taxonRank: species; scientificNameAuthorship: Drake & Hottes, 1952; **Location:** stateProvince: Santa Catarina; municipality: Blumenau; locality: Parque Nacional da Serra do Itajaí, Parque Natural Municipal Nascentes do Garcia, Cachoeira da captação; decimalLatitude: -27.0545; decimalLongitude: -49.0838; **Identification:** identificationID: Microvelia ioana; identifiedBy: C. F. B. Floriano; **Event:** samplingProtocol: "D" Net; year: 2017; month: IV; **Record Level:** institutionCode: CEIOC; basisOfRecord: PreservedSpecimen
- **Type status:** Other material. **Occurrence:** recordedBy: C. F. B. Floriano & T. Malacarne; individualCount: 2; sex: female; lifeStage: adult; occurrenceID: 96E7F1DE-2F34-5944-91F8-B44D4A37BA9E; **Taxon:** genus: Microvelia; specificEpithet: ioana; taxonRank: species; scientificNameAuthorship: Drake & Hottes, 1952; **Location:** stateProvince: Santa Catarina; municipality: Blumenau; locality: Parque Nacional da Serra do Itajaí, Parque Natural Municipal Nascentes do Garcia, Cachoeira da captação; decimalLatitude: -27.0545; decimalLongitude: -49.0838; **Identification:** identificationID: Microvelia ioana; identifiedBy: C. F. B. Floriano; **Event:** samplingProtocol: "D" Net; year: 2017; month: IV; **Record Level:** institutionCode: CEIOC; basisOfRecord: PreservedSpecimen
- **Type status:** Other material. **Occurrence:** recordedBy: F. Plaumann; individualCount: 3; sex: male; lifeStage: adult; occurrenceID: A6E64681-59CB-5B98-BAA3-11B56EAE0C27; **Taxon:** genus: Microvelia; specificEpithet: ioana; taxonRank: species; scientificNameAuthorship: Drake & Hottes, 1952; **Location:** stateProvince: Rio Grande do Sul; municipality: Candelária; locality: Botucaraí; decimalLatitude: -29.71; decimalLongitude: -52.91; **Identification:** identificationID: Microvelia ioana; identifiedBy: C. F. B. Floriano; **Event:** samplingProtocol: "D" Net; year: 1959; month: XI; **Record Level:** institutionCode: MZUSP; basisOfRecord: PreservedSpecimen
- **Type status:** Other material. **Occurrence:** recordedBy: F. Plaumann; individualCount: 3; sex: female; lifeStage: adult; occurrenceID: 01EA9F6D-FF5C-56AA-99C3-FA6F36131378; **Taxon:** genus: Microvelia; specificEpithet: ioana; taxonRank: species; scientificNameAuthorship: Drake & Hottes, 1952; **Location:** stateProvince: Rio Grande do Sul; municipality: Candelária; locality: Botucaraí; decimalLatitude: -29.71; decimalLongitude: -52.91; **Identification:** identificationID: Microvelia ioana; identifiedBy: C. F. B. Floriano; **Event:** samplingProtocol: "D" Net; year: 1959; month: XI; **Record Level:** institutionCode: MZUSP; basisOfRecord: PreservedSpecimen
- **Type status:** Other material. **Occurrence:** recordedBy: F. Plaumann; individualCount: 5; sex: male; lifeStage: adult; occurrenceID: 50DACB63-6D16-591D-AAC6-623E61FFC289; **Taxon:** genus: Microvelia; specificEpithet: ioana; taxonRank: species; scientificNameAuthorship: Drake & Hottes, 1952; **Location:** stateProvince: Rio Grande do Sul; municipality: Nova Petrópolis; decimalLatitude: -29.36; decimalLongitude: -51.20; **Identification:** identificationID: Microvelia ioana; identifiedBy: C. F. B. Floriano; **Event:** samplingProtocol: "D" Net; year: 1959; month: XI; **Record Level:** institutionCode: MZUSP; basisOfRecord: PreservedSpecimen
- **Type status:** Other material. **Occurrence:** recordedBy: F. Plaumann; individualCount: 4; sex: female; lifeStage: adult; occurrenceID: ECC6E86B-F5DB-51E1-8205-5BCCCBB8E2AB; **Taxon:** genus: Microvelia; specificEpithet: ioana; taxonRank: species; scientificNameAuthorship: Drake & Hottes, 1952; **Location:** stateProvince: Rio Grande do Sul; municipality: Nova Petrópolis; decimalLatitude: -29.36; decimalLongitude: -51.20; **Identification:** identificationID: Microvelia ioana; identifiedBy: C. F. B. Floriano; **Event:** samplingProtocol: "D" Net; year: 1959; month: XI; **Record Level:** institutionCode: MZUSP; basisOfRecord: PreservedSpecimen

##### Distribution

Brazil.

##### Notes

First records from Rio Grande do Sul (Fig. 3E, Fig. 4C). Previously reported from Santa Catarina by Magalhães et al. (2021).

#### Microvelia limaiana

Drake, 1951

7CD72F3C-15F5-53FF-937B-3F7A106AC520

##### Materials

- **Type status:** Other material. **Occurrence:** recordedBy: C. F. B. Floriano; individualCount: 2; sex: male; lifeStage: adult; occurrenceID: 4A195F92-0329-5666-9CE8-E1858E10933D; **Taxon:** genus: Microvelia; specificEpithet: limaiana; taxonRank: species; scientificNameAuthorship: Drake, 1951; **Location:** stateProvince: Paraná; municipality: Faxinal; locality: Cachoeira do Rio São Pedro; decimalLatitude: -23.9756; decimalLongitude: -51.2247; **Identification:** identificationID: Microvelia limaiana; identifiedBy: C. F. B. Floriano; **Event:** samplingProtocol: "D" Net; year: 2020; month: IX; **Record Level:** institutionCode: CEIOC; basisOfRecord: PreservedSpecimen
- **Type status:** Other material. **Occurrence:** recordedBy: C. F. B. Floriano; individualCount: 3; sex: female; lifeStage: adult; occurrenceID: FDCCF3A0-22AE-583C-80FB-89CBA166917E; **Taxon:** genus: Microvelia; specificEpithet: limaiana; taxonRank: species; scientificNameAuthorship: Drake, 1951; **Location:** stateProvince: Paraná; municipality: Faxinal; locality: Cachoeira do Rio São Pedro; decimalLatitude: -23.9756; decimalLongitude: -51.2247; **Identification:** identificationID: Microvelia limaiana; identifiedBy: C. F. B. Floriano; **Event:** samplingProtocol: "D" Net; year: 2020; month: IX; **Record Level:** institutionCode: CEIOC; basisOfRecord: PreservedSpecimen
- **Type status:** Other material. **Occurrence:** recordedBy: C. F. B. Floriano & T. Polizei; individualCount: 13; sex: male; lifeStage: adult; occurrenceID: 02943E73-4A66-5CEA-B731-2BFB48324C11; **Taxon:** genus: Microvelia; specificEpithet: limaiana; taxonRank: species; scientificNameAuthorship: Drake, 1951; **Location:** stateProvince: Santa Catarina; municipality: Orleans; locality: Morro da Igreja; decimalLatitude: -28.1286; decimalLongitude: -49.4760; **Identification:** identificationID: Microvelia limaiana; identifiedBy: C. F. B. Floriano; **Event:** samplingProtocol: "D" Net; year: 2016; month: IX; **Record Level:** institutionCode: CEIOC; basisOfRecord: PreservedSpecimen
- **Type status:** Other material. **Occurrence:** recordedBy: C. F. B. Floriano & T. Polizei; individualCount: 2; sex: female; lifeStage: adult; occurrenceID: 1BBD1887-17AC-5FA4-845A-E8C11021AAB3; **Taxon:** genus: Microvelia; specificEpithet: limaiana; taxonRank: species; scientificNameAuthorship: Drake, 1951; **Location:** stateProvince: Santa Catarina; municipality: Orleans; locality: Morro da Igreja; decimalLatitude: -28.1286; decimalLongitude: -49.4760; **Identification:** identificationID: Microvelia limaiana; identifiedBy: C. F. B. Floriano; **Event:** samplingProtocol: "D" Net; year: 2016; month: IX; **Record Level:** institutionCode: CEIOC; basisOfRecord: PreservedSpecimen
- **Type status:** Other material. **Occurrence:** recordedBy: F. Plaumann; individualCount: 2; sex: male; lifeStage: adult; occurrenceID: 4E34CC98-18E3-52C8-92FD-B310DC738BE8; **Taxon:** genus: Microvelia; specificEpithet: limaiana; taxonRank: species; scientificNameAuthorship: Drake, 1951; **Location:** stateProvince: Santa Catarina; municipality: Seara; locality: Nova Teutônia, Saltinho Bach; decimalLatitude: -27.18; decimalLongitude: -52.38; **Identification:** identificationID: Microvelia limaiana; identifiedBy: C. F. B. Floriano; **Record Level:** institutionCode: MZUSP; basisOfRecord: PreservedSpecimen
- **Type status:** Other material. **Occurrence:** recordedBy: F. Plaumann; individualCount: 3; sex: female; lifeStage: adult; occurrenceID: 9E7D75F4-8065-5C62-8533-C9003A6A3653; **Taxon:** genus: Microvelia; specificEpithet: limaiana; taxonRank: species; scientificNameAuthorship: Drake, 1951; **Location:** stateProvince: Santa Catarina; municipality: Seara; locality: Nova Teutônia, Saltinho Bach; decimalLatitude: -27.18; decimalLongitude: -52.38; **Identification:** identificationID: Microvelia limaiana; identifiedBy: C. F. B. Floriano; **Record Level:** institutionCode: MZUSP; basisOfRecord: PreservedSpecimen
- **Type status:** Other material. **Occurrence:** recordedBy: F. Plaumann; individualCount: 1; sex: male; lifeStage: adult; occurrenceID: 72048F59-1B8A-5428-9324-C6CB6121C588; **Taxon:** genus: Microvelia; specificEpithet: limaiana; taxonRank: species; scientificNameAuthorship: Drake, 1951; **Location:** stateProvince: Santa Catarina; municipality: Concórdia; locality: Comunidade Cachimbó; verbatimElevation: 600 m; decimalLatitude: -27.16; decimalLongitude: -51.86; **Identification:** identificationID: Microvelia limaiana; identifiedBy: C. F. B. Floriano; **Event:** year: 1959; month: XII; **Record Level:** institutionCode: MZUSP; basisOfRecord: PreservedSpecimen
- **Type status:** Other material. **Occurrence:** recordedBy: F. Plaumann; individualCount: 3; sex: female; lifeStage: adult; occurrenceID: 11BD9D4D-B638-5D00-A66C-69E2900108DD; **Taxon:** genus: Microvelia; specificEpithet: limaiana; taxonRank: species; scientificNameAuthorship: Drake, 1951; **Location:** stateProvince: Santa Catarina; municipality: Concórdia; locality: Comunidade Cachimbó; decimalLatitude: -27.16; decimalLongitude: -51.86; **Identification:** identificationID: Microvelia limaiana; identifiedBy: C. F. B. Floriano; **Event:** year: 1959; month: XII; **Record Level:** institutionCode: MZUSP; basisOfRecord: PreservedSpecimen

##### Distribution

Brazil and Argentina.

##### Notes

First records from Paraná (Fig. 3F, Fig. 4C). Originally described from Santa Catarina (Drake 1951).

#### Microvelia longipes

Uhler, 1894

D326112D-B36B-50C2-8C05-C5BBCF85D95A

##### Materials

- **Type status:** Other material. **Occurrence:** recordedBy: C. F. B. Floriano; individualCount: 1; sex: male; lifeStage: adult; occurrenceID: 2D3154FE-4E9F-5CFF-AC23-AD37C25C6760; **Taxon:** genus: Microvelia; specificEpithet: longipes; taxonRank: species; scientificNameAuthorship: Uhler, 1894; **Location:** stateProvince: Paraná; municipality: Paranaguá; locality: Ilha do Mel, pousada Ephira; decimalLatitude: -25.5656; decimalLongitude: -48.3153; **Identification:** identificationID: Microvelia longipes; identifiedBy: C. F. B. Floriano; **Event:** samplingProtocol: "D" Net; year: 2016; month: X; **Record Level:** institutionCode: CEIOC; basisOfRecord: PreservedSpecimen
- **Type status:** Other material. **Occurrence:** recordedBy: C. F. B. Floriano; individualCount: 2; sex: male; lifeStage: adult; occurrenceID: 127E87DE-A174-5911-B442-0090E8449E01; **Taxon:** genus: Microvelia; specificEpithet: longipes; taxonRank: species; scientificNameAuthorship: Uhler, 1894; **Location:** stateProvince: Paraná; municipality: Tuneiras do Oeste; locality: Reserva Biológica das Perobas, poça na estrada; decimalLatitude: -23.8553; decimalLongitude: -52.8114; **Identification:** identificationID: Microvelia longipes; identifiedBy: C. F. B. Floriano; **Event:** samplingProtocol: "D" Net; year: 2024; month: IV; **Record Level:** institutionCode: CEIOC; basisOfRecord: PreservedSpecimen
- **Type status:** Other material. **Occurrence:** recordedBy: C. F. B. Floriano; individualCount: 15; sex: male; lifeStage: adult; occurrenceID: 858CB341-1FAA-5DF1-8AC3-3E3EC2C5F80A; **Taxon:** genus: Microvelia; specificEpithet: longipes; taxonRank: species; scientificNameAuthorship: Uhler, 1894; **Location:** stateProvince: Paraná; municipality: Tuneiras do Oeste; locality: Reserva Biológica das Perobas, poça na estrada; decimalLatitude: -23.8983; decimalLongitude: -52.7439; **Identification:** identificationID: Microvelia longipes; identifiedBy: C. F. B. Floriano; **Event:** samplingProtocol: "D" Net; year: 2024; month: IV; **Record Level:** institutionCode: CEIOC; basisOfRecord: PreservedSpecimen
- **Type status:** Other material. **Occurrence:** recordedBy: C. F. B. Floriano; individualCount: 6; sex: female; lifeStage: adult; occurrenceID: 9E7ED954-F440-5FEF-9A6B-694CFBC02C59; **Taxon:** genus: Microvelia; specificEpithet: longipes; taxonRank: species; scientificNameAuthorship: Uhler, 1894; **Location:** stateProvince: Paraná; municipality: Tuneiras do Oeste; locality: Reserva Biológica das Perobas, poça na estrada; decimalLatitude: -23.8983; decimalLongitude: -52.7439; **Identification:** identificationID: Microvelia longipes; identifiedBy: C. F. B. Floriano; **Event:** samplingProtocol: "D" Net; year: 2024; month: IV; **Record Level:** institutionCode: CEIOC; basisOfRecord: PreservedSpecimen

##### Distribution

Cuba, Dominican Republic, Jamaica, Puerto Rico, U. S. Virgin Islands, St. Martin, Saint Barthélemy, St. Eustatius, St. Kitts & Nevis, Aruba, Barbados, Curaçao, Bonaire, Grenada, Trinidad & Tobago, Colombia, Venezuela, Guyana, French Guiana, Brazil, Ecuador, Peru, Bolivia, Paraguay and Argentina.

##### Notes

First record from Paraná (Fig. 3G, Fig. 4C). Previously recorded from Santa Catarina (Drake and Plaumann 1955).

#### Microvelia lujanana

Drake, 1951

775F8666-DE01-58CB-AC64-C8F83CCF0472

##### Materials

- **Type status:** Other material. **Occurrence:** recordedBy: F. Plaumann; individualCount: 3; sex: male; lifeStage: adult; occurrenceID: 90592ABF-2F82-5002-A60C-79C5BBD47180; **Taxon:** genus: Microvelia; specificEpithet: lujanana; taxonRank: species; scientificNameAuthorship: Drake, 1951; **Location:** stateProvince: Rio Grande do Sul; municipality: Erechim; decimalLatitude: -27.64; decimalLongitude: -52.27; **Identification:** identificationID: Microvelia lujanana; identifiedBy: C. F. B. Floriano; **Event:** samplingProtocol: "D" Net; year: 1957; month: V; **Record Level:** institutionCode: NMNH; basisOfRecord: PreservedSpecimen
- **Type status:** Other material. **Occurrence:** recordedBy: F. Plaumann; individualCount: 3; sex: female; lifeStage: adult; occurrenceID: 1A7A11F9-57B0-5116-AF11-5F6F3D2B5B3C; **Taxon:** genus: Microvelia; specificEpithet: lujanana; taxonRank: species; scientificNameAuthorship: Drake, 1951; **Location:** stateProvince: Rio Grande do Sul; municipality: Erechim; decimalLatitude: -27.64; decimalLongitude: -52.27; **Identification:** identificationID: Microvelia lujanana; identifiedBy: C. F. B. Floriano; **Event:** samplingProtocol: "D" Net; year: 1957; month: V; **Record Level:** institutionCode: NMNH; basisOfRecord: PreservedSpecimen
- **Type status:** Other material. **Occurrence:** recordedBy: C. F. B. Floriano & T. Polizei; individualCount: 1; sex: male; lifeStage: adult; occurrenceID: 30128170-2821-58B4-9791-75CFEC906D07; **Taxon:** genus: Microvelia; specificEpithet: lujanana; taxonRank: species; scientificNameAuthorship: Drake, 1951; **Location:** stateProvince: Rio Grande do Sul; municipality: Cambará do Sul; decimalLatitude: -29.047; decimalLongitude: -50.145; **Identification:** identificationID: Microvelia lujanana; identifiedBy: C. F. B. Floriano; **Event:** samplingProtocol: "D" Net; year: 2016; month: IX; **Record Level:** institutionCode: CEIOC; basisOfRecord: PreservedSpecimen
- **Type status:** Other material. **Occurrence:** recordedBy: C. F. B. Floriano & T. Polizei; individualCount: 3; sex: female; lifeStage: adult; occurrenceID: 37917A55-908F-5941-9406-201F3FC32951; **Taxon:** genus: Microvelia; specificEpithet: lujanana; taxonRank: species; scientificNameAuthorship: Drake, 1951; **Location:** stateProvince: Rio Grande do Sul; municipality: Cambará do Sul; decimalLatitude: -29.047; decimalLongitude: -50.145; **Identification:** identificationID: Microvelia lujanana; identifiedBy: C. F. B. Floriano; **Event:** samplingProtocol: "D" Net; year: 2016; month: IX; **Record Level:** institutionCode: CEIOC; basisOfRecord: PreservedSpecimen
- **Type status:** Other material. **Occurrence:** recordedBy: F. Plaumann; individualCount: 4; sex: male; lifeStage: adult; occurrenceID: 4C0F6AEE-EC72-5DCF-AA44-400BAFA8D8A5; **Taxon:** genus: Microvelia; specificEpithet: lujanana; taxonRank: species; scientificNameAuthorship: Drake, 1951; **Location:** stateProvince: Santa Catarina; municipality: Seara; locality: Nova Teutônia; decimalLatitude: -27.18; decimalLongitude: -52.33; **Identification:** identificationID: Microvelia lujanana; identifiedBy: C. F. B. Floriano; **Event:** samplingProtocol: "D" Net; year: 1955; month: VIII; **Record Level:** institutionCode: MZUSP; basisOfRecord: PreservedSpecimen
- **Type status:** Other material. **Occurrence:** recordedBy: F. Plaumann; individualCount: 3; sex: female; lifeStage: adult; occurrenceID: 17F64EDB-8680-577D-B537-9F03785D45FC; **Taxon:** genus: Microvelia; specificEpithet: lujanana; taxonRank: species; scientificNameAuthorship: Drake, 1951; **Location:** stateProvince: Santa Catarina; municipality: Seara; locality: Nova Teutônia; decimalLatitude: -27.18; decimalLongitude: -52.33; **Identification:** identificationID: Microvelia lujanana; identifiedBy: C. F. B. Floriano; **Event:** samplingProtocol: "D" Net; year: 1955; month: VIII; **Record Level:** institutionCode: MZUSP; basisOfRecord: PreservedSpecimen

##### Distribution

Brazil and Argentina.

##### Notes

**Figure 5.**
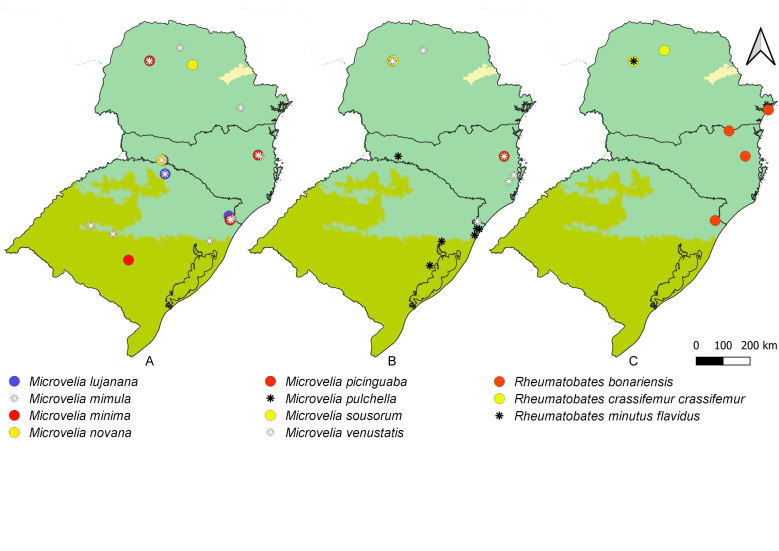
Maps showing new records of Gerridae (*Microvelia*, *Rheumatobates*) from the South Region of Brazil.

First records from Brazil, in the States of Santa Catarina and Rio Grande do Sul (Fig. 3H, Fig. 5A).

##### Diagnosis

Apterous form. Body length 1.10–1.45 mm; general colour brown/black, with areas of frosty pubescence; pronotum very short, not extended over mesonotum; male abdominal sterna II–IV slightly depressed centrally; posterior margin of male abdominal tergum VIII straight, slightly oblique; male pygophore and proctiger slightly asymmeric, right posterolateral angle of pygophore not notched, proctiger without distinct lateral processes; parameres asymmetrical, short, left larger than right.

#### Microvelia mimula

White, 1879

0202161F-9435-579B-A8B7-C0B057F289A1

##### Materials

- **Type status:** Other material. **Occurrence:** recordedBy: C. F. B. Floriano & T. Malacarne; individualCount: 1; sex: male; lifeStage: adult; occurrenceID: CE4C06DF-A687-5E7D-AD22-A665E3DEC6C5; **Taxon:** genus: Microvelia; specificEpithet: mimula; taxonRank: species; scientificNameAuthorship: White, 1879; **Location:** stateProvince: Santa Catarina; municipality: Indaial; locality: Parque Nacional da Serra do Itajaí, Pesque Pague do Wili; decimalLatitude: -27.0161; decimalLongitude: -49.1665; **Identification:** identificationID: Microvelia mimula; identifiedBy: C. F. B. Floriano; **Event:** samplingProtocol: "D" Net; year: 2017; month: IV; **Record Level:** institutionCode: CEIOC; basisOfRecord: PreservedSpecimen
- **Type status:** Other material. **Occurrence:** recordedBy: C. F. B. Floriano & T. Malacarne; individualCount: 1; sex: male; lifeStage: adult; occurrenceID: 87323F27-8BAE-5C5E-A346-4548B6F3C77A; **Taxon:** genus: Microvelia; specificEpithet: mimula; taxonRank: species; scientificNameAuthorship: White, 1879; **Location:** stateProvince: Santa Catarina; municipality: Blumenau; locality: Parque Nacional da Serra do Itajaí, Parque Natural Municipal Nascentes do Garcia, Rio Garcia; decimalLatitude: -27.0538; decimalLongitude: -49.0863; **Identification:** identificationID: Microvelia mimula; identifiedBy: C. F. B. Floriano; **Event:** samplingProtocol: "D" Net; year: 2017; month: IV; **Record Level:** institutionCode: CEIOC; basisOfRecord: PreservedSpecimen
- **Type status:** Other material. **Occurrence:** recordedBy: F. Plaumann; individualCount: 4; sex: male; lifeStage: adult; occurrenceID: DA107C14-2EDD-5B63-AAAC-EA23D49A6FED; **Taxon:** genus: Microvelia; specificEpithet: mimula; taxonRank: species; scientificNameAuthorship: White, 1879; **Location:** stateProvince: Santa Catarina; municipality: Seara; locality: Nova Teutônia; decimalLatitude: -27.19; decimalLongitude: -52.33; **Identification:** identificationID: Microvelia mimula; identifiedBy: C. F. B. Floriano; **Event:** year: 1953; month: VIII; **Record Level:** institutionCode: MZUSP; basisOfRecord: PreservedSpecimen
- **Type status:** Other material. **Occurrence:** recordedBy: F. Plaumann; individualCount: 3; sex: female; lifeStage: adult; occurrenceID: 55EB349E-5BAC-591B-A65B-8ECAD02B3850; **Taxon:** genus: Microvelia; specificEpithet: mimula; taxonRank: species; scientificNameAuthorship: White, 1879; **Location:** stateProvince: Santa Catarina; municipality: Seara; locality: Nova Teutônia; decimalLatitude: -27.19; decimalLongitude: -52.33; **Identification:** identificationID: Microvelia mimula; identifiedBy: C. F. B. Floriano; **Event:** year: 1953; month: VIII; **Record Level:** institutionCode: MZUSP; basisOfRecord: PreservedSpecimen
- **Type status:** Other material. **Occurrence:** recordedBy: C. F. B. Floriano & T. Polizei; individualCount: 6; sex: male; lifeStage: adult; occurrenceID: 863055C1-11CE-57E7-A551-0E970C3D6E15; **Taxon:** genus: Microvelia; specificEpithet: mimula; taxonRank: species; scientificNameAuthorship: White, 1879; **Location:** stateProvince: Rio Grande do Sul; municipality: Cambará do Sul; locality: Parque Nacional da Serra Geral, trilha do segredo; decimalLatitude: -29.17; decimalLongitude: -50.09; **Identification:** identificationID: Microvelia mimula; identifiedBy: C. F. B. Floriano; **Event:** samplingProtocol: "D" Net; year: 2016; month: IX; **Record Level:** institutionCode: CEIOC; basisOfRecord: PreservedSpecimen
- **Type status:** Other material. **Occurrence:** recordedBy: C. F. B. Floriano & T. Polizei; individualCount: 8; sex: female; lifeStage: adult; occurrenceID: 9FE08A22-EE6E-5B33-A492-FF9DBBBC731E; **Taxon:** genus: Microvelia; specificEpithet: mimula; taxonRank: species; scientificNameAuthorship: White, 1879; **Location:** stateProvince: Rio Grande do Sul; municipality: Cambará do Sul; locality: Parque Nacional da Serra Geral, trilha do segredo; decimalLatitude: -29.17; decimalLongitude: -50.09; **Identification:** identificationID: Microvelia mimula; identifiedBy: C. F. B. Floriano; **Event:** samplingProtocol: "D" Net; year: 2016; month: IX; **Record Level:** institutionCode: CEIOC; basisOfRecord: PreservedSpecimen
- **Type status:** Other material. **Occurrence:** recordedBy: C. F. B. Floriano & T. Polizei; individualCount: 2; sex: male; lifeStage: adult; occurrenceID: C5AD8238-5F4E-513A-83FE-2E8D0AB7156F; **Taxon:** genus: Microvelia; specificEpithet: mimula; taxonRank: species; scientificNameAuthorship: White, 1879; **Location:** stateProvince: Rio Grande do Sul; municipality: Cambará do Sul; locality: Parque Nacional da Serra Geral, córrego Marcel; decimalLatitude: -29.17; decimalLongitude: -50.09; **Identification:** identificationID: Microvelia mimula; identifiedBy: C. F. B. Floriano; **Event:** samplingProtocol: "D" Net; year: 2016; month: IX; **Record Level:** institutionCode: CEIOC; basisOfRecord: PreservedSpecimen
- **Type status:** Other material. **Occurrence:** recordedBy: C. F. B. Floriano & T. Polizei; individualCount: 4; sex: female; lifeStage: adult; occurrenceID: 7F99E08D-CFC6-533A-9448-02A59E65D115; **Taxon:** genus: Microvelia; specificEpithet: mimula; taxonRank: species; scientificNameAuthorship: White, 1879; **Location:** stateProvince: Rio Grande do Sul; municipality: Cambará do Sul; locality: Parque Nacional da Serra Geral, córrego Marcel; decimalLatitude: -29.17; decimalLongitude: -50.09; **Identification:** identificationID: Microvelia mimula; identifiedBy: C. F. B. Floriano; **Event:** samplingProtocol: "D" Net; year: 2016; month: IX; **Record Level:** institutionCode: CEIOC; basisOfRecord: PreservedSpecimen
- **Type status:** Other material. **Occurrence:** recordedBy: F. Plaumann; individualCount: 1; sex: male; lifeStage: adult; occurrenceID: C51A3A91-2A4C-583B-9517-9846292AB7EE; **Taxon:** genus: Microvelia; specificEpithet: mimula; taxonRank: species; scientificNameAuthorship: White, 1879; **Location:** stateProvince: Rio Grande do Sul; municipality: Erechim; decimalLatitude: -27.65; decimalLongitude: -52.28; **Identification:** identificationID: Microvelia mimula; identifiedBy: C. F. B. Floriano; **Record Level:** institutionCode: MZUSP; basisOfRecord: PreservedSpecimen
- **Type status:** Other material. **Occurrence:** recordedBy: J.M.S. Rodrigues, I.R.S. Cordeiro, O.M. Magalhães, E.A. Joaquim, L.N.A. Sant’Ana; individualCount: 1; sex: male; lifeStage: adult; occurrenceID: 5E63BCF4-86FA-5D81-A38B-7B5EE7D5928C; **Taxon:** genus: Microvelia; specificEpithet: mimula; taxonRank: species; scientificNameAuthorship: White, 1879; **Location:** stateProvince: Rio Grande do Sul; municipality: Santiago; locality: Ernesto Alves, alagado; decimalLatitude: -29.3720; decimalLongitude: -54.7448; **Identification:** identificationID: Microvelia mimula; identifiedBy: C. F. B. Floriano; **Event:** samplingProtocol: "D" Net; year: 2019; month: XI; **Record Level:** institutionCode: CEIOC; basisOfRecord: PreservedSpecimen
- **Type status:** Other material. **Occurrence:** recordedBy: J. M. S. Rodrigues, I. R. S. Cordeiro, O. M. Magalhães, E. A. Joaquim, L. N. A. Sant’Ana; individualCount: 2; sex: female; lifeStage: adult; occurrenceID: 24CACE02-4127-574E-9C60-A01CC47D7CD7; **Taxon:** genus: Microvelia; specificEpithet: mimula; taxonRank: species; scientificNameAuthorship: White, 1879; **Location:** stateProvince: Rio Grande do Sul; municipality: Santiago; locality: Ernesto Alves, alagado; decimalLatitude: -29.3720; decimalLongitude: -54.7448; **Identification:** identificationID: Microvelia mimula; identifiedBy: C. F. B. Floriano; **Event:** samplingProtocol: "D" Net; year: 2019; month: XI; **Record Level:** institutionCode: CEIOC; basisOfRecord: PreservedSpecimen
- **Type status:** Other material. **Occurrence:** recordedBy: J. M. S. Rodrigues, I. R. S. Cordeiro, O. M. Magalhães, E. A. Joaquim, L. N. A. Sant’Ana; individualCount: 1; sex: male; lifeStage: adult; occurrenceID: 724AB5F3-93F8-5B62-BE13-49FB8DFA3EC5; **Taxon:** genus: Microvelia; specificEpithet: mimula; taxonRank: species; scientificNameAuthorship: White, 1879; **Location:** stateProvince: Rio Grande do Sul; municipality: São Luiz Gonzaga; locality: Fazenda do Cerro, riacho; decimalLatitude: -28.2082; decimalLongitude: -54.9766; **Identification:** identificationID: Microvelia mimula; identifiedBy: C. F. B. Floriano; **Event:** samplingProtocol: "D" Net; year: 2019; month: XI; **Record Level:** institutionCode: CEIOC; basisOfRecord: PreservedSpecimen
- **Type status:** Other material. **Occurrence:** recordedBy: J. M. S. Rodrigues, I. R. S. Cordeiro, O. M. Magalhães, E. A. Joaquim, L. N. A. Sant’Ana; individualCount: 3; sex: female; lifeStage: adult; occurrenceID: CB18C930-3A27-5B16-AA7C-8FF43FF1D624; **Taxon:** genus: Microvelia; specificEpithet: mimula; taxonRank: species; scientificNameAuthorship: White, 1879; **Location:** stateProvince: Rio Grande do Sul; municipality: São Luiz Gonzaga; locality: Fazenda do Cerro, riacho; decimalLatitude: -28.2082; decimalLongitude: -54.9766; **Identification:** identificationID: Microvelia mimula; identifiedBy: C. F. B. Floriano; **Event:** samplingProtocol: "D" Net; year: 2019; month: XI; **Record Level:** institutionCode: CEIOC; basisOfRecord: PreservedSpecimen
- **Type status:** Other material. **Occurrence:** recordedBy: J. M. S. Rodrigues, I. R. S. Cordeiro, O. M. Magalhães, E. A. Joaquim, L. N. A. Sant’Ana; individualCount: 3; sex: male; lifeStage: adult; occurrenceID: 9400860B-2E57-5371-96A3-8DDE76FD0C02; **Taxon:** genus: Microvelia; specificEpithet: mimula; taxonRank: species; scientificNameAuthorship: White, 1879; **Location:** stateProvince: Rio Grande do Sul; municipality: Santa Maria; locality: Canabarro, açude; decimalLatitude: -29.64; decimalLongitude: -54.00; **Identification:** identificationID: Microvelia mimula; identifiedBy: C. F. B. Floriano; **Event:** samplingProtocol: "D" Net; year: 2019; month: XI; **Record Level:** institutionCode: CEIOC; basisOfRecord: PreservedSpecimen
- **Type status:** Other material. **Occurrence:** recordedBy: J. M. S. Rodrigues, I. R. S. Cordeiro, O. M. Magalhães, E. A. Joaquim, L. N. A. Sant’Ana; individualCount: 5; sex: female; lifeStage: adult; occurrenceID: 459C8090-C666-587F-8D99-1EC4EAC01124; **Taxon:** genus: Microvelia; specificEpithet: mimula; taxonRank: species; scientificNameAuthorship: White, 1879; **Location:** stateProvince: Rio Grande do Sul; municipality: Santa Maria; locality: Canabarro, açude; decimalLatitude: -29.64; decimalLongitude: -54.00; **Identification:** identificationID: Microvelia mimula; identifiedBy: C. F. B. Floriano; **Event:** samplingProtocol: "D" Net; year: 2019; month: XI; **Record Level:** institutionCode: CEIOC; basisOfRecord: PreservedSpecimen
- **Type status:** Other material. **Occurrence:** recordedBy: F. Plaumann; individualCount: 1; sex: male; lifeStage: adult; occurrenceID: 1B652CBB-1C5D-57CA-A138-963F0922B087; **Taxon:** genus: Microvelia; specificEpithet: mimula; taxonRank: species; scientificNameAuthorship: White, 1879; **Location:** stateProvince: Rio Grande do Sul; municipality: Glorinha; decimalLatitude: -29.90; decimalLongitude: -50.78; **Identification:** identificationID: Microvelia mimula; identifiedBy: C. F. B. Floriano; **Record Level:** institutionCode: MZUSP; basisOfRecord: PreservedSpecimen
- **Type status:** Other material. **Occurrence:** recordedBy: C. F. B. Floriano & T. Malacarne; individualCount: 1; sex: male; lifeStage: adult; occurrenceID: DAF5AF9F-AC8C-535D-AD3E-93F535283028; **Taxon:** genus: Microvelia; specificEpithet: mimula; taxonRank: species; scientificNameAuthorship: White, 1879; **Location:** stateProvince: Paraná; municipality: Balsa Nova; locality: São Luiz do Purumã, Rio das Pombas; decimalLatitude: -25.44024; decimalLongitude: -49.74974; **Identification:** identificationID: Microvelia mimula; identifiedBy: C. F. B. Floriano; **Event:** samplingProtocol: "D" Net; year: 2017; month: IV; **Record Level:** institutionCode: CEIOC; basisOfRecord: PreservedSpecimen
- **Type status:** Other material. **Occurrence:** recordedBy: C. F. B. Floriano; individualCount: 10; sex: male; lifeStage: adult; occurrenceID: B52123D1-986C-5611-B136-3555F6090EC4; **Taxon:** genus: Microvelia; specificEpithet: mimula; taxonRank: species; scientificNameAuthorship: White, 1879; **Location:** stateProvince: Paraná; municipality: Tuneiras do Oeste; locality: Reserva Biológica das Perobas, Rio Mouro; decimalLatitude: -23.8850; decimalLongitude: -52.8072; **Identification:** identificationID: Microvelia mimula; identifiedBy: C. F. B. Floriano; **Event:** samplingProtocol: "D" Net; year: 2024; month: IV; **Record Level:** institutionCode: CEIOC; basisOfRecord: PreservedSpecimen
- **Type status:** Other material. **Occurrence:** recordedBy: C. F. B. Floriano; individualCount: 5; sex: female; lifeStage: adult; occurrenceID: 391972BF-0E8E-5736-9ABF-9B505FE952AC; **Taxon:** genus: Microvelia; specificEpithet: mimula; taxonRank: species; scientificNameAuthorship: White, 1879; **Location:** stateProvince: Paraná; municipality: Tuneiras do Oeste; locality: Reserva Biológica das Perobas, Rio Mouro; decimalLatitude: -23.8850; decimalLongitude: -52.8072; **Identification:** identificationID: Microvelia mimula; identifiedBy: C. F. B. Floriano; **Event:** samplingProtocol: "D" Net; year: 2024; month: IV; **Record Level:** institutionCode: CEIOC; basisOfRecord: PreservedSpecimen
- **Type status:** Other material. **Occurrence:** recordedBy: C. F. B. Floriano; individualCount: 3; sex: male; lifeStage: adult; occurrenceID: 1863DEE0-BC0C-55A9-82AE-18453D201232; **Taxon:** genus: Microvelia; specificEpithet: mimula; taxonRank: species; scientificNameAuthorship: White, 1879; **Location:** stateProvince: Paraná; municipality: Tuneiras do Oeste; locality: Reserva Biológica das Perobas, Lagoa; decimalLatitude: -23.8894; decimalLongitude: -52.8144; **Identification:** identificationID: Microvelia mimula; identifiedBy: C. F. B. Floriano; **Event:** samplingProtocol: "D" Net; year: 2024; month: IV; **Record Level:** institutionCode: CEIOC; basisOfRecord: PreservedSpecimen
- **Type status:** Other material. **Occurrence:** recordedBy: C. F. B. Floriano; individualCount: 5; sex: female; lifeStage: adult; occurrenceID: C071F6C7-8E20-5EB5-9406-D84653729DD3; **Taxon:** genus: Microvelia; specificEpithet: mimula; taxonRank: species; scientificNameAuthorship: White, 1879; **Location:** stateProvince: Paraná; municipality: Tuneiras do Oeste; locality: Reserva Biológica das Perobas, Lagoa; decimalLatitude: -23.8894; decimalLongitude: -52.8144; **Identification:** identificationID: Microvelia mimula; identifiedBy: C. F. B. Floriano; **Event:** samplingProtocol: "D" Net; year: 2024; month: IV; **Record Level:** institutionCode: CEIOC; basisOfRecord: PreservedSpecimen
- **Type status:** Other material. **Occurrence:** recordedBy: C. F. B. Floriano; individualCount: 2; sex: male; lifeStage: adult; occurrenceID: 87E292CF-9209-5822-BD34-4C659D5BCC46; **Taxon:** genus: Microvelia; specificEpithet: mimula; taxonRank: species; scientificNameAuthorship: White, 1879; **Location:** stateProvince: Paraná; municipality: Tuneiras do Oeste; locality: Reserva Biológica das Perobas, Rio Saquarema; decimalLatitude: -23.8725; decimalLongitude: -52.7864; **Identification:** identificationID: Microvelia mimula; identifiedBy: C. F. B. Floriano; **Event:** samplingProtocol: "D" Net; year: 2024; month: IV; **Record Level:** institutionCode: CEIOC; basisOfRecord: PreservedSpecimen
- **Type status:** Other material. **Occurrence:** recordedBy: C. F. B. Floriano; individualCount: 1; sex: male; lifeStage: adult; occurrenceID: DF0E4420-5872-5F69-AAD1-DCCC07CA497C; **Taxon:** genus: Microvelia; specificEpithet: mimula; taxonRank: species; scientificNameAuthorship: White, 1879; **Location:** stateProvince: Paraná; municipality: Tuneiras do Oeste; locality: Reserva Biológica das Perobas; decimalLatitude: -23.8726; decimalLongitude: -52.7863; **Identification:** identificationID: Microvelia mimula; identifiedBy: C. F. B. Floriano; **Event:** samplingProtocol: Pensilvânia; year: 2024; month: IV; **Record Level:** institutionCode: CEIOC; basisOfRecord: PreservedSpecimen
- **Type status:** Other material. **Occurrence:** recordedBy: C. F. B. Floriano; individualCount: 2; sex: female; lifeStage: adult; occurrenceID: 38ACDC25-C46D-5DA8-A0B4-0508258E165C; **Taxon:** genus: Microvelia; specificEpithet: mimula; taxonRank: species; scientificNameAuthorship: White, 1879; **Location:** stateProvince: Paraná; municipality: Tuneiras do Oeste; locality: Reserva Biológica das Perobas; decimalLatitude: -23.8726; decimalLongitude: -52.7863; **Identification:** identificationID: Microvelia mimula; identifiedBy: C. F. B. Floriano; **Event:** samplingProtocol: Pensilvânia; year: 2024; month: IV; **Record Level:** institutionCode: CEIOC; basisOfRecord: PreservedSpecimen
- **Type status:** Other material. **Occurrence:** recordedBy: C. F. B. Floriano; individualCount: 2; sex: male; lifeStage: adult; occurrenceID: 94043BC2-25EE-5B0A-A4B1-1DA5735970C6; **Taxon:** genus: Microvelia; specificEpithet: mimula; taxonRank: species; scientificNameAuthorship: White, 1879; **Location:** stateProvince: Paraná; municipality: Marialva; locality: Ribeirão Sarandi, Cachoeira Marialva; decimalLatitude: -23.4423; decimalLongitude: -51.7695; **Identification:** identificationID: Microvelia mimula; identifiedBy: C. F. B. Floriano; **Event:** samplingProtocol: "D" Net; year: 2024; month: III; **Record Level:** institutionCode: CEIOC; basisOfRecord: PreservedSpecimen
- **Type status:** Other material. **Occurrence:** recordedBy: C. F. B. Floriano; individualCount: 2; sex: female; lifeStage: adult; occurrenceID: E62737AF-1ED5-52F9-B46D-9F9547FF6326; **Taxon:** genus: Microvelia; specificEpithet: mimula; taxonRank: species; scientificNameAuthorship: White, 1879; **Location:** stateProvince: Paraná; municipality: Marialva; locality: Ribeirão Sarandi, Cachoeira Marialva; decimalLatitude: -23.4423; decimalLongitude: -51.7695; **Identification:** identificationID: Microvelia mimula; identifiedBy: C. F. B. Floriano; **Event:** samplingProtocol: "D" Net; year: 2024; month: III; **Record Level:** institutionCode: CEIOC; basisOfRecord: PreservedSpecimen

##### Distribution

Cuba, Puerto Rico, Costa Rica, Panama, St. Vicent & the Grenadines, Barbados, Grenada, Trinidad & Tobago, Colombia, Venezuela, Suriname, French Guiana, Brazil, Ecuador, Peru, Paraguay, Argentina and Uruguay.

##### Notes

First records from Paraná (Fig. 3I, Fig. 5A). Previously reported from Santa Catarina and Rio Grande do Sul (Drake and Hussey 1951, Rodrigues et al. 2021).

#### Microvelia minima

Drake, 1952

BD3BC7F4-EB87-5BEE-90C7-43D055D09170

##### Materials

- **Type status:** Other material. **Occurrence:** recordedBy: C. F. B. Floriano & T. Polizei; individualCount: 1; sex: female; lifeStage: adult; occurrenceID: 69D1539F-E34A-5F2B-ABA0-AF8AE557C230; **Taxon:** genus: Microvelia; specificEpithet: minima; taxonRank: species; scientificNameAuthorship: Drake, 1952; **Location:** stateProvince: Rio Grande do Sul; municipality: Cambará do Sul; locality: Parque Nacional de Aparados da Serra, Córrego Preá; decimalLatitude: -29.17; decimalLongitude: -50.09; **Identification:** identificationID: Microvelia minima; identifiedBy: C. F. B. Floriano; **Event:** samplingProtocol: "D" Net; year: 2016; month: IX; **Record Level:** institutionCode: CEIOC; basisOfRecord: PreservedSpecimen
- **Type status:** Other material. **Occurrence:** recordedBy: C. F. B. Floriano & T. Polizei; individualCount: 2; sex: male; lifeStage: adult; occurrenceID: 05CF866F-EA5F-5064-A71F-D38176F9FEA0; **Taxon:** genus: Microvelia; specificEpithet: minima; taxonRank: species; scientificNameAuthorship: Drake, 1952; **Location:** stateProvince: Rio Grande do Sul; municipality: Cambará do Sul; locality: Parque Nacional da Aparados da Serra, Córrego Marcel; decimalLatitude: -29.17; decimalLongitude: -50.09; **Identification:** identificationID: Microvelia minima; identifiedBy: C. F. B. Floriano; **Event:** samplingProtocol: "D" Net; year: 2016; month: IX; **Record Level:** institutionCode: CEIOC; basisOfRecord: PreservedSpecimen
- **Type status:** Other material. **Occurrence:** recordedBy: C. F. B. Floriano & T. Polizei; individualCount: 4; sex: female; lifeStage: adult; occurrenceID: 3EA65E23-8900-5764-8921-0A592B7C8206; **Taxon:** genus: Microvelia; specificEpithet: minima; taxonRank: species; scientificNameAuthorship: Drake, 1952; **Location:** stateProvince: Rio Grande do Sul; municipality: Cambará do Sul; locality: Parque Nacional da Aparados da Serra, Córrego Marcel; decimalLatitude: -29.17; decimalLongitude: -50.09; **Identification:** identificationID: Microvelia minima; identifiedBy: C. F. B. Floriano; **Event:** samplingProtocol: "D" Net; year: 2016; month: IX; **Record Level:** institutionCode: CEIOC; basisOfRecord: PreservedSpecimen
- **Type status:** Other material. **Occurrence:** recordedBy: F. Plaumann; individualCount: 4; sex: male; lifeStage: adult; occurrenceID: 9F266C00-67C4-51CD-8CB4-6AA0FE64DC0E; **Taxon:** genus: Microvelia; specificEpithet: minima; taxonRank: species; scientificNameAuthorship: Drake, 1952; **Location:** stateProvince: Rio Grande do Sul; municipality: Santa Cruz do Sul; locality: Bach (brook); verbatimElevation: 200 m; decimalLatitude: -29.71; decimalLongitude: -52.43; **Identification:** identificationID: Microvelia minima; identifiedBy: C. F. B. Floriano; **Event:** year: 1959; month: XI; **Record Level:** institutionCode: MZUSP; basisOfRecord: PreservedSpecimen
- **Type status:** Other material. **Occurrence:** recordedBy: F. Plaumann; individualCount: 6; sex: female; lifeStage: adult; occurrenceID: A96F8B9F-1CC3-521D-85A3-748B12CCFCE8; **Taxon:** genus: Microvelia; specificEpithet: minima; taxonRank: species; scientificNameAuthorship: Drake, 1952; **Location:** stateProvince: Rio Grande do Sul; municipality: Santa Cruz do Sul; locality: Bach (brook); decimalLatitude: -29.71; decimalLongitude: -52.43; **Identification:** identificationID: Microvelia minima; identifiedBy: C. F. B. Floriano; **Event:** year: 1959; month: XI; **Record Level:** institutionCode: MZUSP; basisOfRecord: PreservedSpecimen
- **Type status:** Other material. **Occurrence:** recordedBy: F. Plaumann; individualCount: 4; sex: male; lifeStage: adult; occurrenceID: A02F7BEC-0623-5101-AC32-127DC56BD3AD; **Taxon:** genus: Microvelia; specificEpithet: minima; taxonRank: species; scientificNameAuthorship: Drake, 1952; **Location:** stateProvince: Rio Grande do Sul; municipality: Caçapava do Sul; locality: Quelle (Spring); decimalLatitude: -30.50; decimalLongitude: -53.50; **Identification:** identificationID: Microvelia minima; identifiedBy: C. F. B. Floriano; **Event:** year: 1959; month: IX; **Record Level:** institutionCode: MZUSP; basisOfRecord: PreservedSpecimen
- **Type status:** Other material. **Occurrence:** recordedBy: F. Plaumann; individualCount: 5; sex: female; lifeStage: adult; occurrenceID: DDED9C15-7CA7-5D1B-9BFC-1EFCF2D63F74; **Taxon:** genus: Microvelia; specificEpithet: minima; taxonRank: species; scientificNameAuthorship: Drake, 1952; **Location:** stateProvince: Rio Grande do Sul; municipality: Caçapava do Sul; locality: Quelle (Spring); decimalLatitude: -30.50; decimalLongitude: -53.50; **Identification:** identificationID: Microvelia minima; identifiedBy: C. F. B. Floriano; **Event:** year: 1959; month: IX; **Record Level:** institutionCode: MZUSP; basisOfRecord: PreservedSpecimen
- **Type status:** Other material. **Occurrence:** recordedBy: C. F. B. Floriano & T. Malacarne; individualCount: 2; sex: female; lifeStage: adult; occurrenceID: 4D73FFF1-D2DB-5509-B2D4-BFC7DDC0CD7A; **Taxon:** genus: Microvelia; specificEpithet: minima; taxonRank: species; scientificNameAuthorship: Drake, 1952; **Location:** stateProvince: Santa Catarina; municipality: Indaial; locality: Parque Nacional da Serra do Itajaí, Pesque Pague do Wili, Riacho pequeno; decimalLatitude: -27.015; decimalLongitude: -49.164; **Identification:** identificationID: Microvelia minima; identifiedBy: C. F. B. Floriano; **Event:** samplingProtocol: "D" Net; year: 2017; month: IV; **Record Level:** institutionCode: CEIOC; basisOfRecord: PreservedSpecimen
- **Type status:** Other material. **Occurrence:** recordedBy: C. F. B. Floriano; individualCount: 1; sex: male; lifeStage: adult; occurrenceID: 1DAEFB7D-D74A-5681-8126-FB858435E367; **Taxon:** genus: Microvelia; specificEpithet: minima; taxonRank: species; scientificNameAuthorship: Drake, 1952; **Location:** stateProvince: Paraná; municipality: Tuneiras do Oeste; locality: Reserva Biológica das Perobas, Rio Saquarema; decimalLatitude: -23.8725; decimalLongitude: -52.7864; **Identification:** identificationID: Microvelia minima; identifiedBy: C. F. B. Floriano; **Event:** samplingProtocol: "D" Net; year: 2024; month: IV; **Record Level:** institutionCode: CEIOC; basisOfRecord: PreservedSpecimen
- **Type status:** Other material. **Occurrence:** recordedBy: C. F. B. Floriano; individualCount: 1; sex: female; lifeStage: adult; occurrenceID: 22F67462-8D1D-503A-B0FA-16A006030A45; **Taxon:** genus: Microvelia; specificEpithet: minima; taxonRank: species; scientificNameAuthorship: Drake, 1952; **Location:** stateProvince: Paraná; municipality: Tuneiras do Oeste; locality: Reserva Biológica das Perobas, Rio Saquarema; decimalLatitude: -23.8725; decimalLongitude: -52.7864; **Identification:** identificationID: Microvelia minima; identifiedBy: C. F. B. Floriano; **Event:** samplingProtocol: "D" Net; year: 2024; month: IV; **Record Level:** institutionCode: CEIOC; basisOfRecord: PreservedSpecimen

##### Distribution

Brazil.

##### Notes

**Figure 6.**
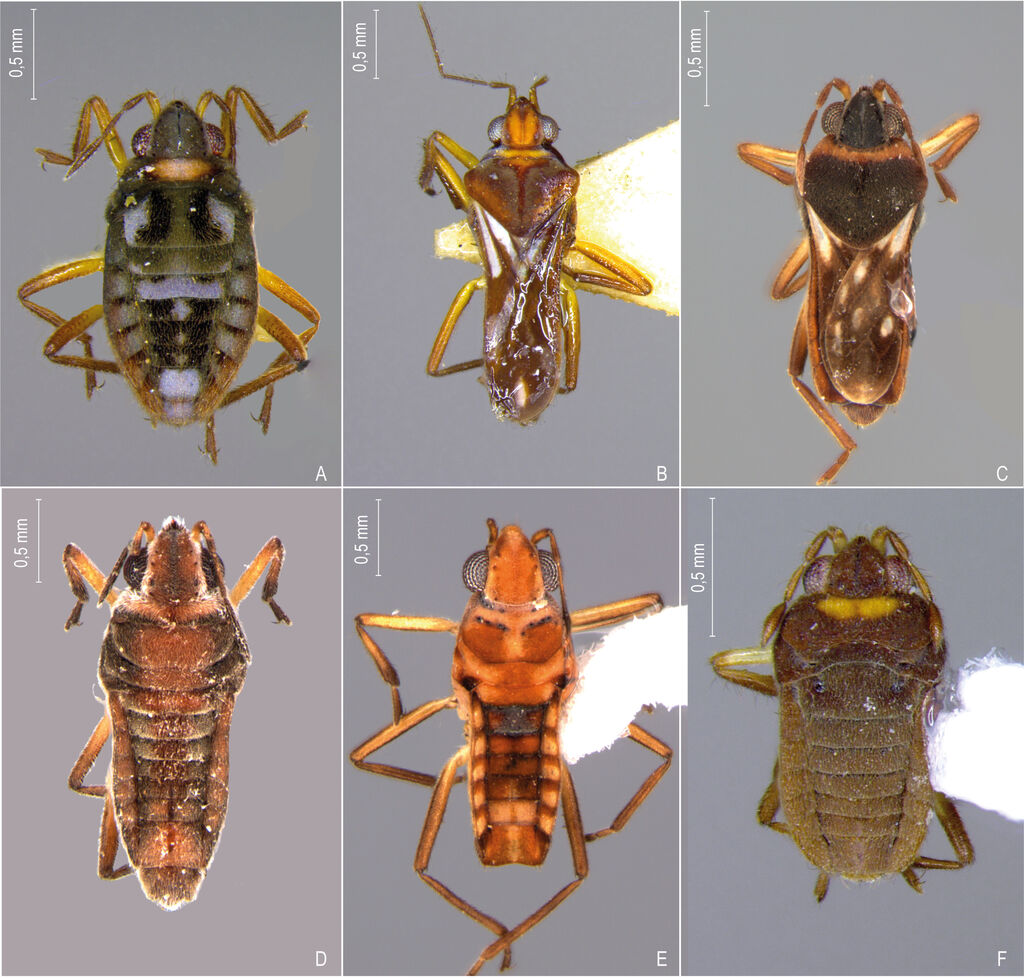
Gerridae from the South Region of Brazil, male, habitus, dorsal view. **A** *Microvelia minima*; **B** *Microvelia novana*; **C** *Microvelia picinguaba*; **D** *Microvelia pulchella*; **E** *Microvelia sousorum*; **F** *Microvelia venustatis*.

First records from Paraná and Rio Grande do Sul (Fig. 5A, Fig. 6A). Described from the States of Rio de Janeiro and Santa Catarina (Drake 1952a).

#### Microvelia novana

Drake & Plaumann, 1955

F9EC8D2D-C3B3-51DF-95D8-9C9E2654EABD

##### Materials

- **Type status:** Holotype. **Occurrence:** recordedBy: F. Plaumann; individualCount: 1; sex: male; lifeStage: adult; occurrenceID: 8BB9E088-B28C-530A-A2B7-E35092B5EE0F; **Taxon:** genus: Microvelia; specificEpithet: novana; taxonRank: species; scientificNameAuthorship: Drake & Plaumann, 1955; **Location:** stateProvince: Santa Catarina; municipality: Seara; locality: Nova Teutônia; decimalLatitude: -27.19; decimalLongitude: -51.22; **Identification:** identificationID: Microvelia novana; identifiedBy: C. Drake; **Event:** year: 1953; month: VIII; **Record Level:** institutionCode: NMNH; basisOfRecord: PreservedSpecimen
- **Type status:** Paratype. **Occurrence:** recordedBy: F. Plaumann; individualCount: 11; sex: male; lifeStage: adult; occurrenceID: FB40BEDA-369A-5908-993D-3C43FDFC9F09; **Taxon:** genus: Microvelia; specificEpithet: novana; taxonRank: species; scientificNameAuthorship: Drake & Plaumann, 1955; **Location:** stateProvince: Santa Catarina; municipality: Seara; locality: Nova Teutônia; decimalLatitude: -27.19; decimalLongitude: -52.33; **Identification:** identificationID: Microvelia novana; identifiedBy: C. Drake; **Event:** year: 1953; month: VIII; **Record Level:** institutionCode: NMNH; basisOfRecord: PreservedSpecimen
- **Type status:** Paratype. **Occurrence:** recordedBy: F. Plaumann; individualCount: 8; sex: female; lifeStage: adult; occurrenceID: FB95CEE1-C736-542B-8792-931321E285B4; **Taxon:** genus: Microvelia; specificEpithet: novana; taxonRank: species; scientificNameAuthorship: Drake & Plaumann, 1955; **Location:** stateProvince: Santa Catarina; municipality: Seara; locality: Nova Teutônia; decimalLatitude: -27.19; decimalLongitude: -52.33; **Identification:** identificationID: Microvelia novana; identifiedBy: C. Drake; **Event:** year: 1953; month: VIII; **Record Level:** institutionCode: NMNH; basisOfRecord: PreservedSpecimen
- **Type status:** Other material. **Occurrence:** recordedBy: C. F. B. Floriano; individualCount: 1; sex: male; lifeStage: adult; occurrenceID: BC7B0EF3-D519-5C7A-A6EF-6E90B08903C2; **Taxon:** genus: Microvelia; specificEpithet: novana; taxonRank: species; scientificNameAuthorship: Drake & Plaumann, 1955; **Location:** stateProvince: Paraná; municipality: Faxinal; locality: Cachoeira do Rio São Pedro; decimalLatitude: -23.9756; decimalLongitude: -51.2247; **Identification:** identificationID: Microvelia novana; identifiedBy: C. F. B. Floriano; **Event:** samplingProtocol: "D" Net; year: 2020; month: IX; **Record Level:** institutionCode: CEIOC; basisOfRecord: PreservedSpecimen

##### Distribution

Brazil.

##### Notes

First record from Paraná (Fig. 5A, Fig. 6B). Described from Santa Catarina (Drake and Plaumann 1955).

#### Microvelia picinguaba

Moreira & Barbosa, 2011

20642D81-5458-5BE2-9B2D-394C94E63956

##### Materials

- **Type status:** Other material. **Occurrence:** recordedBy: C. F. B. Floriano & T. Malacarne; individualCount: 1; sex: male; lifeStage: adult; occurrenceID: 37231308-198E-5259-B81A-48F251852ADF; **Taxon:** genus: Microvelia; specificEpithet: picinguaba; taxonRank: species; scientificNameAuthorship: Moreira & Barbosa, 2011; **Location:** stateProvince: Santa Catarina; municipality: Blumenau; locality: Parque Nacional da Serra do Itajaí, Parque Natural Municipal Nascentes do Garcia, lagoa trilha da curva do rio; decimalLatitude: -27.0610; decimalLongitude: -49.0951; **Identification:** identificationID: Microvelia picinguaba; identifiedBy: C. F. B. Floriano; **Event:** samplingProtocol: "D" Net; year: 2017; month: IV; **Record Level:** institutionCode: CEIOC; basisOfRecord: PreservedSpecimen
- **Type status:** Other material. **Occurrence:** recordedBy: C. F. B. Floriano & T. Malacarne; individualCount: 1; sex: female; lifeStage: adult; occurrenceID: 04AB91F4-A1A4-5949-A41C-3BA66B744A5C; **Taxon:** genus: Microvelia; specificEpithet: picinguaba; taxonRank: species; scientificNameAuthorship: Moreira & Barbosa, 2011; **Location:** stateProvince: Santa Catarina; municipality: Blumenau; locality: Parque Nacional da Serra do Itajaí, Parque Natural Municipal Nascentes do Garcia, lagoa trilha da curva do rio; decimalLatitude: -27.0610; decimalLongitude: -49.0951; **Identification:** identificationID: Microvelia picinguaba; identifiedBy: C. F. B. Floriano; **Event:** samplingProtocol: "D" Net; year: 2017; month: IV; **Record Level:** institutionCode: CEIOC; basisOfRecord: PreservedSpecimen
- **Type status:** Other material. **Occurrence:** recordedBy: C. F. B. Floriano & T. Malacarne; individualCount: 1; sex: male; lifeStage: adult; occurrenceID: 307C77A8-2B52-56A2-B857-E5BDB73A1A40; **Taxon:** genus: Microvelia; specificEpithet: picinguaba; taxonRank: species; scientificNameAuthorship: Moreira & Barbosa, 2011; **Location:** stateProvince: Santa Catarina; municipality: Blumenau; locality: Parque Nacional da Serra do Itajaí, Parque Natural Municipal Nascentes do Garcia, lagoa negra; decimalLatitude: -27.0532; decimalLongitude: -49.0878; **Identification:** identificationID: Microvelia picinguaba; identifiedBy: C. F. B. Floriano; **Event:** samplingProtocol: "D" Net; year: 2017; month: IV; **Record Level:** institutionCode: CEIOC; basisOfRecord: PreservedSpecimen

##### Distribution

Brazil.

##### Notes

First records from Santa Catarina and the South Region of Brazil (Fig. 5B, Fig. 6C).

#### Microvelia pulchella

Westwood, 1834

762C64A9-10A7-5CEE-A4E0-8A9A052E58B7

##### Materials

- **Type status:** Other material. **Occurrence:** recordedBy: C. F. B. Floriano & T. Malacarne; individualCount: 1; sex: male; lifeStage: adult; occurrenceID: 441BF355-4553-5A4B-B230-DFC1ACA870D3; **Taxon:** genus: Microvelia; specificEpithet: pulchella; taxonRank: species; scientificNameAuthorship: Westwood, 1834; **Location:** stateProvince: Santa Catarina; municipality: Blumenau; locality: Parque Nacional da Serra do Itajaí, Parque Natural Municipal Nascentes do Garcia, lagoa negra; decimalLatitude: -27.0532; decimalLongitude: -49.0878; **Identification:** identificationID: Microvelia pulchella; identifiedBy: C. F. B. Floriano; **Event:** samplingProtocol: "D" Net; year: 2017; month: IV; **Record Level:** institutionCode: CEIOC; basisOfRecord: PreservedSpecimen
- **Type status:** Other material. **Occurrence:** recordedBy: C. F. B. Floriano & T. Malacarne; individualCount: 1; sex: female; lifeStage: adult; occurrenceID: B5E57FB5-552B-5C13-8B20-0BE0EF035A07; **Taxon:** genus: Microvelia; specificEpithet: pulchella; taxonRank: species; scientificNameAuthorship: Westwood, 1834; **Location:** stateProvince: Santa Catarina; municipality: Blumenau; locality: Parque Nacional da Serra do Itajaí, Parque Natural Municipal Nascentes do Garcia, lagoa negra; decimalLatitude: -27.0532; decimalLongitude: -49.0878; **Identification:** identificationID: Microvelia pulchella; identifiedBy: C. F. B. Floriano; **Event:** samplingProtocol: "D" Net; year: 2017; month: IV; **Record Level:** institutionCode: CEIOC; basisOfRecord: PreservedSpecimen
- **Type status:** Other material. **Occurrence:** recordedBy: F. Plaumman; individualCount: 1; sex: female; lifeStage: adult; occurrenceID: 33D2AF34-B2A6-58B7-95EE-3D233407FC84; **Taxon:** genus: Microvelia; specificEpithet: pulchella; taxonRank: species; scientificNameAuthorship: Westwood, 1834; **Location:** stateProvince: Santa Catarina; municipality: Chapecó; locality: Linha facão; decimalLatitude: -27.08; decimalLongitude: -52.62; **Identification:** identificationID: Microvelia pulchella; identifiedBy: C. F. B. Floriano; **Event:** year: 1957; month: V; **Record Level:** institutionCode: MZUSP; basisOfRecord: PreservedSpecimen
- **Type status:** Other material. **Occurrence:** recordedBy: F. Plaumman; individualCount: 2; sex: male; lifeStage: adult; occurrenceID: AEE47435-F38E-55B0-91D1-4895DCB56811; **Taxon:** genus: Microvelia; specificEpithet: pulchella; taxonRank: species; scientificNameAuthorship: Westwood, 1834; **Location:** stateProvince: Rio Grande do Sul; municipality: Maquiné; locality: Lagoa Quadros; decimalLatitude: -29.75; decimalLongitude: -50.11; **Identification:** identificationID: Microvelia pulchella; identifiedBy: C. F. B. Floriano; **Event:** year: 1959; month: IV; **Record Level:** institutionCode: MZUSP; basisOfRecord: PreservedSpecimen
- **Type status:** Other material. **Occurrence:** recordedBy: F. Plaumman; individualCount: 2; sex: female; lifeStage: adult; occurrenceID: 7E81B6E2-77DC-535A-8BFF-517AC82F2C58; **Taxon:** genus: Microvelia; specificEpithet: pulchella; taxonRank: species; scientificNameAuthorship: Westwood, 1834; **Location:** stateProvince: Rio Grande do Sul; municipality: Maquiné; locality: Lagoa Quadros; decimalLatitude: -29.75; decimalLongitude: -50.11; **Identification:** identificationID: Microvelia pulchella; identifiedBy: C. F. B. Floriano; **Event:** year: 1959; month: IV; **Record Level:** institutionCode: MZUSP; basisOfRecord: PreservedSpecimen
- **Type status:** Other material. **Occurrence:** recordedBy: F. Plaumman; individualCount: 1; sex: male; lifeStage: adult; occurrenceID: 5D0A0687-9BDA-51EB-BB6C-87675D45CB34; **Taxon:** genus: Microvelia; specificEpithet: pulchella; taxonRank: species; scientificNameAuthorship: Westwood, 1834; **Location:** stateProvince: Rio Grande do Sul; municipality: Torres; locality: Lagoa Itapeva; decimalLatitude: -29.38; decimalLongitude: -49.80; **Identification:** identificationID: Microvelia pulchella; identifiedBy: C. F. B. Floriano; **Event:** year: 1959; month: IV; **Record Level:** institutionCode: MZUSP; basisOfRecord: PreservedSpecimen
- **Type status:** Other material. **Occurrence:** recordedBy: J. R. I. Ribeiro; individualCount: 1; sex: male; lifeStage: adult; occurrenceID: D89B64FE-9031-5687-A105-BC98CF26B4AE; **Taxon:** genus: Microvelia; specificEpithet: pulchella; taxonRank: species; scientificNameAuthorship: Westwood, 1834; **Location:** stateProvince: Rio Grande do Sul; municipality: Tapes; decimalLatitude: -30.70; decimalLongitude: -51.56; **Identification:** identificationID: Microvelia pulchella; identifiedBy: C. F. B. Floriano; **Event:** year: 2002; **Record Level:** institutionCode: CEIOC; basisOfRecord: PreservedSpecimen
- **Type status:** Other material. **Occurrence:** recordedBy: C. F. B. Floriano; individualCount: 8; sex: male; lifeStage: adult; occurrenceID: 04D7808E-769F-519C-9006-14002CAAFB92; **Taxon:** genus: Microvelia; specificEpithet: pulchella; taxonRank: species; scientificNameAuthorship: Westwood, 1834; **Location:** stateProvince: Paraná; municipality: Tuneiras do Oeste; locality: Reserva Biológica das Perobas, Lagoa; decimalLatitude: -23.8894; decimalLongitude: -52.8144; **Identification:** identificationID: Microvelia pulchella; identifiedBy: C. F. B. Floriano; **Event:** samplingProtocol: "D" Net; year: 2024; month: IV; **Record Level:** institutionCode: CEIOC; basisOfRecord: PreservedSpecimen
- **Type status:** Other material. **Occurrence:** recordedBy: C. F. B. Floriano; individualCount: 4; sex: female; lifeStage: adult; occurrenceID: FBF8ECCF-A91C-5547-B6B5-4EDE76F722E8; **Taxon:** genus: Microvelia; specificEpithet: pulchella; taxonRank: species; scientificNameAuthorship: Westwood, 1834; **Location:** stateProvince: Paraná; municipality: Tuneiras do Oeste; locality: Reserva Biológica das Perobas, Lagoa; decimalLatitude: -23.8894; decimalLongitude: -52.8144; **Identification:** identificationID: Microvelia pulchella; identifiedBy: C. F. B. Floriano; **Event:** samplingProtocol: "D" Net; year: 2024; month: IV; **Record Level:** institutionCode: CEIOC; basisOfRecord: PreservedSpecimen
- **Type status:** Other material. **Occurrence:** recordedBy: C. F. B. Floriano; individualCount: 4; sex: male; lifeStage: adult; occurrenceID: 628933DF-263A-5B18-88DB-F5E2806D1D94; **Taxon:** genus: Microvelia; specificEpithet: pulchella; taxonRank: species; scientificNameAuthorship: Westwood, 1834; **Location:** stateProvince: Paraná; municipality: Tuneiras do Oeste; locality: Reserva Biológica das Perobas, poça na estrada; decimalLatitude: -23.8553; decimalLongitude: -52.8114; **Identification:** identificationID: Microvelia pulchella; identifiedBy: C. F. B. Floriano; **Event:** samplingProtocol: "D" Net; year: 2024; month: IV; **Record Level:** institutionCode: CEIOC; basisOfRecord: PreservedSpecimen
- **Type status:** Other material. **Occurrence:** recordedBy: C. F. B. Floriano; individualCount: 7; sex: female; lifeStage: adult; occurrenceID: 3D83620F-169A-552B-A464-0842E27594A4; **Taxon:** genus: Microvelia; specificEpithet: pulchella; taxonRank: species; scientificNameAuthorship: Westwood, 1834; **Location:** stateProvince: Paraná; municipality: Tuneiras do Oeste; locality: Reserva Biológica das Perobas, poça na estrada; decimalLatitude: -23.8553; decimalLongitude: -52.8114; **Identification:** identificationID: Microvelia pulchella; identifiedBy: C. F. B. Floriano; **Event:** samplingProtocol: "D" Net; year: 2024; month: IV; **Record Level:** institutionCode: CEIOC; basisOfRecord: PreservedSpecimen

##### Distribution

Canada, United States, Mexico, Guatemala, Bahamas, Cuba, Jamaica, Dominican Republic, Puerto Rico, U. S. Virgin Islands, Cayman Islands, Anguilla, St. Martin, Saba, St. Kitts & Nevis, Guadeloupe, Martinique, Costa Rica, Panama, Aruba, St. Vicent & the Grenadines, Barbados, Curaçao, Klein Curaçao, Bonaire, Klein Bonaire, Grenada, Trinidad & Tobago, Colombia, Venezuela, French Guiana, Brazil, Ecuador, Peru and Argentina.

##### Notes

First records from Paraná (Fig. 5B, Fig. 6D). Previously reported from Santa Catarina and Rio Grande do Sul (Drake and Plaumann 1955, Rodrigues et al. 2021).

#### Microvelia sousorum

dos Santos, Rodrigues, Couceiro & Moreira, 2021

6F2FF8E2-EDED-584B-B2F6-C87EFC282A80

##### Materials

- **Type status:** Other material. **Occurrence:** recordedBy: C. F. B. Floriano; individualCount: 1; sex: male; lifeStage: adult; occurrenceID: 970D51D4-8623-5B38-BB99-FA5488213B9C; **Taxon:** genus: Microvelia; specificEpithet: sousorum; taxonRank: species; scientificNameAuthorship: dos Santos, Rodrigues, Couceiro & Moreira, 2021; **Location:** stateProvince: Paraná; municipality: Tuneiras do Oeste; locality: Reserva Biológica das Perobas; decimalLatitude: -23.8797; decimalLongitude: -52.8194; **Identification:** identificationID: Microvelia sousorum; identifiedBy: C. F. B. Floriano; **Event:** samplingProtocol: "D" Net; year: 2024; month: IV; **Record Level:** institutionCode: CEIOC; basisOfRecord: PreservedSpecimen
- **Type status:** Other material. **Occurrence:** recordedBy: C. F. B. Floriano; individualCount: 2; sex: male; lifeStage: adult; occurrenceID: E744F9B3-41BD-54B5-BE68-A3C7017DE9C1; **Taxon:** genus: Microvelia; specificEpithet: sousorum; taxonRank: species; scientificNameAuthorship: dos Santos, Rodrigues, Couceiro & Moreira, 2021; **Location:** stateProvince: Paraná; municipality: Tuneiras do Oeste; locality: Reserva Biológica das Perobas, Rio Saquarema; decimalLatitude: -23.8725; decimalLongitude: -52.7864; **Identification:** identificationID: Microvelia sousorum; identifiedBy: C. F. B. Floriano; **Event:** samplingProtocol: "D" Net; year: 2024; month: IV; **Record Level:** institutionCode: CEIOC; basisOfRecord: PreservedSpecimen

##### Distribution

Brazil.

##### Notes

First records from Paraná and the South Region of Brazil (Fig. 5B, Fig. 6E).

#### Microvelia venustatis

Drake & Harris, 1933

E897B279-C177-53F9-86CC-3D59ADA4864B

##### Materials

- **Type status:** Other material. **Occurrence:** recordedBy: C. F. B. Floriano & T. Malacarne; individualCount: 39; sex: male; lifeStage: adult; occurrenceID: 073EEB49-69F1-559F-B4EC-6F3AC08FA281; **Taxon:** genus: Microvelia; specificEpithet: venustatis; taxonRank: species; scientificNameAuthorship: Drake & Harris, 1933; **Location:** stateProvince: Santa Catarina; municipality: Blumenau; locality: Parque Nacional da Serra do Itajaí, Parque Natural Municipal Nascentes do Garcia, lagoa trilha da curva do rio; decimalLatitude: -27.0610; decimalLongitude: -49.0951; **Identification:** identificationID: Microvelia venustatis; identifiedBy: C. F. B. Floriano; **Event:** samplingProtocol: "D" Net; year: 2017; month: IV; **Record Level:** institutionCode: CEIOC; basisOfRecord: PreservedSpecimen
- **Type status:** Other material. **Occurrence:** recordedBy: C. F. B. Floriano & T. Malacarne; individualCount: 6; sex: female; lifeStage: adult; occurrenceID: 30441B50-6103-5E8E-8CD4-70F5B1B5257D; **Taxon:** genus: Microvelia; specificEpithet: venustatis; taxonRank: species; scientificNameAuthorship: Drake & Harris, 1933; **Location:** stateProvince: Santa Catarina; municipality: Blumenau; locality: Parque Nacional da Serra do Itajaí, Parque Natural Municipal Nascentes do Garcia, lagoa trilha da curva do rio; decimalLatitude: -27.0610; decimalLongitude: -49.0951; **Identification:** identificationID: Microvelia venustatis; identifiedBy: C. F. B. Floriano; **Event:** samplingProtocol: "D" Net; year: 2017; month: IV; **Record Level:** institutionCode: CEIOC; basisOfRecord: PreservedSpecimen
- **Type status:** Other material. **Occurrence:** recordedBy: C. F. B. Floriano & T. Malacarne; individualCount: 14; sex: male; lifeStage: adult; occurrenceID: D8075EBC-04A0-5191-B06F-3675E30D5E09; **Taxon:** genus: Microvelia; specificEpithet: venustatis; taxonRank: species; scientificNameAuthorship: Drake & Harris, 1933; **Location:** stateProvince: Santa Catarina; municipality: Blumenau; locality: Parque Nacional da Serra do Itajaí, Parque Natural Municipal Nascentes do Garcia, riacho pequeno; decimalLatitude: -27.052; decimalLongitude: -49.088; **Identification:** identificationID: Microvelia venustatis; identifiedBy: C. F. B. Floriano; **Event:** samplingProtocol: "D" Net; year: 2017; month: IV; **Record Level:** institutionCode: CEIOC; basisOfRecord: PreservedSpecimen
- **Type status:** Other material. **Occurrence:** recordedBy: C. F. B. Floriano & T. Malacarne; individualCount: 6; sex: female; lifeStage: adult; occurrenceID: 029C2586-D67D-56A3-BC97-6BA4555ECC9A; **Taxon:** genus: Microvelia; specificEpithet: venustatis; taxonRank: species; scientificNameAuthorship: Drake & Harris, 1933; **Location:** stateProvince: Santa Catarina; municipality: Blumenau; locality: Parque Nacional da Serra do Itajaí, Parque Natural Municipal Nascentes do Garcia, riacho pequeno; decimalLatitude: -27.052; decimalLongitude: -49.088; **Identification:** identificationID: Microvelia venustatis; identifiedBy: C. F. B. Floriano; **Event:** samplingProtocol: "D" Net; year: 2017; month: IV; **Record Level:** institutionCode: CEIOC; basisOfRecord: PreservedSpecimen
- **Type status:** Other material. **Occurrence:** recordedBy: C. F. B. Floriano & T. Polizei; individualCount: 8; sex: male; lifeStage: adult; occurrenceID: 5E112B3D-4DC7-5D0A-8B6A-862C74DF3CBC; **Taxon:** genus: Microvelia; specificEpithet: venustatis; taxonRank: species; scientificNameAuthorship: Drake & Harris, 1933; **Location:** stateProvince: Santa Catarina; municipality: Praia Grande; locality: Parque Nacional de Aparados da Serra, Rio do Boi; decimalLatitude: -29.213; decimalLongitude: -50.005; **Identification:** identificationID: Microvelia venustatis; identifiedBy: C. F. B. Floriano; **Event:** samplingProtocol: "D" Net; year: 2016; month: IX; **Record Level:** institutionCode: CEIOC; basisOfRecord: PreservedSpecimen
- **Type status:** Other material. **Occurrence:** recordedBy: C. F. B. Floriano & T. Polizei; individualCount: 8; sex: female; lifeStage: adult; occurrenceID: 333EB7C6-A451-5B4C-8DDC-3C85272E509D; **Taxon:** genus: Microvelia; specificEpithet: venustatis; taxonRank: species; scientificNameAuthorship: Drake & Harris, 1933; **Location:** stateProvince: Santa Catarina; municipality: Praia Grande; locality: Parque Nacional de Aparados da Serra, Rio do Boi; decimalLatitude: -29.213; decimalLongitude: -50.005; **Identification:** identificationID: Microvelia venustatis; identifiedBy: C. F. B. Floriano; **Event:** samplingProtocol: "D" Net; year: 2016; month: IX; **Record Level:** institutionCode: CEIOC; basisOfRecord: PreservedSpecimen
- **Type status:** Other material. **Occurrence:** recordedBy: C. F. B. Floriano & T. Polizei; individualCount: 16; sex: male; lifeStage: adult; occurrenceID: 6AE1AB8E-AB94-5436-B4DD-D5A51F4D3B2D; **Taxon:** genus: Microvelia; specificEpithet: venustatis; taxonRank: species; scientificNameAuthorship: Drake & Harris, 1933; **Location:** stateProvince: Santa Catarina; municipality: São Bonifácio; locality: Córrego Broemer; decimalLatitude: -28.01; decimalLongitude: -49.02; **Identification:** identificationID: Microvelia venustatis; identifiedBy: C. F. B. Floriano; **Event:** samplingProtocol: "D" Net; year: 2016; month: IX; **Record Level:** institutionCode: CEIOC; basisOfRecord: PreservedSpecimen
- **Type status:** Other material. **Occurrence:** recordedBy: C. F. B. Floriano & T. Polizei; individualCount: 2; sex: female; lifeStage: adult; occurrenceID: 5999D80A-67C4-5E0E-BEBB-B777BBC80AA3; **Taxon:** genus: Microvelia; specificEpithet: venustatis; taxonRank: species; scientificNameAuthorship: Drake & Harris, 1933; **Location:** stateProvince: Santa Catarina; municipality: São Bonifácio; locality: Córrego Broemer; decimalLatitude: -28.01; decimalLongitude: -49.02; **Identification:** identificationID: Microvelia venustatis; identifiedBy: C. F. B. Floriano; **Event:** samplingProtocol: "D" Net; year: 2016; month: IX; **Record Level:** institutionCode: CEIOC; basisOfRecord: PreservedSpecimen
- **Type status:** Other material. **Occurrence:** recordedBy: C. F. B. Floriano & T. Polizei; individualCount: 4; sex: male; lifeStage: adult; occurrenceID: 8BF71B0F-FB17-5286-BE7E-CBC3E4282211; **Taxon:** genus: Microvelia; specificEpithet: venustatis; taxonRank: species; scientificNameAuthorship: Drake & Harris, 1933; **Location:** stateProvince: Santa Catarina; municipality: Santo Amaro da Imperatriz; locality: Rio do Braço; decimalLatitude: -27.72; decimalLongitude: -48.72; **Identification:** identificationID: Microvelia venustatis; identifiedBy: C. F. B. Floriano; **Event:** samplingProtocol: "D" Net; year: 2016; month: IX; **Record Level:** institutionCode: CEIOC; basisOfRecord: PreservedSpecimen
- **Type status:** Other material. **Occurrence:** recordedBy: C. F. B. Floriano & T. Polizei; individualCount: 7; sex: female; lifeStage: adult; occurrenceID: C3021690-11C1-5469-90B2-0D943E7B3FB5; **Taxon:** genus: Microvelia; specificEpithet: venustatis; taxonRank: species; scientificNameAuthorship: Drake & Harris, 1933; **Location:** stateProvince: Santa Catarina; municipality: Santo Amaro da Imperatriz; locality: Rio do Braço; decimalLatitude: -27.72; decimalLongitude: -48.72; **Identification:** identificationID: Microvelia venustatis; identifiedBy: C. F. B. Floriano; **Event:** samplingProtocol: "D" Net; year: 2016; month: IX; **Record Level:** institutionCode: CEIOC; basisOfRecord: PreservedSpecimen
- **Type status:** Other material. **Occurrence:** recordedBy: C. F. B. Floriano; individualCount: 1; sex: male; lifeStage: adult; occurrenceID: B4BA163F-51BB-598A-8379-20B1F5848777; **Taxon:** genus: Microvelia; specificEpithet: venustatis; taxonRank: species; scientificNameAuthorship: Drake & Harris, 1933; **Location:** stateProvince: Paraná; municipality: Tuneiras do Oeste; locality: Reserva Biológica das Perobas, Rio Mouro; decimalLatitude: -23.8850; decimalLongitude: -52.8072; **Identification:** identificationID: Microvelia venustatis; identifiedBy: C. F. B. Floriano; **Event:** samplingProtocol: "D" Net; year: 2024; month: IV; **Record Level:** institutionCode: CEIOC; basisOfRecord: PreservedSpecimen
- **Type status:** Other material. **Occurrence:** recordedBy: C. F. B. Floriano; individualCount: 1; sex: male; lifeStage: adult; occurrenceID: B7F801FF-7B5B-5EA4-A368-7AA04A25B63A; **Taxon:** genus: Microvelia; specificEpithet: venustatis; taxonRank: species; scientificNameAuthorship: Drake & Harris, 1933; **Location:** stateProvince: Paraná; municipality: Marialva; locality: Tanque de peixe; decimalLatitude: -23.528; decimalLongitude: -51.7859; **Identification:** identificationID: Microvelia venustatis; identifiedBy: C. F. B. Floriano; **Event:** samplingProtocol: "D" Net; year: 2024; month: VI; **Record Level:** institutionCode: CEIOC; basisOfRecord: PreservedSpecimen
- **Type status:** Other material. **Occurrence:** recordedBy: C. F. B. Floriano; individualCount: 3; sex: female; lifeStage: adult; occurrenceID: 66DE73F4-02A9-5FF6-BC73-FC53AA0A7770; **Taxon:** genus: Microvelia; specificEpithet: venustatis; taxonRank: species; scientificNameAuthorship: Drake & Harris, 1933; **Location:** stateProvince: Paraná; municipality: Marialva; locality: Tanque de peixe; decimalLatitude: -23.528; decimalLongitude: -51.7859; **Identification:** identificationID: Microvelia venustatis; identifiedBy: C. F. B. Floriano; **Event:** samplingProtocol: "D" Net; year: 2024; month: VI; **Record Level:** institutionCode: CEIOC; basisOfRecord: PreservedSpecimen

##### Distribution

Colombia, Brazil, Peru, Paraguay and Argentina.

##### Notes

First records from Paraná (Fig. 5B, Fig. 6F). Previously reported from Santa Catarina (Drake and Plaumann 1955).

#### Rhagadotarsinae

Lundblad, 1933

B67C704D-CEB7-54CA-9C38-02E512BD0B31

#### Rheumatobates

Bergroth, 1892

7959F583-DB1B-53EF-935C-85038FDFB679

#### Rheumatobates bonariensis

(Berg, 1898)

0E52F62B-F942-5FCA-84D8-B113DE0E5776

##### Materials

- **Type status:** Other material. **Occurrence:** recordedBy: T. Roell & T. Polizei; individualCount: 3; sex: male; lifeStage: adult; occurrenceID: 1EDD8E84-18E0-5FCD-9D39-FB5AD2110ECE; **Taxon:** genus: Rheumatobates; specificEpithet: bonariensis; taxonRank: species; scientificNameAuthorship: (Berg, 1898); **Location:** stateProvince: Santa Catarina; municipality: Mafra; locality: Rio Negro; verbatimElevation: 790 m; decimalLatitude: -26.2117; decimalLongitude: -49.6286; **Identification:** identificationID: Rheumatobates bonariensis; identifiedBy: C. F. B. Floriano; **Event:** samplingProtocol: "D" Net; year: 2020; month: III; **Record Level:** institutionCode: CEIOC; basisOfRecord: PreservedSpecimen
- **Type status:** Other material. **Occurrence:** recordedBy: T. Roell & T. Polizei; individualCount: 6; sex: female; lifeStage: adult; occurrenceID: 92DF732E-DCAD-59B2-9A86-4D137CDEBFD4; **Taxon:** genus: Rheumatobates; specificEpithet: bonariensis; taxonRank: species; scientificNameAuthorship: (Berg, 1898); **Location:** stateProvince: Santa Catarina; municipality: Mafra; locality: Rio Negro; verbatimElevation: 790 m; decimalLatitude: -26.2117; decimalLongitude: -49.6286; **Identification:** identificationID: Rheumatobates bonariensis; identifiedBy: C. F. B. Floriano; **Event:** samplingProtocol: "D" Net; year: 2020; month: III; **Record Level:** institutionCode: CEIOC; basisOfRecord: PreservedSpecimen
- **Type status:** Other material. **Occurrence:** recordedBy: C. F. B. Floriano & T. Malacarne; individualCount: 5; sex: male; lifeStage: adult; occurrenceID: 8BE5F390-717B-5BFE-B0BC-C65B84A937B7; **Taxon:** genus: Rheumatobates; specificEpithet: bonariensis; taxonRank: species; scientificNameAuthorship: (Berg, 1898); **Location:** stateProvince: Santa Catarina; municipality: Blumenau; locality: Parque Nacional da Serra do Itajaí, Parque Natural Municipal Nascentes do Garcia, Rio Garcia; decimalLatitude: -27.0538; decimalLongitude: -49.0863; **Identification:** identificationID: Rheumatobates bonariensis; identifiedBy: C. F. B. Floriano; **Event:** samplingProtocol: "D" Net; year: 2017; month: IV; **Record Level:** institutionCode: CEIOC; basisOfRecord: PreservedSpecimen
- **Type status:** Other material. **Occurrence:** recordedBy: C. F. B. Floriano & T. Malacarne; individualCount: 4; sex: female; lifeStage: adult; occurrenceID: 9A55A25B-EBE6-5188-87D6-94D5B06B3450; **Taxon:** genus: Rheumatobates; specificEpithet: bonariensis; taxonRank: species; scientificNameAuthorship: (Berg, 1898); **Location:** stateProvince: Santa Catarina; municipality: Blumenau; locality: Parque Nacional da Serra do Itajaí, Parque Natural Municipal Nascentes do Garcia, Rio Garcia; decimalLatitude: -27.0538; decimalLongitude: -49.0863; **Identification:** identificationID: Rheumatobates bonariensis; identifiedBy: C. F. B. Floriano; **Event:** samplingProtocol: "D" Net; year: 2017; month: IV; **Record Level:** institutionCode: CEIOC; basisOfRecord: PreservedSpecimen
- **Type status:** Other material. **Occurrence:** recordedBy: C. F. B. Floriano; individualCount: 1; sex: male; lifeStage: adult; occurrenceID: 1CEE4684-36AB-54A4-8397-E381BD935204; **Taxon:** genus: Rheumatobates; specificEpithet: bonariensis; taxonRank: species; scientificNameAuthorship: (Berg, 1898); **Location:** stateProvince: Paraná; municipality: Paranaguá; locality: Ilha do Mel, richo próximo ao forte; decimalLatitude: -25.5123; decimalLongitude: -48.3171; **Identification:** identificationID: Rheumatobates bonariensis; identifiedBy: C. F. B. Floriano; **Event:** samplingProtocol: "D" Net; year: 2016; month: VI; **Record Level:** institutionCode: CEIOC; basisOfRecord: PreservedSpecimen
- **Type status:** Other material. **Occurrence:** recordedBy: C. F. B. Floriano & T. Polizei; individualCount: 1; sex: female; lifeStage: adult; occurrenceID: A9AB3F2A-8AD7-5C70-9D02-94050188C826; **Taxon:** genus: Rheumatobates; specificEpithet: bonariensis; taxonRank: species; scientificNameAuthorship: (Berg, 1898); **Location:** stateProvince: Rio Grande do Sul; municipality: Cambará do Sul; locality: Parque Nacional de Aparados da Serra, Córrego Preá; decimalLatitude: -29.17; decimalLongitude: -49.02; **Identification:** identificationID: Rheumatobates bonariensis; identifiedBy: C. F. B. Floriano; **Event:** samplingProtocol: "D" Net; year: 2016; month: IX; **Record Level:** institutionCode: CEIOC; basisOfRecord: PreservedSpecimen

##### Distribution

Brazil, Peru, Bolivia, Paraguay, Argentina and Uruguay.

##### Notes

First record from Paraná (Fig. 5C, Fig. 8A). Previously reported from Santa Catarina and Rio Grande do Sul (Hungerford 1954, Neri et al. 2005, Secretaria do Meio Ambiente e Infraestrutura do Estado do Rio Grande do Sul 2014, Rodrigues et al. 2021).

#### Rheumatobates crassifemur

Esaki, 1926

21178599-3283-5BDB-89E4-72A92FF547DD

#### Rheumatobates crassifemur crassifemur

Esaki, 1926

8EFAA180-3B1C-5298-BF70-E4E4C7BDED43

##### Materials

- **Type status:** Other material. **Occurrence:** recordedBy: C. F. B. Floriano; individualCount: 1; sex: male; lifeStage: adult; occurrenceID: 774B2E06-CA4C-5129-820A-4E8204F1C2E9; **Taxon:** genus: Rheumatobates; specificEpithet: crassifemur; infraspecificEpithet: crassifemur; taxonRank: Subspecies; scientificNameAuthorship: Esaki, 1926; **Location:** stateProvince: Paraná; municipality: Tuneiras do Oeste; locality: Reserva Biológica das Perobas, Lagoa; verbatimElevation: 422 m; decimalLatitude: -23.8894; decimalLongitude: -52.8144; **Identification:** identificationID: Rheumatobates crassifemur crassifemur; identifiedBy: C. F. B. Floriano; **Event:** samplingProtocol: "D" Net; year: 2024; month: IV; **Record Level:** institutionCode: CEIOC; basisOfRecord: PreservedSpecimen
- **Type status:** Other material. **Occurrence:** recordedBy: C. F. B. Floriano; individualCount: 4; sex: female; lifeStage: adult; occurrenceID: C86E98D7-E9E0-529D-8829-68F2EA14B737; **Taxon:** genus: Rheumatobates; specificEpithet: crassifemur; infraspecificEpithet: crassifemur; taxonRank: Subspecies; scientificNameAuthorship: Esaki, 1926; **Location:** stateProvince: Paraná; municipality: Tuneiras do Oeste; locality: Reserva Biológica das Perobas, Lagoa; verbatimElevation: 422 m; decimalLatitude: -23.8894; decimalLongitude: -52.8144; **Identification:** identificationID: Rheumatobates crassifemur crassifemur; identifiedBy: C. F. B. Floriano; **Event:** samplingProtocol: "D" Net; year: 2024; month: IV; **Record Level:** institutionCode: CEIOC; basisOfRecord: PreservedSpecimen
- **Type status:** Other material. **Occurrence:** recordedBy: C. F. B. Floriano; individualCount: 2; sex: male; lifeStage: adult; occurrenceID: 90078FB8-B019-5422-B2C8-AEC7E3F50D7E; **Taxon:** genus: Rheumatobates; specificEpithet: crassifemur; infraspecificEpithet: crassifemur; taxonRank: Subspecies; scientificNameAuthorship: Esaki, 1926; **Location:** stateProvince: Paraná; municipality: Marialva; locality: Tanque de peixe; decimalLatitude: -23.528; decimalLongitude: -51.785; **Identification:** identificationID: Rheumatobates crassifemur crassifemur; identifiedBy: C. F. B. Floriano; **Event:** samplingProtocol: "D" Net; year: 2024; month: VI; **Record Level:** institutionCode: CEIOC; basisOfRecord: PreservedSpecimen
- **Type status:** Other material. **Occurrence:** recordedBy: C. F. B. Floriano; individualCount: 3; sex: female; lifeStage: adult; occurrenceID: 908CE8E5-AE3B-5853-A07C-441568DEABF5; **Taxon:** genus: Rheumatobates; specificEpithet: crassifemur; infraspecificEpithet: crassifemur; taxonRank: Subspecies; scientificNameAuthorship: Esaki, 1926; **Location:** stateProvince: Paraná; municipality: Marialva; locality: Tanque de peixe; decimalLatitude: -23.528; decimalLongitude: -51.785; **Identification:** identificationID: Rheumatobates crassifemur crassifemur; identifiedBy: C. F. B. Floriano; **Event:** samplingProtocol: "D" Net; year: 2024; month: VI; **Record Level:** institutionCode: CEIOC; basisOfRecord: PreservedSpecimen

##### Distribution

Panama, Colombia, Brazil, Bolivia, Paraguay and Argentina.

##### Notes

First records from Paraná (Fig. 5C, Fig. 8B). Previously reported from Rio Grande do Sul (Rodrigues et al. 2021).

#### Rheumatobates minutus

Hungerford, 1936

A5C8369E-C4EA-5B7D-894A-B4EE9F15B6FC

#### Rheumatobates minutus flavidus

Drake & Harris, 1942

87690787-73AE-5856-9B83-05409D305787

##### Materials

- **Type status:** Other material. **Occurrence:** recordedBy: C. F. B. Floriano; individualCount: 1; sex: male; lifeStage: adult; occurrenceID: C883F1A0-9AAA-51D5-99F2-FF3E089FA785; **Taxon:** genus: Rheumatobates; specificEpithet: minutus; infraspecificEpithet: flavidus; taxonRank: species; scientificNameAuthorship: Drake & Harris, 1942; **Location:** stateProvince: Paraná; municipality: Tuneiras do Oeste; locality: Reserva Biológica das Perobas, Rio Mouro; decimalLatitude: -23.8850; decimalLongitude: -52.8072; **Identification:** identificationID: Rheumatobates minutus flavidus; identifiedBy: C. F. B. Floriano; **Event:** samplingProtocol: "D" Net; year: 2024; month: IV; **Record Level:** institutionCode: CEIOC; basisOfRecord: PreservedSpecimen

##### Distribution

Costa Rica, Panama, Colombia, Brazil, Peru, Bolivia and Argentina.

##### Notes

First record from Paraná and the South Region of Brazil (Fig. 5C, Fig. 8C).

#### Trepobatinae

Matsuda, 1960

E1D51263-7104-58A1-BD9D-2FC2B65E3044

#### Halobatopsis

Bianchi, 1896

890FAA9C-18B8-5380-B90F-9DF4B4E50C1C

#### Halobatopsis platensis

(Berg, 1879)

B34FFDD4-8FEF-5124-A144-9D1BCCC0762D

##### Materials

- **Type status:** Other material. **Occurrence:** recordedBy: C. F. B. Floriano; individualCount: 1; sex: male; lifeStage: adult; occurrenceID: 58B051FA-DBB6-543F-BE51-B06F90813830; **Taxon:** genus: Halobatopsis; specificEpithet: platensis; taxonRank: species; scientificNameAuthorship: (Berg, 1879); **Location:** stateProvince: Paraná; municipality: Morretes; locality: Rio Nhudiaquara; decimalLatitude: -25.483; decimalLongitude: -48.820; **Identification:** identificationID: Halobatopsis platensis; identifiedBy: C. F. B. Floriano; **Event:** samplingProtocol: "D" Net; year: 2017; month: IX; **Record Level:** institutionCode: CEIOC; basisOfRecord: PreservedSpecimen
- **Type status:** Other material. **Occurrence:** recordedBy: C. F. B. Floriano; individualCount: 1; sex: female; lifeStage: adult; occurrenceID: E3069E1E-D6D4-5FB4-A022-E2006D2DFBC5; **Taxon:** genus: Halobatopsis; specificEpithet: platensis; taxonRank: species; scientificNameAuthorship: (Berg, 1879); **Location:** stateProvince: Paraná; municipality: Morretes; locality: Rio Nhudiaquara; decimalLatitude: -25.483; decimalLongitude: -48.820; **Identification:** identificationID: Halobatopsis platensis; identifiedBy: C. F. B. Floriano; **Event:** samplingProtocol: "D" Net; year: 2017; month: IX; **Record Level:** institutionCode: CEIOC; basisOfRecord: PreservedSpecimen
- **Type status:** Other material. **Occurrence:** recordedBy: R. P. R. Canejo, J. M. S. Rodrigues, M. S. L. Alexandre, L. P. Pereira; individualCount: 1; sex: male; lifeStage: adult; occurrenceID: 79C4F1D8-D716-5EE3-89C3-96A9AF8CF481; **Taxon:** genus: Halobatopsis; specificEpithet: platensis; taxonRank: species; scientificNameAuthorship: (Berg, 1879); **Location:** stateProvince: Paraná; municipality: Jaguariaíva; locality: Rio Diamante; verbatimElevation: 1127 m; decimalLatitude: -24.3589; decimalLongitude: -49.8126; **Identification:** identificationID: Halobatopsis platensis; identifiedBy: C. F. B. Floriano; **Event:** samplingProtocol: "D" Net; year: 2023; month: X; **Record Level:** institutionCode: CEIOC; basisOfRecord: PreservedSpecimen
- **Type status:** Other material. **Occurrence:** recordedBy: R. P. R. Canejo, J. M. S. Rodrigues, M. S. L. Alexandre, L.P. Pereira; individualCount: 2; sex: female; lifeStage: adult; occurrenceID: 1B5C9808-1039-5BB9-9AD6-7C65C6EAA3BA; **Taxon:** genus: Halobatopsis; specificEpithet: platensis; taxonRank: species; scientificNameAuthorship: (Berg, 1879); **Location:** stateProvince: Paraná; municipality: Jaguariaíva; locality: Rio Diamante; verbatimElevation: 1127 m; decimalLatitude: -24.3589; decimalLongitude: -49.8126; **Identification:** identificationID: Halobatopsis platensis; identifiedBy: C. F. B. Floriano; **Event:** samplingProtocol: "D" Net; year: 2023; month: X; **Record Level:** institutionCode: CEIOC; basisOfRecord: PreservedSpecimen
- **Type status:** Other material. **Occurrence:** recordedBy: R. P. R. Canejo, J. M. S. Rodrigues, M. S. L. Alexandre, L.P. Pereira; individualCount: 2; sex: male; lifeStage: adult; occurrenceID: B43D0868-A4A9-574E-A229-C5904CE3221E; **Taxon:** genus: Halobatopsis; specificEpithet: platensis; taxonRank: species; scientificNameAuthorship: (Berg, 1879); **Location:** stateProvince: Paraná; municipality: Ponta Grossa; locality: Rio Pintagui; verbatimElevation: 850 m; decimalLatitude: -25.0297; decimalLongitude: -50.1008; **Identification:** identificationID: Halobatopsis platensis; identifiedBy: C. F. B. Floriano; **Event:** samplingProtocol: "D" Net; year: 2023; month: X; **Record Level:** institutionCode: CEIOC; basisOfRecord: PreservedSpecimen
- **Type status:** Other material. **Occurrence:** recordedBy: R. P. R. Canejo, J. M. S. Rodrigues, M. S. L. Alexandre, L. P. Pereira; individualCount: 2; sex: female; lifeStage: adult; occurrenceID: 09D14238-22E3-556E-A0DB-457E5F9EC335; **Taxon:** genus: Halobatopsis; specificEpithet: platensis; taxonRank: species; scientificNameAuthorship: (Berg, 1879); **Location:** stateProvince: Paraná; municipality: Ponta Grossa; locality: Rio Pintagui; verbatimElevation: 850 m; decimalLatitude: -25.0297; decimalLongitude: -50.1008; **Identification:** identificationID: Halobatopsis platensis; identifiedBy: C. F. B. Floriano; **Event:** samplingProtocol: "D" Net; year: 2023; month: X; **Record Level:** institutionCode: CEIOC; basisOfRecord: PreservedSpecimen

##### Distribution

Brazil, Peru, Paraguay, Argentina and Uruguay.

##### Notes

Previously reported from Paraná and Rio Grande do Sul (Nieser 1970, Moreira and Campos 2012, Secretaria do Meio Ambiente e Infraestrutura do Estado do Rio Grande do Sul 2014, Rodrigues et al. 2021).

#### Halobatopsis spiniventris

Drake & Harris, 1936

801A578D-1491-5F0D-9C8E-F071A337EAF8

##### Materials

- **Type status:** Other material. **Occurrence:** recordedBy: C. F. B. Floriano & T. Malacarne; individualCount: 1; sex: male; lifeStage: adult; occurrenceID: 46CBA14E-2BA1-5D9E-9B3E-DD4FBB949B68; **Taxon:** genus: Halobatopsis; specificEpithet: spiniventris; taxonRank: species; scientificNameAuthorship: Drake & Harris, 1936; **Location:** stateProvince: Santa Catarina; municipality: Blumenau; locality: Parque Nacional da Serra do Itajaí, Parque Natural Municipal Nascentes do Garcia, Rio Garcia; decimalLatitude: -27.0538; decimalLongitude: -49.0863; **Identification:** identificationID: Halobatopsis spiniventris; identifiedBy: C. F. B. Floriano; **Event:** samplingProtocol: "D" Net; year: 2017; month: IV; **Record Level:** institutionCode: CEIOC; basisOfRecord: PreservedSpecimen
- **Type status:** Other material. **Occurrence:** recordedBy: C. F. B. Floriano & T. Malacarne; individualCount: 4; sex: female; lifeStage: adult; occurrenceID: FD521ADB-D1E1-5423-B88E-1A24C4820362; **Taxon:** genus: Halobatopsis; specificEpithet: spiniventris; taxonRank: species; scientificNameAuthorship: Drake & Harris, 1936; **Location:** stateProvince: Santa Catarina; municipality: Blumenau; locality: Parque Nacional da Serra do Itajaí, Parque Natural Municipal Nascentes do Garcia, Rio Garcia; decimalLatitude: -27.0538; decimalLongitude: -49.0863; **Identification:** identificationID: Halobatopsis spiniventris; identifiedBy: C. F. B. Floriano; **Event:** samplingProtocol: "D" Net; year: 2017; month: IV; **Record Level:** institutionCode: CEIOC; basisOfRecord: PreservedSpecimen
- **Type status:** Other material. **Occurrence:** recordedBy: T. Roell & T. Polizei; individualCount: 3; sex: male; lifeStage: adult; occurrenceID: 728A99CD-D845-5DA7-AD4A-72EFFE567EDB; **Taxon:** genus: Halobatopsis; specificEpithet: spiniventris; taxonRank: species; scientificNameAuthorship: Drake & Harris, 1936; **Location:** stateProvince: Santa Catarina; municipality: Mafra; decimalLatitude: -26.2127; decimalLongitude: -49.6252; **Identification:** identificationID: Halobatopsis spiniventris; identifiedBy: C. F. B. Floriano; **Event:** samplingProtocol: "D" Net; year: 2019; month: XI; **Record Level:** institutionCode: CEIOC; basisOfRecord: PreservedSpecimen
- **Type status:** Other material. **Occurrence:** recordedBy: T. Roell & T. Polizei; individualCount: 2; sex: female; lifeStage: adult; occurrenceID: 5B95E444-49B6-5D9C-9A5C-E3479B877DF0; **Taxon:** genus: Halobatopsis; specificEpithet: spiniventris; taxonRank: species; scientificNameAuthorship: Drake & Harris, 1936; **Location:** stateProvince: Santa Catarina; municipality: Mafra; decimalLatitude: -26.2127; decimalLongitude: -49.6252; **Identification:** identificationID: Halobatopsis spiniventris; identifiedBy: C. F. B. Floriano; **Event:** samplingProtocol: "D" Net; year: 2019; month: XI; **Record Level:** institutionCode: CEIOC; basisOfRecord: PreservedSpecimen
- **Type status:** Other material. **Occurrence:** recordedBy: C. F. B. Floriano & T. Polizei; individualCount: 2; sex: male; lifeStage: adult; occurrenceID: BCEABE53-3F04-5F3A-98FC-D7439C8671D0; **Taxon:** genus: Halobatopsis; specificEpithet: spiniventris; taxonRank: species; scientificNameAuthorship: Drake & Harris, 1936; **Location:** stateProvince: Santa Catarina; municipality: São Bonifácio; locality: Rio Capivari; decimalLatitude: -28.01; decimalLongitude: -49.02; **Identification:** identificationID: Halobatopsis spiniventris; identifiedBy: C. F. B. Floriano; **Event:** samplingProtocol: "D" Net; year: 2016; month: IX; **Record Level:** institutionCode: CEIOC; basisOfRecord: PreservedSpecimen
- **Type status:** Other material. **Occurrence:** recordedBy: C. F. B. Floriano & T. Polizei; individualCount: 2; sex: male; lifeStage: adult; occurrenceID: CE4D025F-543D-5147-812E-B5CED3687537; **Taxon:** genus: Halobatopsis; specificEpithet: spiniventris; taxonRank: species; scientificNameAuthorship: Drake & Harris, 1936; **Location:** stateProvince: Santa Catarina; municipality: Rancho Queimado; locality: Rio das Antas; decimalLatitude: -27.67; decimalLongitude: -49.01; **Identification:** identificationID: Halobatopsis spiniventris; identifiedBy: C. F. B. Floriano; **Event:** samplingProtocol: "D" Net; year: 2016; month: IX; **Record Level:** institutionCode: CEIOC; basisOfRecord: PreservedSpecimen

##### Distribution

Brazil, Paraguay and Argentina.

##### Notes

Previously reported from Paraná, Santa Catarina and Rio Grande do Sul (Nieser and Melo 1999, Moreira and Campos 2012, Cordeiro and Moreira 2015, Rodrigues et al. 2021).

#### Metrobates

Uhler, 1871

192CD5D3-3D71-59A6-8A6D-30DC9B3F1B7A

#### Metrobates plaumanni plaumanni

Hungerford, 1951

5FE50731-391B-5A32-9BCC-414B07E4A010

##### Materials

- **Type status:** Other material. **Occurrence:** recordedBy: C. F. B. Floriano; individualCount: 18; sex: male; lifeStage: adult; occurrenceID: 4FE941C4-41DA-5743-BCC3-8E2E1667A8D5; **Taxon:** genus: Metrobates; specificEpithet: plaumanni; infraspecificEpithet: plaumanni; taxonRank: subspecies; scientificNameAuthorship: Hungerford, 1951; **Location:** stateProvince: Paraná; municipality: Morretes; locality: Rio Nhudiaquara; decimalLatitude: -25.483; decimalLongitude: -48.820; **Identification:** identificationID: Metrobates plaumanni; identifiedBy: C. F. B. Floriano; **Event:** samplingProtocol: "D" Net; year: 2017; month: IX; **Record Level:** institutionCode: CEIOC; basisOfRecord: PreservedSpecimen
- **Type status:** Other material. **Occurrence:** recordedBy: C. F. B. Floriano; individualCount: 9; sex: female; lifeStage: adult; occurrenceID: E064DF73-D90D-5A51-A9D6-6E75C73DC5FB; **Taxon:** genus: Metrobates; specificEpithet: plaumanni; infraspecificEpithet: plaumanni; taxonRank: subspecies; scientificNameAuthorship: Hungerford, 1951; **Location:** stateProvince: Paraná; municipality: Morretes; locality: Rio Nhudiaquara; decimalLatitude: -25.483; decimalLongitude: -48.820; **Identification:** identificationID: Metrobates plaumanni; identifiedBy: C. F. B. Floriano; **Event:** samplingProtocol: "D" Net; year: 2017; month: IX; **Record Level:** institutionCode: CEIOC; basisOfRecord: PreservedSpecimen
- **Type status:** Other material. **Occurrence:** recordedBy: C. F. B. Floriano & T. Malacarne; individualCount: 3; sex: male; lifeStage: adult; occurrenceID: A049C4E1-1F12-590D-8632-6B7510807CC8; **Taxon:** genus: Metrobates; specificEpithet: plaumanni; infraspecificEpithet: plaumanni; taxonRank: subspecies; scientificNameAuthorship: Hungerford, 1951; **Location:** stateProvince: Santa Catarina; municipality: Indaial; locality: Parque Nacional da Serra do Itajaí, Pesque Pague do Wili; decimalLatitude: -27.0158; decimalLongitude: -49.1660; **Identification:** identificationID: Metrobates plaumanni; identifiedBy: C. F. B. Floriano; **Event:** samplingProtocol: "D" Net; year: 2017; month: IV; **Record Level:** institutionCode: CEIOC; basisOfRecord: PreservedSpecimen
- **Type status:** Other material. **Occurrence:** recordedBy: C. F. B. Floriano & T. Malacarne; individualCount: 4; sex: female; lifeStage: adult; occurrenceID: C30CACAD-6DB0-5E70-ADDA-B7F8C55B1C84; **Taxon:** genus: Metrobates; specificEpithet: plaumanni; infraspecificEpithet: plaumanni; taxonRank: subspecies; scientificNameAuthorship: Hungerford, 1951; **Location:** stateProvince: Santa Catarina; municipality: Indaial; locality: Parque Nacional da Serra do Itajaí, Pesque Pague do Wili; decimalLatitude: -27.0158; decimalLongitude: -49.1660; **Identification:** identificationID: Metrobates plaumanni; identifiedBy: C. F. B. Floriano; **Event:** samplingProtocol: "D" Net; year: 2017; month: IV; **Record Level:** institutionCode: CEIOC; basisOfRecord: PreservedSpecimen
- **Type status:** Other material. **Occurrence:** recordedBy: T. Roell & T. Polizei; individualCount: 15; sex: male; lifeStage: adult; occurrenceID: 073BB72C-A507-5FC5-80BD-D471C2017EA4; **Taxon:** genus: Metrobates; specificEpithet: plaumanni; infraspecificEpithet: plaumanni; taxonRank: subspecies; scientificNameAuthorship: Hungerford, 1951; **Location:** stateProvince: Santa Catarina; municipality: Mafra; locality: Rio Negro; decimalLatitude: -26.2117; decimalLongitude: -49.6286; **Identification:** identificationID: Metrobates plaumanni; identifiedBy: C. F. B. Floriano; **Event:** samplingProtocol: "D" Net; year: 2020; month: III; **Record Level:** institutionCode: CEIOC; basisOfRecord: PreservedSpecimen
- **Type status:** Other material. **Occurrence:** recordedBy: T. Roell & T. Polizei; individualCount: 18; sex: female; lifeStage: adult; occurrenceID: 1FED1D1E-6FCC-5D48-809C-117B5BD9CA57; **Taxon:** genus: Metrobates; specificEpithet: plaumanni; infraspecificEpithet: plaumanni; taxonRank: subspecies; scientificNameAuthorship: Hungerford, 1951; **Location:** stateProvince: Santa Catarina; municipality: Mafra; locality: Rio Negro; decimalLatitude: -26.2117; decimalLongitude: -49.6286; **Identification:** identificationID: Metrobates plaumanni; identifiedBy: C. F. B. Floriano; **Event:** samplingProtocol: "D" Net; year: 2020; month: III; **Record Level:** institutionCode: CEIOC; basisOfRecord: PreservedSpecimen
- **Type status:** Other material. **Occurrence:** recordedBy: T. Roell & T. Polizei; individualCount: 3; sex: male; lifeStage: adult; occurrenceID: 76770918-C82A-5D25-B4F0-348EAF645CFE; **Taxon:** genus: Metrobates; specificEpithet: plaumanni; infraspecificEpithet: plaumanni; taxonRank: subspecies; scientificNameAuthorship: Hungerford, 1951; **Location:** stateProvince: Santa Catarina; municipality: Rio Negrinho; locality: Rio do Bugres; decimalLatitude: -26.2769; decimalLongitude: -49.5344; **Identification:** identificationID: Metrobates plaumanni; identifiedBy: C. F. B. Floriano; **Event:** samplingProtocol: "D" Net; year: 2020; month: III; **Record Level:** institutionCode: CEIOC; basisOfRecord: PreservedSpecimen
- **Type status:** Other material. **Occurrence:** recordedBy: T. Roell & T. Polizei; individualCount: 2; sex: female; lifeStage: adult; occurrenceID: 3F275DE1-BC73-52E3-9176-75B9FAD0B402; **Taxon:** genus: Metrobates; specificEpithet: plaumanni; infraspecificEpithet: plaumanni; taxonRank: subspecies; scientificNameAuthorship: Hungerford, 1951; **Location:** stateProvince: Santa Catarina; municipality: Rio Negrinho; locality: Rio do Bugres; decimalLatitude: -26.2769; decimalLongitude: -49.5344; **Identification:** identificationID: Metrobates plaumanni; identifiedBy: C. F. B. Floriano; **Event:** samplingProtocol: "D" Net; year: 2020; month: III; **Record Level:** institutionCode: CEIOC; basisOfRecord: PreservedSpecimen

##### Distribution

Brazil, Argentina and Uruguay.

##### Notes

Previoulsy reported from Paraná and Santa Catarina (Hungerford 1951, Armisén et al. 2022).

#### Hebridae

Amyot & Serville, 1843

DE16D5B0-3D1B-513A-A040-A63F30A595A6

#### Hebrinae

Amyot & Serville, 1843

A2F4BF6B-F8FF-5A31-A290-42AA700EC10B

#### Hebrus

Curtis, 1833

04EF7EFA-B70D-563D-BAB9-2C873375864D

#### Hebrus parvulus

Stål, 1860

789DFCAE-5B35-5C77-9C4F-E2A0515F7D85

##### Materials

- **Type status:** Other material. **Occurrence:** recordedBy: C. F. B. Floriano & T. Polizei; individualCount: 1; sex: male; lifeStage: adult; occurrenceID: D6D38ACB-AFB7-5121-B768-CF7FB46181E8; **Taxon:** genus: Hebrus; specificEpithet: parvulus; taxonRank: species; scientificNameAuthorship: Stål, 1860; **Location:** stateProvince: Santa Catarina; municipality: São Bonifácio; locality: Rio Moll; decimalLatitude: -27.89; decimalLongitude: -48.92; **Identification:** identificationID: Hebrus parvulus; identifiedBy: C. L. FRANCO; **Event:** samplingProtocol: "D" Net; year: 2016; month: IX; **Record Level:** institutionCode: CEIOC; basisOfRecord: PreservedSpecimen

##### Distribution

Brazil.

##### Notes

**Figure 7.**
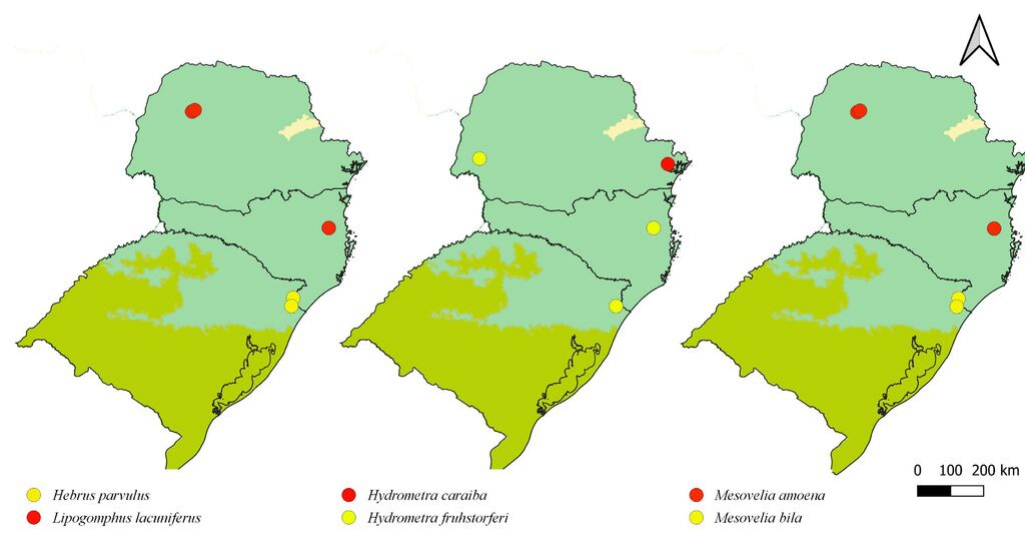
Maps showing new records of Hebridae, Hydrometridae and Mesoveliidae from the South Region of Brazil.

**Figure 8.**
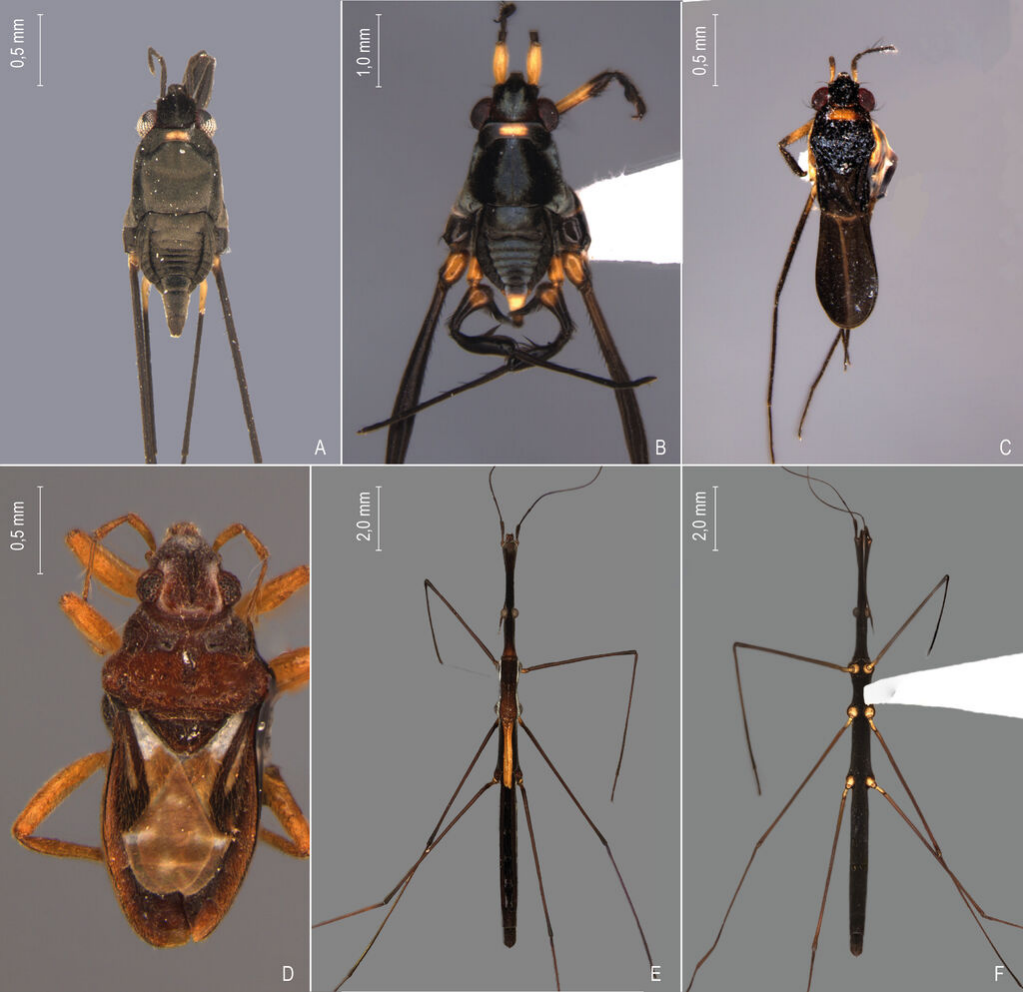
Gerridae, Hebridae and Hydrometridae from the South Region of Brazil, male, habitus. **A**–**E**: Dorsal View. **F**: Ventral view. **A** *Rheumatobates bonariensis*; **B** *Rheumatobates crassifemur crassifemur*; **C** *Rheumatobates minutus flavidus*; **D** *Hebrus parvulus*; **E**, **F** *Hydrometra fruhstorferi*.

First record from Santa Catarina and the South Region of Brazil (Fig. 7A, Fig. 8D).

#### Lipogomphus

Berg, 1879

3B14A4FC-0310-57F6-A09B-95413396152D

#### Lipogomphus lacuniferus

Berg, 1879

932582B9-4A75-5B6A-B4E2-45D5F567DF6F

##### Materials

- **Type status:** Other material. **Occurrence:** recordedBy: C. F. B. Floriano & T. Malacarne; individualCount: 1; sex: male; lifeStage: adult; occurrenceID: B78FCEAC-6ACD-5CCD-AC25-4215CE2434F7; **Taxon:** genus: Lipogomphus; specificEpithet: lacuniferus; taxonRank: species; scientificNameAuthorship: Berg, 1879; **Location:** stateProvince: Santa Catarina; municipality: Blumenau; locality: Parque Nacional da Serra do Itajaí, Parque Natural Municipal Nascentes do Garcia, Lagoa Negra; decimalLatitude: -27.0532; decimalLongitude: -49.0878; **Identification:** identificationID: Lipogomphus lacuniferus; identifiedBy: C. L. FRANCO; **Event:** samplingProtocol: "D" Net; year: 2017; month: IV; **Record Level:** institutionCode: CEIOC; basisOfRecord: PreservedSpecimen

##### Distribution

Brazil, Bolivia, Paraguay, Argentina and Uruguay.

##### Notes

First record from Santa Catarina and the South Region of Brazil (Fig. 7A).

#### Hydrometridae

Billberg, 1820

261B2B14-54B1-58C3-B9BB-2CB055FE36E9

#### Hydrometrinae

Billberg, 1820

BEA7C983-4AB1-57B3-B55D-AEC9A1C4E50B

#### Hydrometra

Latreille, 1797

C5866759-76C5-582B-8731-56AA998FB055

#### Hydrometra argentina

Berg, 1879

39D88DBA-C96D-52CE-A3ED-8AE37D1D14DB

##### Materials

- **Type status:** Other material. **Occurrence:** recordedBy: C. F. B. Floriano & T. Malacarne; individualCount: 1; sex: male; lifeStage: adult; occurrenceID: A9356B64-3A24-5B4C-BDBC-D11016DADDD4; **Taxon:** genus: Hydrometra; specificEpithet: argentina; taxonRank: species; scientificNameAuthorship: Berg, 1879; **Location:** stateProvince: Santa Catarina; municipality: Blumenau; locality: Parque Nacional da Serra do Itajaí, Parque Natural Municipal Nascentes do Garcia, Rio Garcia; decimalLatitude: -27.0538; decimalLongitude: -49.0863; **Identification:** identificationID: Hydrometra argentina; identifiedBy: I. R. S. Cordeiro; **Event:** samplingProtocol: "D" Net; year: 2017; month: IV; **Record Level:** institutionCode: CEIOC; basisOfRecord: PreservedSpecimen
- **Type status:** Other material. **Occurrence:** recordedBy: C. F. B. Floriano & T. Malacarne; individualCount: 1; sex: female; lifeStage: adult; occurrenceID: AD347EB4-5E2B-5F29-BBF2-57BDCC577267; **Taxon:** genus: Hydrometra; specificEpithet: argentina; taxonRank: species; scientificNameAuthorship: Berg, 1879; **Location:** stateProvince: Santa Catarina; municipality: Blumenau; locality: Parque Nacional da Serra do Itajaí, Parque Natural Municipal Nascentes do Garcia, Rio Garcia; decimalLatitude: -27.0538; decimalLongitude: -49.0863; **Identification:** identificationID: Hydrometra argentina; identifiedBy: I. R. S. Cordeiro; **Event:** samplingProtocol: "D" Net; year: 2017; month: IV; **Record Level:** institutionCode: CEIOC; basisOfRecord: PreservedSpecimen
- **Type status:** Other material. **Occurrence:** recordedBy: C. F. B. Floriano & T. Malacarne; individualCount: 1; sex: male; lifeStage: adult; occurrenceID: F9509CE4-C871-544D-962E-E8F06DA075E9; **Taxon:** genus: Hydrometra; specificEpithet: argentina; taxonRank: species; scientificNameAuthorship: Berg, 1879; **Location:** stateProvince: Santa Catarina; municipality: Blumenau; locality: Parque Nacional da Serra do Itajaí, Parque Natural Municipal Nascentes do Garcia, Lagoa Negra; decimalLatitude: -27.0532; decimalLongitude: -49.0878; **Identification:** identificationID: Hydrometra argentina; identifiedBy: I. R. S. Cordeiro; **Event:** samplingProtocol: "D" Net; year: 2017; month: IV; **Record Level:** institutionCode: CEIOC; basisOfRecord: PreservedSpecimen
- **Type status:** Other material. **Occurrence:** recordedBy: C. F. B. Floriano & T. Polizei; individualCount: 1; sex: female; lifeStage: adult; occurrenceID: 39F92817-EBE6-5865-AE53-509966D32BB4; **Taxon:** genus: Hydrometra; specificEpithet: argentina; taxonRank: species; scientificNameAuthorship: Berg, 1879; **Location:** stateProvince: Rio Grande do Sul; municipality: Cambará do Sul; locality: Parque Nacional de Aparados da Serra, lagoa no caminho para o canion Fortaleza; decimalLatitude: -29.177; decimalLongitude: -50.098; **Identification:** identificationID: Hydrometra argentina; identifiedBy: I. R. S. Cordeiro; **Event:** samplingProtocol: "D" Net; year: 2016; month: IX; **Record Level:** institutionCode: CEIOC; basisOfRecord: PreservedSpecimen
- **Type status:** Other material. **Occurrence:** recordedBy: C. F. B. Floriano; individualCount: 2; sex: female; lifeStage: adult; occurrenceID: 17502F51-9099-5F25-98FD-3451E14AE4EF; **Taxon:** genus: Hydrometra; specificEpithet: argentina; taxonRank: species; scientificNameAuthorship: Berg, 1879; **Location:** stateProvince: Paraná; municipality: Curitiba; locality: Lagoa do Jardim Botânico; decimalLatitude: -25.441; decimalLongitude: -49.240; **Identification:** identificationID: Hydrometra argentina; identifiedBy: I. R. S. Cordeiro; **Event:** samplingProtocol: "D" Net; year: 2016; month: XI; **Record Level:** institutionCode: CEIOC; basisOfRecord: PreservedSpecimen

##### Distribution

Panama, Trinidad & Tobago, Colombia, Venezuela, Suriname, Brazil, Ecuador, Peru, Bolivia, Chile, Paraguay, Argentina and Uruguay.

##### Notes

Previoulsy reported from Paraná, Santa Catarina and Rio Grande do Sul (Jaczewski 1928a, Neri et al. 2005, University of California, Berkeley 2009, Secretaria do Meio Ambiente e Infraestrutura do Estado do Rio Grande do Sul 2014, Rodrigues et al. 2021).

#### Hydrometra caraiba

Guérin-Méneville, 1856

FEA12D8E-182F-5A10-9019-E4C0494810FF

##### Materials

- **Type status:** Other material. **Occurrence:** recordedBy: A. C. Domahovski; individualCount: 1; sex: male; lifeStage: adult; occurrenceID: CDD2778C-0F36-5122-82D9-820D9C48BF5C; **Taxon:** genus: Hydrometra; specificEpithet: caraiba; taxonRank: species; scientificNameAuthorship: Guérin-Méneville, 1856; **Location:** stateProvince: Paraná; municipality: Antonina; locality: Reserva Natural da Guaricica; decimalLatitude: -25.3160; decimalLongitude: -48.6960; **Identification:** identificationID: Hydrometra caraiba; identifiedBy: I. R. S. Cordeiro; **Event:** year: 2017; month: X; **Record Level:** institutionCode: DZRJ; basisOfRecord: PreservedSpecimen
- **Type status:** Other material. **Occurrence:** recordedBy: A. C. Domahovski; individualCount: 3; sex: female; lifeStage: adult; occurrenceID: A6E626BD-82D9-5B37-AEF9-AC439981CC6B; **Taxon:** genus: Hydrometra; specificEpithet: caraiba; taxonRank: species; scientificNameAuthorship: Guérin-Méneville, 1856; **Location:** stateProvince: Paraná; municipality: Antonina; locality: Reserva Natural da Guaricica; decimalLatitude: -25.3160; decimalLongitude: -48.6960; **Identification:** identificationID: Hydrometra caraiba; identifiedBy: I. R. S. Cordeiro; **Event:** year: 2017; month: X; **Record Level:** institutionCode: DZRJ; basisOfRecord: PreservedSpecimen

##### Distribution

Mexico, Guatemala, Cuba, Haiti, Jamaica, Puerto Rico, Guadeloupe, Martinique, Honduras, Nicaragua, El Salvador, Costa Rica, Panama, Trinidad & Tobago, Colombia, Venezuela, Guyana, French Guiana, Brazil, Ecuador and Peru.

##### Notes

First records for Paraná and the South Region of Brazil (Fig. 7B).

#### Hydrometra fruhstorferi

Hungerford & Evans, 1934

AD089E75-A64D-5C5D-8666-AA614CE2A724

##### Materials

- **Type status:** Other material. **Occurrence:** recordedBy: C. F. B. Floriano & T. Malacarne; individualCount: 2; sex: male; lifeStage: adult; occurrenceID: 0D3091F3-BC60-57E6-B679-AF604587EAED; **Taxon:** genus: Hydrometra; specificEpithet: fruhstorferi; taxonRank: species; scientificNameAuthorship: Hungerford & Evans, 1934; **Location:** stateProvince: Santa Catarina; municipality: Blumenau; locality: Parque Nacional da Serra do Itajaí, Parque Natural Municipal Nascentes do Garcia, Rio Garcia; decimalLatitude: -27.0538; decimalLongitude: -49.0863; **Identification:** identificationID: Hydrometra fruhstorferi; identifiedBy: I. R. S. Cordeiro; **Event:** samplingProtocol: "D" Net; year: 2017; month: IV; **Record Level:** institutionCode: CEIOC; basisOfRecord: PreservedSpecimen
- **Type status:** Other material. **Occurrence:** recordedBy: C. F. B. Floriano & T. Malacarne; individualCount: 2; sex: female; lifeStage: adult; occurrenceID: 6D1DF00D-A13B-5E20-9825-F8C79EE5DAD3; **Taxon:** genus: Hydrometra; specificEpithet: fruhstorferi; taxonRank: species; scientificNameAuthorship: Hungerford & Evans, 1934; **Location:** stateProvince: Santa Catarina; municipality: Blumenau; locality: Parque Nacional da Serra do Itajaí, Parque Natural Municipal Nascentes do Garcia, Rio Garcia; decimalLatitude: -27.0538; decimalLongitude: -49.0863; **Identification:** identificationID: Hydrometra fruhstorferi; identifiedBy: I. R. S. Cordeiro; **Event:** samplingProtocol: "D" Net; year: 2017; month: IV; **Record Level:** institutionCode: CEIOC; basisOfRecord: PreservedSpecimen
- **Type status:** Other material. **Occurrence:** recordedBy: C. F. B. Floriano & T. Polizei; individualCount: 2; sex: female; lifeStage: adult; occurrenceID: A1C53641-1827-5C63-B15F-6B715B514555; **Taxon:** genus: Hydrometra; specificEpithet: fruhstorferi; taxonRank: species; scientificNameAuthorship: Hungerford & Evans, 1934; **Location:** stateProvince: Rio Grande do Sul; municipality: Cambará do Sul; locality: Parque Nacional de Aparados da Serra, Córrego Preá; decimalLatitude: -29.17; decimalLongitude: -50.09; **Identification:** identificationID: Hydrometra fruhstorferi; identifiedBy: I. R. S. Cordeiro; **Event:** samplingProtocol: "D" Net; year: 2016; month: IX; **Record Level:** institutionCode: CEIOC; basisOfRecord: PreservedSpecimen

##### Distribution

Brazil, Bolivia, Paraguay and Argentina.

##### Notes

First record from Rio Grande do Sul (Fig. 8D-E, Fig. 7B). Previously reported from Paraná and Santa Catarina (Drake 1952b, Cordeiro and Moreira 2015).

#### Mesoveliidae

Douglas & Scott, 1867

B6F9E61F-A3F5-5D9A-A58A-0D8014E1C9D0

#### Mesoveliinae

Douglas & Scott, 1867

B432CFB5-240C-5852-99B9-E511A14310DD

#### Mesovelia

Mulsant & Rey, 1852

97BD224A-B67B-5803-AD14-2E0B5B8FFD5E

#### Mesovelia amoena

Uhler, 1894

E6F78AA5-D18B-55EB-B665-8BAB7EF77ECA

##### Materials

- **Type status:** Other material. **Occurrence:** recordedBy: C. F. B. Floriano & T. Malacarne; individualCount: 1; sex: female; lifeStage: adult; occurrenceID: 4DE7C545-7F04-57A7-B73C-5D6724A89D86; **Taxon:** genus: Mesovelia; specificEpithet: amoena; taxonRank: species; scientificNameAuthorship: Uhler, 1894; **Location:** stateProvince: Santa Catarina; municipality: Indaial; locality: Parque Nacional da Serra do Itajaí, Pesque Pague do Wili, Riacho pequeno; decimalLatitude: -27.015; decimalLongitude: -49.164; **Identification:** identificationID: Mesovelia amoena; identifiedBy: C. F. B. Floriano; **Event:** samplingProtocol: "D" Net; year: 2017; month: IV; **Record Level:** institutionCode: CEIOC; basisOfRecord: PreservedSpecimen
- **Type status:** Other material. **Occurrence:** recordedBy: C. F. B. Floriano & T. Malacarne; individualCount: 6; sex: female; lifeStage: adult; occurrenceID: BEE63079-052E-5F0A-8DBC-0B9C6E95775B; **Taxon:** genus: Mesovelia; specificEpithet: amoena; taxonRank: species; scientificNameAuthorship: Uhler, 1894; **Location:** stateProvince: Santa Catarina; municipality: Blumenau; locality: Parque Nacional da Serra do Itajaí, Parque Natural Municipal Nascentes do Garcia, Lagoa Negra; decimalLatitude: -27.0532; decimalLongitude: -49.0878; **Identification:** identificationID: Mesovelia amoena; identifiedBy: C. F. B. Floriano; **Event:** samplingProtocol: "D" Net; year: 2017; month: IV; **Record Level:** institutionCode: CEIOC; basisOfRecord: PreservedSpecimen
- **Type status:** Other material. **Occurrence:** recordedBy: C. F. B. Floriano & T. Malacarne; individualCount: 2; sex: female; lifeStage: adult; occurrenceID: 97DB6BE0-83CA-5B80-88BA-0E45E65AAEC0; **Taxon:** genus: Mesovelia; specificEpithet: amoena; taxonRank: species; scientificNameAuthorship: Uhler, 1894; **Location:** stateProvince: Santa Catarina; municipality: Blumenau; locality: Parque Nacional da Serra do Itajaí, Parque Natural Municipal Nascentes do Garcia, Rio Garcia; decimalLatitude: -27.0538; decimalLongitude: -49.0863; **Identification:** identificationID: Mesovelia amoena; identifiedBy: I. R. S. Cordeiro; **Event:** samplingProtocol: "D" Net; year: 2017; month: IV; **Record Level:** institutionCode: CEIOC; basisOfRecord: PreservedSpecimen
- **Type status:** Other material. **Occurrence:** recordedBy: C. F. B. Floriano; individualCount: 1; sex: female; lifeStage: adult; occurrenceID: 5DD2D198-508A-55D1-BDAE-0CA24952D684; **Taxon:** genus: Mesovelia; specificEpithet: amoena; taxonRank: species; scientificNameAuthorship: Uhler, 1894; **Location:** stateProvince: Paraná; municipality: Tuneiras do Oeste; locality: Reserva Biológica das Perobas, Rio Mouro; decimalLatitude: -23.8850; decimalLongitude: -52.8072; **Identification:** identificationID: Mesovelia amoena; identifiedBy: C. F. B. Floriano; **Event:** samplingProtocol: "D" Net; year: 2024; month: IV; **Record Level:** institutionCode: CEIOC; basisOfRecord: PreservedSpecimen
- **Type status:** Other material. **Occurrence:** recordedBy: C. F. B. Floriano; individualCount: 7; sex: female; lifeStage: adult; occurrenceID: 1F524C1F-32F7-5D0D-9AAC-AD0453C35704; **Taxon:** genus: Mesovelia; specificEpithet: amoena; taxonRank: species; scientificNameAuthorship: Uhler, 1894; **Location:** stateProvince: Paraná; municipality: Tuneiras do Oeste; locality: Reserva Biológica das Perobas, Lagoa; decimalLatitude: -23.8894; decimalLongitude: -52.8144; **Identification:** identificationID: Mesovelia amoena; identifiedBy: C. F. B. Floriano; **Event:** samplingProtocol: "D" Net; year: 2024; month: IV; **Record Level:** institutionCode: CEIOC; basisOfRecord: PreservedSpecimen
- **Type status:** Other material. **Occurrence:** recordedBy: C. F. B. Floriano & T. Malacarne; individualCount: 1; sex: male; lifeStage: adult; occurrenceID: 91704A63-8143-59C0-94CB-477F5AAB8A98; **Taxon:** genus: Mesovelia; specificEpithet: amoena; taxonRank: species; scientificNameAuthorship: Uhler, 1894; **Location:** stateProvince: Santa Catarina; municipality: Blumenau; locality: Parque Nacional da Serra do Itajaí, riacho pequeno ao lado da estrada; **Identification:** identificationID: Mesovelia amoena; identifiedBy: C. F. B. Floriano; **Event:** samplingProtocol: "D" Net; year: 2017; month: IV; **Record Level:** institutionCode: CEIOC; basisOfRecord: PreservedSpecimen

##### Distribution

Hawaiian Islands, Galápagos Islands, Canada, United States, Mexico, Belize, Cuba, Jamaica, Dominican Republic, Puerto Rico, U.S. Virgin Islands, St. Eustatius, Martinique, Costa Rica, Panama, St. Vicent & the Grenadines, Curaçao, Bonaire, Trinidad & Tobago, Colombia, French Guiana, Brazil and Argentina.

##### Notes

**Figure 9.**
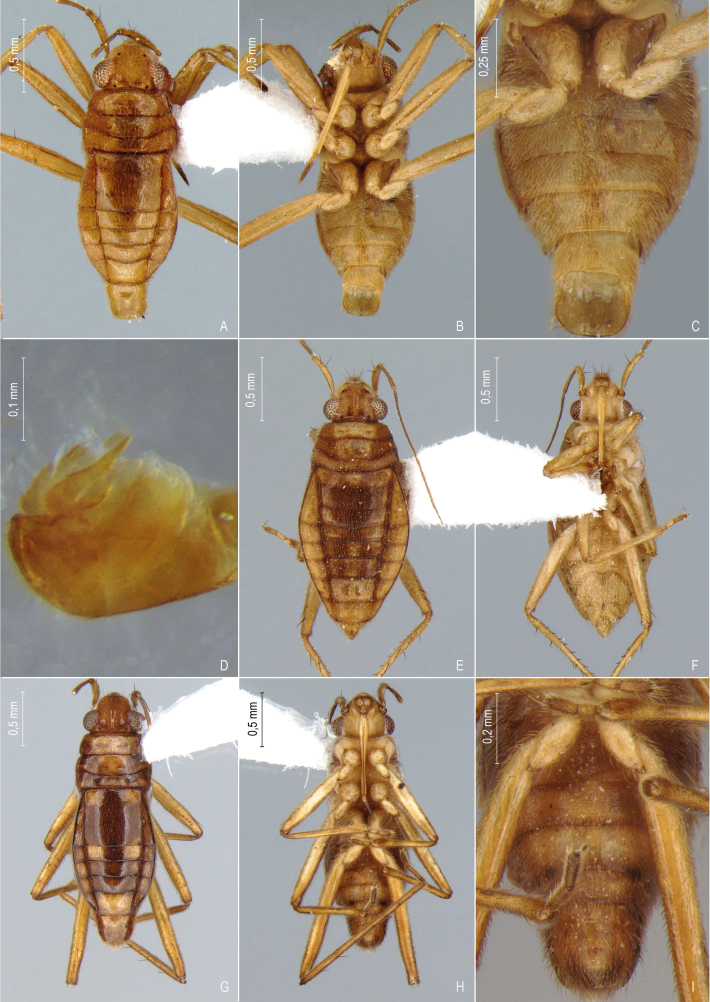
Mesoveliidae from the South Region of Brazil. **A**–**F.** *Mesovelia amoena*. **G**–**I.** *Mesovelia bila*, male. **A**–**D.** Male; **A** Habitus, dorsal view; **B** Habitus, ventral view; **C** Abdomen, ventral view; **D** Genital capsule, lateral view; **E, F.** Female; **E** Habitus, dorsal view; **F** Habitus, ventral view; **G** Habitus, dorsal view; **H** Habitus, ventral view; **I** Abdomen, ventral view.

First records from Santa Catarina (Fig. 7C, Fig. 9A-F). Previously reported from Paraná (Jaczewski 1928b). Males of this species are extremely rare in the Neotropics.

##### Diagnosis

Apterous form. Body length 2.00 mm; male mesofemur without row of spinules on posterior margin; male abdominal sterna VII and VIII without spines or clusters of black spinules; male abdominal segment VIII without lateral tubercles, posterior margin rounded dorsally, concave ventrally; paramere with margins converging distally, apex not bifid.

#### Mesovelia bila

Jaczewski, 1928

343D16AE-9D88-5C9F-8C56-C2E734F7C59B

##### Materials

- **Type status:** Other material. **Occurrence:** recordedBy: C. F. B. Floriano & T. Polizei; individualCount: 1; sex: male; lifeStage: adult; occurrenceID: D1733CFD-DE8E-51C3-997C-03459C33736C; **Taxon:** genus: Mesovelia; specificEpithet: bila; taxonRank: species; scientificNameAuthorship: Jaczewski, 1928; **Location:** stateProvince: Rio Grande do Sul; municipality: Cambará do Sul; locality: Parque Nacional de Aparados da Serra, Córrego Marcel; decimalLatitude: -29.17; decimalLongitude: -50.09; **Identification:** identificationID: Mesovelia bila; identifiedBy: C. F. B. Floriano; **Event:** samplingProtocol: "D" Net; year: 2016; month: IX; **Record Level:** institutionCode: CEIOC; basisOfRecord: PreservedSpecimen
- **Type status:** Other material. **Occurrence:** recordedBy: C. F. B. Floriano & T. Polizei; individualCount: 1; sex: male; lifeStage: adult; occurrenceID: A44F64AD-79E6-5178-83EF-DB3699BB48A0; **Taxon:** genus: Mesovelia; specificEpithet: bila; taxonRank: species; scientificNameAuthorship: Jaczewski, 1928; **Location:** stateProvince: Rio Grande do Sul; municipality: Cambará do Sul; locality: Higropétrico; decimalLatitude: -28.953; decimalLongitude: -50.056; **Identification:** identificationID: Mesovelia bila; identifiedBy: C. F. B. Floriano; **Event:** samplingProtocol: "D" Net; year: 2016; month: IX; **Record Level:** institutionCode: CEIOC; basisOfRecord: PreservedSpecimen
- **Type status:** Other material. **Occurrence:** recordedBy: C. F. B. Floriano & T. Polizei; individualCount: 1; sex: female; lifeStage: adult; occurrenceID: CC7289FA-E170-5FA4-A31F-944A8358164B; **Taxon:** genus: Mesovelia; specificEpithet: bila; taxonRank: species; scientificNameAuthorship: Jaczewski, 1928; **Location:** stateProvince: Rio Grande do Sul; municipality: Cambará do Sul; locality: Higropétrico; verbatimElevation: 880 m; decimalLatitude: -28.953; decimalLongitude: -50.056; **Identification:** identificationID: Mesovelia bila; identifiedBy: C. F. B. Floriano; **Event:** samplingProtocol: "D" Net; year: 2016; month: IX; **Record Level:** institutionCode: CEIOC; basisOfRecord: PreservedSpecimen

##### Distribution

Brazil and Argentina.

##### Notes

First records from Rio Grande do Sul (Fig. 7C, Fig. 9G-I). Previously reported from Paraná and Santa Catarina (Jaczewski 1928b, Floriano et al. 2017).

#### Veliidae

Brullé, 1836

280893CA-720F-5B63-9B38-08DE125379CD

#### Rhagoveliinae

China & Usinger, 1949

8FDBDA55-E608-5974-A782-C71A72D9FF0F

#### Rhagovelia

Mayr, 1865

67908266-FAB9-5570-B912-FC195A1964A5

#### Rhagovelia brunae

Magalhães & Moreira, 2016

1060468D-DBB6-5C0F-86D0-BFB62088B6FD

##### Materials

- **Type status:** Other material. **Occurrence:** recordedBy: C. F. B. Floriano; individualCount: 2; sex: male; lifeStage: adult; occurrenceID: 9483FEF7-282D-50F5-9495-299966019EF1; **Taxon:** genus: Rhagovelia; specificEpithet: brunae; taxonRank: species; scientificNameAuthorship: Magalhães & Moreira, 2016; **Location:** stateProvince: Paraná; municipality: Tuneiras do Oeste; locality: Reserva Biológica das Perobas, Rio Mouro; decimalLatitude: -23.8850; decimalLongitude: -52.8072; **Identification:** identificationID: Rhagovelia brunae; identifiedBy: C. F. B. Floriano; **Event:** samplingProtocol: "D" Net; year: 2024; month: IV; **Record Level:** institutionCode: CEIOC; basisOfRecord: PreservedSpecimen
- **Type status:** Other material. **Occurrence:** recordedBy: C. F. B. Floriano; individualCount: 9; sex: female; lifeStage: adult; occurrenceID: 77F4B545-29FE-5AB4-9B3B-EFAD23914216; **Taxon:** genus: Rhagovelia; specificEpithet: brunae; taxonRank: species; scientificNameAuthorship: Magalhães & Moreira, 2016; **Location:** stateProvince: Paraná; municipality: Tuneiras do Oeste; locality: Reserva Biológica das Perobas, Rio Mouro; decimalLatitude: -23.8850; decimalLongitude: -52.8072; **Identification:** identificationID: Rhagovelia brunae; identifiedBy: C. F. B. Floriano; **Event:** samplingProtocol: "D" Net; year: 2024; month: IV; **Record Level:** institutionCode: CEIOC; basisOfRecord: PreservedSpecimen

##### Distribution

Venezuela, French Guiana and Brazil.

##### Notes

**Figure 10.**
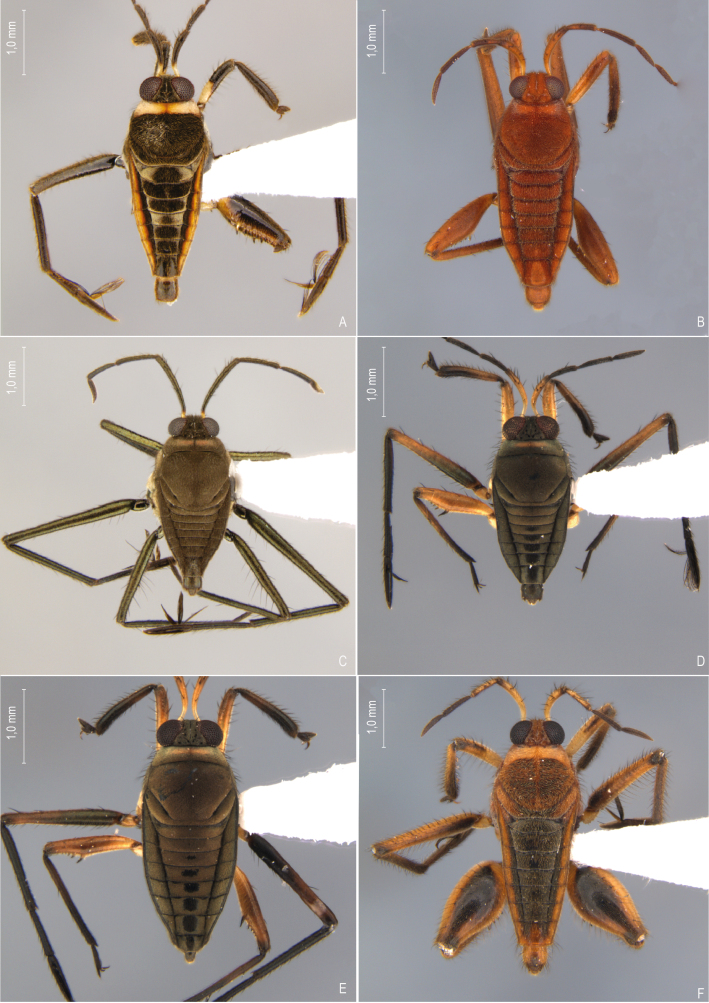
Veliidae from the South Region of Brazil, habitus, dorsal view. **A**–**D, F** Male. **E.** Female. **A** *Rhagovelia brunae*; **B** *Rhagovelia henryi*; **C** *Rhagovelia occulcata*; **D**, **E** *Rhagovelia ochroischion*; **F** *Rhagovelia robusta*.

**Figure 11.**
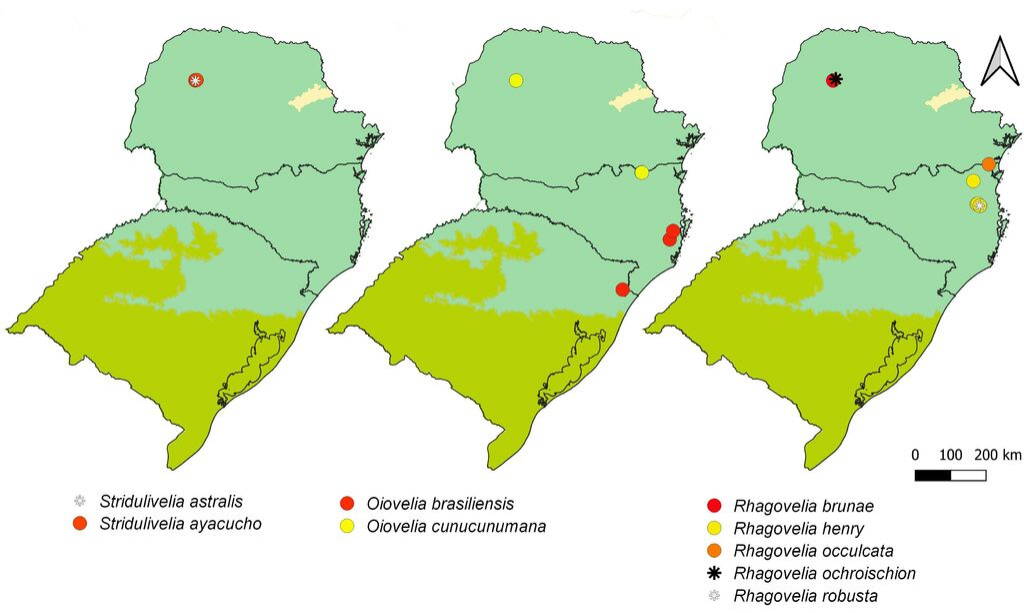
Maps showing new records of Veliidae (*Stridulivelia*, *Oiovelia* and *Rhagovelia*) from the South Region of Brazil.

First records from Paraná and the South Region of Brazil (Fig. 10A, Fig. 11C).

#### Rhagovelia henryi

Polhemus, 1997

51B851C0-64C7-57DB-8608-E7919BF508B5

##### Materials

- **Type status:** Other material. **Occurrence:** recordedBy: C. F. B. Floriano & T. Malacarne; individualCount: 18; sex: male; lifeStage: adult; occurrenceID: BD7A5913-8E9E-5753-9DE0-8BABC4D5B6C5; **Taxon:** genus: Rhagovelia; specificEpithet: henryi; taxonRank: species; scientificNameAuthorship: Polhemus, 1997; **Location:** stateProvince: Santa Catarina; municipality: Indaial; locality: Parque Nacional da Serra do Itajaí, Pesque Pague do Wili; decimalLatitude: -27.0158; decimalLongitude: -49.1660; **Identification:** identificationID: Rhagovelia henryi; identifiedBy: C. F. B. Floriano; **Event:** samplingProtocol: "D" Net; year: 2017; month: IV; **Record Level:** institutionCode: CEIOC; basisOfRecord: PreservedSpecimen
- **Type status:** Other material. **Occurrence:** recordedBy: C. F. B. Floriano & T. Malacarne; individualCount: 23; sex: female; lifeStage: adult; occurrenceID: C4E9DCD0-4182-5680-AE78-009B6F4FA962; **Taxon:** genus: Rhagovelia; specificEpithet: henryi; taxonRank: species; scientificNameAuthorship: Polhemus, 1997; **Location:** stateProvince: Santa Catarina; municipality: Indaial; locality: Parque Nacional da Serra do Itajaí, Pesque Pague do Wili; decimalLatitude: -27.0158; decimalLongitude: -49.1660; **Identification:** identificationID: Rhagovelia henryi; identifiedBy: C. F. B. Floriano; **Event:** samplingProtocol: "D" Net; year: 2017; month: IV; **Record Level:** institutionCode: CEIOC; basisOfRecord: PreservedSpecimen
- **Type status:** Other material. **Occurrence:** recordedBy: C. F. B. Floriano & T. Malacarne; individualCount: 27; sex: male; lifeStage: adult; occurrenceID: CB1974C0-BB28-5C9F-A496-C780A7F36103; **Taxon:** genus: Rhagovelia; specificEpithet: henryi; taxonRank: species; scientificNameAuthorship: Polhemus, 1997; **Location:** stateProvince: Santa Catarina; locality: Parque Nacional da Serra do Itajaí, KM 18; **Identification:** identificationID: Rhagovelia henryi; identifiedBy: C. F. B. Floriano; **Event:** samplingProtocol: "D" Net; year: 2017; month: IV; **Record Level:** institutionCode: CEIOC; basisOfRecord: PreservedSpecimen
- **Type status:** Other material. **Occurrence:** recordedBy: C. F. B. Floriano & T. Malacarne; individualCount: 35; sex: female; lifeStage: adult; occurrenceID: E57BD4FC-A224-59F9-894A-8DDA781DD282; **Taxon:** genus: Rhagovelia; specificEpithet: henryi; taxonRank: species; scientificNameAuthorship: Polhemus, 1997; **Location:** stateProvince: Santa Catarina; locality: Parque Nacional da Serra do Itajaí, KM 18; **Identification:** identificationID: Rhagovelia henryi; identifiedBy: C. F. B. Floriano; **Event:** samplingProtocol: "D" Net; year: 2017; month: IV; **Record Level:** institutionCode: CEIOC; basisOfRecord: PreservedSpecimen
- **Type status:** Other material. **Occurrence:** recordedBy: C. F. B. Floriano & T. Malacarne; individualCount: 6; sex: male; lifeStage: adult; occurrenceID: BC4623C1-F71A-51D2-B43B-E11DD14F6CD3; **Taxon:** genus: Rhagovelia; specificEpithet: henryi; taxonRank: species; scientificNameAuthorship: Polhemus, 1997; **Location:** stateProvince: Santa Catarina; municipality: Indaial; locality: Parque Nacional da Serra do Itajaí, Pesque Pague do Wili, rampa; decimalLatitude: -27.0155; decimalLongitude: -49.1655; **Identification:** identificationID: Rhagovelia henryi; identifiedBy: C. F. B. Floriano; **Event:** samplingProtocol: "D" Net; year: 2017; month: IV; **Record Level:** institutionCode: CEIOC; basisOfRecord: PreservedSpecimen
- **Type status:** Other material. **Occurrence:** recordedBy: C. F. B. Floriano & T. Malacarne; individualCount: 1; sex: female; lifeStage: adult; occurrenceID: 48B970C5-5698-5E98-B7E6-C6D113E96413; **Taxon:** genus: Rhagovelia; specificEpithet: henryi; taxonRank: species; scientificNameAuthorship: Polhemus, 1997; **Location:** stateProvince: Santa Catarina; municipality: Indaial; locality: Parque Nacional da Serra do Itajaí, Pesque Pague do Wili, rampa; decimalLatitude: -27.0155; decimalLongitude: -49.1655; **Identification:** identificationID: Rhagovelia henryi; identifiedBy: C. F. B. Floriano; **Event:** samplingProtocol: "D" Net; year: 2017; month: IV; **Record Level:** institutionCode: CEIOC; basisOfRecord: PreservedSpecimen
- **Type status:** Other material. **Occurrence:** recordedBy: C. F. B. Floriano & T. Malacarne; individualCount: 9; sex: male; lifeStage: adult; occurrenceID: 4FDD5F03-B9A0-5A9A-8352-81D3D222CDE2; **Taxon:** genus: Rhagovelia; specificEpithet: henryi; taxonRank: species; scientificNameAuthorship: Polhemus, 1997; **Location:** stateProvince: Santa Catarina; municipality: Blumenau; locality: Parque Nacional da Serra do Itajaí, riacho pequeno ao lado da estrada; **Identification:** identificationID: Rhagovelia henryi; identifiedBy: C. F. B. Floriano; **Event:** samplingProtocol: "D" Net; year: 2017; month: IV; **Record Level:** institutionCode: CEIOC; basisOfRecord: PreservedSpecimen
- **Type status:** Other material. **Occurrence:** recordedBy: C. F. B. Floriano & T. Malacarne; individualCount: 10; sex: female; lifeStage: adult; occurrenceID: 40FE7521-9A33-52D8-8749-03FDCC32DADB; **Taxon:** genus: Rhagovelia; specificEpithet: henryi; taxonRank: species; scientificNameAuthorship: Polhemus, 1997; **Location:** stateProvince: Santa Catarina; municipality: Blumenau; locality: Parque Nacional da Serra do Itajaí, riacho pequeno ao lado da estrada; **Identification:** identificationID: Rhagovelia henry; identifiedBy: C. F. B. Floriano; **Event:** samplingProtocol: "D" Net; year: 2017; month: IV; **Record Level:** institutionCode: CEIOC; basisOfRecord: PreservedSpecimen
- **Type status:** Other material. **Occurrence:** recordedBy: C. F. B. Floriano & T. Malacarne; individualCount: 41; sex: male; lifeStage: adult; occurrenceID: 79398D76-7A4C-58F6-B8A1-FB2E27B8782B; **Taxon:** genus: Rhagovelia; specificEpithet: henryi; taxonRank: species; scientificNameAuthorship: Polhemus, 1997; **Location:** stateProvince: Santa Catarina; municipality: Blumenau; locality: Parque Nacional da Serra do Itajaí, Parque Natural Municipal Nascentes do Garcia, Cachoeira da captação; decimalLatitude: -27.0545; decimalLongitude: -49.0838; **Identification:** identificationID: Rhagovelia henryi; identifiedBy: C. F. B. Floriano; **Event:** samplingProtocol: "D" Net; year: 2017; month: IV; **Record Level:** institutionCode: CEIOC; basisOfRecord: PreservedSpecimen
- **Type status:** Other material. **Occurrence:** recordedBy: C. F. B. Floriano & T. Malacarne; individualCount: 26; sex: female; lifeStage: adult; occurrenceID: 73F4393E-202E-54E3-B237-F9259C3C47BD; **Taxon:** genus: Rhagovelia; specificEpithet: henryi; taxonRank: species; scientificNameAuthorship: Polhemus, 1997; **Location:** stateProvince: Santa Catarina; municipality: Blumenau; locality: Parque Nacional da Serra do Itajaí, Parque Natural Municipal Nascentes do Garcia, Cachoeira da captação; decimalLatitude: -27.0545; decimalLongitude: -49.0838; **Identification:** identificationID: Rhagovelia henryi; identifiedBy: C. F. B. Floriano; **Event:** samplingProtocol: "D" Net; year: 2017; month: IV; **Record Level:** institutionCode: CEIOC; basisOfRecord: PreservedSpecimen
- **Type status:** Other material. **Occurrence:** recordedBy: C. F. B. Floriano & T. Malacarne; individualCount: 1; sex: male; lifeStage: adult; occurrenceID: 03A94530-A881-5DA5-BA17-5FAB3AFCC71B; **Taxon:** genus: Rhagovelia; specificEpithet: henryi; taxonRank: species; scientificNameAuthorship: Gould, 1931; **Location:** stateProvince: Santa Catarina; municipality: Blumenau; locality: Parque Nacional da Serra do Itajaí, Parque Natural Municipal Nascentes do Garcia, riacho pequeno; decimalLatitude: -27.052; decimalLongitude: -49.088; **Identification:** identificationID: Rhagovelia henryi; identifiedBy: C. F. B. Floriano; **Event:** samplingProtocol: "D" Net; year: 2017; month: IV; **Record Level:** institutionCode: CEIOC; basisOfRecord: PreservedSpecimen
- **Type status:** Other material. **Occurrence:** recordedBy: C. F. B. Floriano & T. Malacarne; individualCount: 1; sex: female; lifeStage: adult; occurrenceID: A6F90AC4-F42A-50C3-8256-72C0D33D3DB9; **Taxon:** genus: Rhagovelia; specificEpithet: henryi; taxonRank: species; scientificNameAuthorship: Gould, 1931; **Location:** stateProvince: Santa Catarina; municipality: Blumenau; locality: Parque Nacional da Serra do Itajaí, Parque Natural Municipal Nascentes do Garcia, riacho pequeno; decimalLatitude: -27.052; decimalLongitude: -49.088; **Identification:** identificationID: Rhagovelia henryi; identifiedBy: C. F. B. Floriano; **Event:** samplingProtocol: "D" Net; year: 2017; month: IV; **Record Level:** institutionCode: CEIOC; basisOfRecord: PreservedSpecimen
- **Type status:** Other material. **Occurrence:** recordedBy: C. F. B. Floriano & T. Malacarne; individualCount: 1; sex: male; lifeStage: adult; occurrenceID: 83C51755-29E0-5574-99FC-9E67E0E22D8C; **Taxon:** genus: Rhagovelia; specificEpithet: henryi; taxonRank: species; scientificNameAuthorship: Polhemus, 1997; **Location:** stateProvince: Santa Catarina; municipality: Corupá; locality: Rio Cachoeira; decimalLatitude: -26.425; decimalLongitude: -49.255; **Identification:** identificationID: Rhagovelia henryi; identifiedBy: C. F. B. Floriano; **Event:** samplingProtocol: "D" Net; year: 2017; month: IV; **Record Level:** institutionCode: CEIOC; basisOfRecord: PreservedSpecimen
- **Type status:** Other material. **Occurrence:** recordedBy: C. F. B. Floriano & T. Malacarne; individualCount: 1; sex: female; lifeStage: adult; occurrenceID: DFFBD604-F5AE-5E6E-AD97-B8AFC31D3568; **Taxon:** genus: Rhagovelia; specificEpithet: henryi; taxonRank: species; scientificNameAuthorship: Polhemus, 1997; **Location:** stateProvince: Santa Catarina; municipality: Corupá; locality: Rio Cachoeira; decimalLatitude: -26.425; decimalLongitude: -49.255; **Identification:** identificationID: Rhagovelia henry; identifiedBy: C. F. B. Floriano; **Event:** samplingProtocol: "D" Net; year: 2017; month: IV; **Record Level:** institutionCode: CEIOC; basisOfRecord: PreservedSpecimen
- **Type status:** Other material. **Occurrence:** recordedBy: T. Polizei & Família Roell; individualCount: 2; sex: male; lifeStage: adult; occurrenceID: 2919F308-E945-5DF0-AD4F-F36E0FB5A935; **Taxon:** genus: Rhagovelia; specificEpithet: henryi; taxonRank: species; scientificNameAuthorship: Polhemus, 1997; **Location:** stateProvince: Santa Catarina; municipality: Rio Negrinho; locality: Fazenda Evaristo Riacho Cachoeira do Índio; decimalLatitude: -26.3058; decimalLongitude: -49.5269; **Identification:** identificationID: Rhagovelia henry; identifiedBy: C. F. B. Floriano; **Event:** samplingProtocol: "D" Net; year: 2021; month: I; **Record Level:** institutionCode: CEIOC; basisOfRecord: PreservedSpecimen
- **Type status:** Other material. **Occurrence:** recordedBy: T. Polizei & Família Roell; individualCount: 3; sex: female; lifeStage: adult; occurrenceID: B7FA6E36-9816-5028-9D29-703241317B82; **Taxon:** genus: Rhagovelia; specificEpithet: henryi; taxonRank: species; scientificNameAuthorship: Polhemus, 1997; **Location:** stateProvince: Santa Catarina; municipality: Rio Negrinho; locality: Fazenda Evaristo Riacho Cachoeira do Índio; decimalLatitude: -26.3058; decimalLongitude: -49.5269; **Identification:** identificationID: Rhagovelia henry; identifiedBy: C. F. B. Floriano; **Event:** samplingProtocol: "D" Net; year: 2021; month: I; **Record Level:** institutionCode: CEIOC; basisOfRecord: PreservedSpecimen

##### Distribution

Brazil.

##### Notes

First records from Santa Catarina and the South Region of Brazil (Fig. 10B, Fig. 11C).

#### Rhagovelia lucida

Gould, 1931

FC1BB8BB-A088-5313-8126-A28327A4C936

##### Materials

- **Type status:** Other material. **Occurrence:** recordedBy: C. F. B. Floriano & T. Malacarne; individualCount: 2; sex: male; lifeStage: adult; occurrenceID: FC76CC9D-77A3-5980-8EA0-27E6AD9BDAB7; **Taxon:** genus: Rhagovelia; specificEpithet: lucida; taxonRank: species; scientificNameAuthorship: Gould, 1931; **Location:** stateProvince: Santa Catarina; municipality: Blumenau; locality: Parque Nacional da Serra do Itajaí, Parque Natural Municipal Nascentes do Garcia, Rio Garcia; decimalLatitude: -27.0538; decimalLongitude: -49.0863; **Identification:** identificationID: Rhagovelia lucida; identifiedBy: C. F. B. Floriano; **Event:** samplingProtocol: "D" Net; year: 2017; month: IV; **Record Level:** institutionCode: CEIOC; basisOfRecord: PreservedSpecimen
- **Type status:** Other material. **Occurrence:** recordedBy: C. F. B. Floriano & T. Malacarne; individualCount: 3; sex: male; lifeStage: adult; occurrenceID: B42A64A1-9D55-5BB6-820E-D9DE8BED511D; **Taxon:** genus: Rhagovelia; specificEpithet: lucida; taxonRank: species; scientificNameAuthorship: Gould, 1931; **Location:** stateProvince: Santa Catarina; locality: Parque Nacional da Serra do Itajaí, KM 18; **Identification:** identificationID: Rhagovelia lucida; identifiedBy: C. F. B. Floriano; **Event:** samplingProtocol: "D" Net; year: 2017; month: IV; **Record Level:** institutionCode: CEIOC; basisOfRecord: PreservedSpecimen
- **Type status:** Other material. **Occurrence:** recordedBy: C. F. B. Floriano & T. Malacarne; individualCount: 3; sex: male; lifeStage: adult; occurrenceID: EEC7ED80-97C0-5929-BFA4-BCAE14CB4166; **Taxon:** genus: Rhagovelia; specificEpithet: lucida; taxonRank: species; scientificNameAuthorship: Gould, 1931; **Location:** stateProvince: Santa Catarina; municipality: Indaial; locality: Parque Nacional da Serra do Itajaí, Pesque Pague do Wili; decimalLatitude: -27.0158; decimalLongitude: -49.1660; **Identification:** identificationID: Rhagovelia lucida; identifiedBy: C. F. B. Floriano; **Event:** samplingProtocol: "D" Net; year: 2017; month: IV; **Record Level:** institutionCode: CEIOC; basisOfRecord: PreservedSpecimen
- **Type status:** Other material. **Occurrence:** recordedBy: C. F. B. Floriano & T. Malacarne; individualCount: 33; sex: male; lifeStage: adult; occurrenceID: C93BF422-377E-545F-B302-F71EE9B5AA50; **Taxon:** genus: Rhagovelia; specificEpithet: lucida; taxonRank: species; scientificNameAuthorship: Gould, 1931; **Location:** stateProvince: Santa Catarina; municipality: Blumenau; locality: Parque Nacional da Serra do Itajaí, riacho pequeno ao lado da estrada; **Identification:** identificationID: Rhagovelia lucida; identifiedBy: C. F. B. Floriano; **Event:** samplingProtocol: "D" Net; year: 2017; month: IV; **Record Level:** institutionCode: CEIOC; basisOfRecord: PreservedSpecimen
- **Type status:** Other material. **Occurrence:** recordedBy: C. F. B. Floriano & T. Malacarne; individualCount: 4; sex: female; lifeStage: adult; occurrenceID: B57A9679-C1C5-5FD0-A913-29C9659FC7C4; **Taxon:** genus: Rhagovelia; specificEpithet: lucida; taxonRank: species; scientificNameAuthorship: Gould, 1931; **Location:** stateProvince: Santa Catarina; municipality: Blumenau; locality: Parque Nacional da Serra do Itajaí, riacho pequeno ao lado da estrada; **Identification:** identificationID: Rhagovelia lucida; identifiedBy: C. F. B. Floriano; **Event:** samplingProtocol: "D" Net; year: 2017; month: IV; **Record Level:** institutionCode: CEIOC; basisOfRecord: PreservedSpecimen
- **Type status:** Other material. **Occurrence:** recordedBy: C. F. B. Floriano & T. Malacarne; individualCount: 12; sex: male; lifeStage: adult; occurrenceID: EAF73B3E-4670-54A5-8F22-52612C377187; **Taxon:** genus: Rhagovelia; specificEpithet: lucida; taxonRank: species; scientificNameAuthorship: Gould, 1931; **Location:** stateProvince: Santa Catarina; municipality: Blumenau; locality: Parque Nacional da Serra do Itajaí, Parque Natural Municipal Nascentes do Garcia, riacho pequeno; decimalLatitude: -27.052; decimalLongitude: -49.088; **Identification:** identificationID: Rhagovelia lucida; identifiedBy: C. F. B. Floriano; **Event:** samplingProtocol: "D" Net; year: 2017; month: IV; **Record Level:** institutionCode: CEIOC; basisOfRecord: PreservedSpecimen
- **Type status:** Other material. **Occurrence:** recordedBy: C. F. B. Floriano & T. Malacarne; individualCount: 3; sex: female; lifeStage: adult; occurrenceID: CD05A20D-9287-53FE-8586-D46A4E5F267F; **Taxon:** genus: Rhagovelia; specificEpithet: lucida; taxonRank: species; scientificNameAuthorship: Gould, 1931; **Location:** stateProvince: Santa Catarina; municipality: Blumenau; locality: Parque Nacional da Serra do Itajaí, Parque Natural Municipal Nascentes do Garcia, riacho pequeno; decimalLatitude: -27.052; decimalLongitude: -49.088; **Identification:** identificationID: Rhagovelia lucida; identifiedBy: C. F. B. Floriano; **Event:** samplingProtocol: "D" Net; year: 2017; month: IV; **Record Level:** institutionCode: CEIOC; basisOfRecord: PreservedSpecimen
- **Type status:** Other material. **Occurrence:** recordedBy: C. F. B. Floriano & T. Polizei; individualCount: 66; sex: male; lifeStage: adult; occurrenceID: 47D6277A-7FD7-5C40-8303-335B72A4368D; **Taxon:** genus: Rhagovelia; specificEpithet: lucida; taxonRank: species; scientificNameAuthorship: Gould, 1931; **Location:** stateProvince: Santa Catarina; municipality: Rancho Queimado; locality: Rio das Antas; decimalLatitude: -27.673; decimalLongitude: -49.012; **Identification:** identificationID: Rhagovelia lucida; identifiedBy: C. F. B. Floriano; **Event:** samplingProtocol: "D" Net; year: 2016; month: IX; **Record Level:** institutionCode: CEIOC; basisOfRecord: PreservedSpecimen
- **Type status:** Other material. **Occurrence:** recordedBy: C. F. B. Floriano & T. Polizei; individualCount: 37; sex: female; lifeStage: adult; occurrenceID: E4246160-804E-54AE-8884-78747A5793D0; **Taxon:** genus: Rhagovelia; specificEpithet: lucida; taxonRank: species; scientificNameAuthorship: Gould, 1931; **Location:** stateProvince: Santa Catarina; municipality: Rancho Queimado; locality: Rio das Antas; decimalLatitude: -27.673; decimalLongitude: -49.012; **Identification:** identificationID: Rhagovelia lucida; identifiedBy: C. F. B. Floriano; **Event:** samplingProtocol: "D" Net; year: 2016; month: IX; **Record Level:** institutionCode: CEIOC; basisOfRecord: PreservedSpecimen
- **Type status:** Other material. **Occurrence:** recordedBy: C. F. B. Floriano & T. Polizei; individualCount: 2; sex: male; lifeStage: adult; occurrenceID: 86D0B84D-1D51-51FE-90D5-A31BE6307125; **Taxon:** genus: Rhagovelia; specificEpithet: lucida; taxonRank: species; scientificNameAuthorship: Gould, 1931; **Location:** stateProvince: Santa Catarina; municipality: Rancho Queimado; locality: Rio Cubatão; decimalLatitude: -27.673; decimalLongitude: -49.012; **Identification:** identificationID: Rhagovelia lucida; identifiedBy: C. F. B. Floriano; **Event:** samplingProtocol: "D" Net; year: 2016; month: IX; **Record Level:** institutionCode: CEIOC; basisOfRecord: PreservedSpecimen
- **Type status:** Other material. **Occurrence:** recordedBy: C. F. B. Floriano & T. Polizei; individualCount: 1; sex: female; lifeStage: adult; occurrenceID: EAA0409A-DD52-5B29-B846-1FDFFB6F95CF; **Taxon:** genus: Rhagovelia; specificEpithet: lucida; taxonRank: species; scientificNameAuthorship: Gould, 1931; **Location:** stateProvince: Santa Catarina; municipality: Rancho Queimado; locality: Rio Cubatão; decimalLatitude: -27.673; decimalLongitude: -49.012; **Identification:** identificationID: Rhagovelia lucida; identifiedBy: C. F. B. Floriano; **Event:** samplingProtocol: "D" Net; year: 2016; month: IX; **Record Level:** institutionCode: CEIOC; basisOfRecord: PreservedSpecimen
- **Type status:** Other material. **Occurrence:** recordedBy: C. F. B. Floriano & T. Polizei; individualCount: 4; sex: female; lifeStage: adult; occurrenceID: BEB6B9FF-348C-5A1D-9546-2AFE2B72B15F; **Taxon:** genus: Rhagovelia; specificEpithet: lucida; taxonRank: species; scientificNameAuthorship: Gould, 1931; **Location:** stateProvince: Santa Catarina; municipality: São Bonifácio; locality: Trilha do Cedro; decimalLatitude: -27.89; decimalLongitude: -48.92; **Identification:** identificationID: Rhagovelia lucida; identifiedBy: C. F. B. Floriano; **Event:** samplingProtocol: "D" Net; year: 2016; month: IX; **Record Level:** institutionCode: CEIOC; basisOfRecord: PreservedSpecimen
- **Type status:** Other material. **Occurrence:** recordedBy: C. F. B. Floriano & T. Polizei; individualCount: 3; sex: male; lifeStage: adult; occurrenceID: 1BADAA05-B62C-577A-B9ED-81D9185BDB06; **Taxon:** genus: Rhagovelia; specificEpithet: lucida; taxonRank: species; scientificNameAuthorship: Gould, 1931; **Location:** stateProvince: Santa Catarina; municipality: São Bonifácio; locality: Rio Atafora; decimalLatitude: -27.89; decimalLongitude: -48.92; **Identification:** identificationID: Rhagovelia lucida; identifiedBy: C. F. B. Floriano; **Event:** samplingProtocol: "D" Net; year: 2016; month: IX; **Record Level:** institutionCode: CEIOC; basisOfRecord: PreservedSpecimen
- **Type status:** Other material. **Occurrence:** recordedBy: C. F. B. Floriano & T. Polizei; individualCount: 4; sex: female; lifeStage: adult; occurrenceID: 95B7D188-E237-5907-BB62-CC4E8E84C118; **Taxon:** genus: Rhagovelia; specificEpithet: lucida; taxonRank: species; scientificNameAuthorship: Gould, 1931; **Location:** stateProvince: Santa Catarina; municipality: São Bonifácio; locality: Rio Atafora; decimalLatitude: -27.89; decimalLongitude: -48.92; **Identification:** identificationID: Rhagovelia lucida; identifiedBy: C. F. B. Floriano; **Event:** samplingProtocol: "D" Net; year: 2016; month: IX; **Record Level:** institutionCode: CEIOC; basisOfRecord: PreservedSpecimen
- **Type status:** Other material. **Occurrence:** recordedBy: C. F. B. Floriano & T. Polizei; individualCount: 5; sex: male; lifeStage: adult; occurrenceID: 4EA0E2FD-9FFF-5BD6-AE2F-51C61BEFCAD9; **Taxon:** genus: Rhagovelia; specificEpithet: lucida; taxonRank: species; scientificNameAuthorship: Gould, 1931; **Location:** stateProvince: Santa Catarina; municipality: São Bonifácio; locality: Rio Ferro; decimalLatitude: -27.89; decimalLongitude: -48.92; **Identification:** identificationID: Rhagovelia lucida; identifiedBy: C. F. B. Floriano; **Event:** samplingProtocol: "D" Net; year: 2016; month: IX; **Record Level:** institutionCode: CEIOC; basisOfRecord: PreservedSpecimen
- **Type status:** Other material. **Occurrence:** recordedBy: C. F. B. Floriano & T. Polizei; individualCount: 6; sex: female; lifeStage: adult; occurrenceID: 786D6CEF-D818-58F1-9B74-D5076DE5576C; **Taxon:** genus: Rhagovelia; specificEpithet: lucida; taxonRank: species; scientificNameAuthorship: Gould, 1931; **Location:** stateProvince: Santa Catarina; municipality: São Bonifácio; locality: Rio Ferro; decimalLatitude: -27.89; decimalLongitude: -48.92; **Identification:** identificationID: Rhagovelia lucida; identifiedBy: C. F. B. Floriano; **Event:** samplingProtocol: "D" Net; year: 2016; month: IX; **Record Level:** institutionCode: CEIOC; basisOfRecord: PreservedSpecimen
- **Type status:** Other material. **Occurrence:** recordedBy: C. F. B. Floriano & T. Polizei; individualCount: 7; sex: male; lifeStage: adult; occurrenceID: 3CE4D6F9-8ABF-55E1-9C44-38B2EED7BEBB; **Taxon:** genus: Rhagovelia; specificEpithet: lucida; taxonRank: species; scientificNameAuthorship: Gould, 1931; **Location:** stateProvince: Santa Catarina; municipality: São Bonifácio; locality: Rio Moll; decimalLatitude: -27.89; decimalLongitude: -48.92; **Identification:** identificationID: Rhagovelia lucida; identifiedBy: C. F. B. Floriano; **Event:** samplingProtocol: "D" Net; year: 2016; month: IX; **Record Level:** institutionCode: CEIOC; basisOfRecord: PreservedSpecimen
- **Type status:** Other material. **Occurrence:** recordedBy: C. F. B. Floriano & T. Polizei; individualCount: 13; sex: male; lifeStage: adult; occurrenceID: E7666F41-FDC5-5C69-AFD5-962F5DC51940; **Taxon:** genus: Rhagovelia; specificEpithet: lucida; taxonRank: species; scientificNameAuthorship: Gould, 1931; **Location:** stateProvince: Santa Catarina; municipality: São Bonifácio; locality: Rio Moll; decimalLatitude: -27.89; decimalLongitude: -48.92; **Identification:** identificationID: Rhagovelia lucida; identifiedBy: C. F. B. Floriano; **Event:** samplingProtocol: "D" Net; year: 2016; month: IX; **Record Level:** institutionCode: CEIOC; basisOfRecord: PreservedSpecimen
- **Type status:** Other material. **Occurrence:** recordedBy: C. F. B. Floriano & T. Polizei; individualCount: 9; sex: male; lifeStage: adult; occurrenceID: 00151E5F-05F4-5B46-A936-8BDF8CA3DD7D; **Taxon:** genus: Rhagovelia; specificEpithet: lucida; taxonRank: species; scientificNameAuthorship: Gould, 1931; **Location:** stateProvince: Santa Catarina; municipality: São Bonifácio; locality: Riacho Dona Terezinha, Montanha; decimalLatitude: -27.9088; decimalLongitude: -48.9126; **Identification:** identificationID: Rhagovelia lucida; identifiedBy: C. F. B. Floriano; **Event:** samplingProtocol: "D" Net; year: 2016; month: IX; **Record Level:** institutionCode: CEIOC; basisOfRecord: PreservedSpecimen
- **Type status:** Other material. **Occurrence:** recordedBy: C. F. B. Floriano & T. Polizei; individualCount: 15; sex: male; lifeStage: adult; occurrenceID: 94261684-61E4-5A5E-8C1B-A02427963F7C; **Taxon:** genus: Rhagovelia; specificEpithet: lucida; taxonRank: species; scientificNameAuthorship: Gould, 1931; **Location:** stateProvince: Santa Catarina; municipality: São Bonifácio; locality: Riacho Dona Terezinha, Montanha; decimalLatitude: -27.9088; decimalLongitude: -48.9126; **Identification:** identificationID: Rhagovelia lucida; identifiedBy: C. F. B. Floriano; **Event:** samplingProtocol: "D" Net; year: 2016; month: IX; **Record Level:** institutionCode: CEIOC; basisOfRecord: PreservedSpecimen
- **Type status:** Other material. **Occurrence:** recordedBy: C. F. B. Floriano & T. Polizei; individualCount: 2; sex: male; lifeStage: adult; occurrenceID: 4FB2846C-4C00-567E-8AE2-FB8D771246B4; **Taxon:** genus: Rhagovelia; specificEpithet: lucida; taxonRank: species; scientificNameAuthorship: Gould, 1931; **Location:** stateProvince: Santa Catarina; municipality: Águas Mornas; locality: Rio Braço da Forquilha; decimalLatitude: -27.694; decimalLongitude: -48.825; **Identification:** identificationID: Rhagovelia lucida; identifiedBy: C. F. B. Floriano; **Event:** samplingProtocol: "D" Net; year: 2016; month: IX; **Record Level:** institutionCode: CEIOC; basisOfRecord: PreservedSpecimen
- **Type status:** Other material. **Occurrence:** recordedBy: C. F. B. Floriano & T. Polizei; individualCount: 10; sex: male; lifeStage: adult; occurrenceID: F43A0B33-21C3-5FC6-9B64-D9C8DC08E247; **Taxon:** genus: Rhagovelia; specificEpithet: lucida; taxonRank: species; scientificNameAuthorship: Gould, 1931; **Location:** stateProvince: Santa Catarina; municipality: Corupá; locality: Cachoeira da antiga usina; decimalLatitude: -26.389; decimalLongitude: -49.222; **Identification:** identificationID: Rhagovelia lucida; identifiedBy: C. F. B. Floriano; **Event:** samplingProtocol: "D" Net; year: 2017; month: IV; **Record Level:** institutionCode: CEIOC; basisOfRecord: PreservedSpecimen
- **Type status:** Other material. **Occurrence:** recordedBy: C. F. B. Floriano & T. Polizei; individualCount: 2; sex: female; lifeStage: adult; occurrenceID: 876420CF-EE6D-5A07-9F53-D00ABE72DA82; **Taxon:** genus: Rhagovelia; specificEpithet: lucida; taxonRank: species; scientificNameAuthorship: Gould, 1931; **Location:** stateProvince: Santa Catarina; municipality: Corupá; locality: Cachoeira da antiga usina; decimalLatitude: -26.389; decimalLongitude: -49.222; **Identification:** identificationID: Rhagovelia lucida; identifiedBy: C. F. B. Floriano; **Event:** samplingProtocol: "D" Net; year: 2017; month: IV; **Record Level:** institutionCode: CEIOC; basisOfRecord: PreservedSpecimen
- **Type status:** Other material. **Occurrence:** recordedBy: T. Roell & T. Polizei; individualCount: 12; sex: male; lifeStage: adult; occurrenceID: D20CEDB5-5431-563F-A37F-BD3B5D0DB54E; **Taxon:** genus: Rhagovelia; specificEpithet: lucida; taxonRank: species; scientificNameAuthorship: Gould, 1931; **Location:** stateProvince: Santa Catarina; municipality: São Bento do Sul; locality: Serra do Corupá, Rio Estrada Galinhas; decimalLatitude: -26.3494; decimalLongitude: -49.3317; **Identification:** identificationID: Rhagovelia lucida; identifiedBy: C. F. B. Floriano; **Event:** samplingProtocol: "D" Net; year: 2020; month: IV; **Record Level:** institutionCode: CEIOC; basisOfRecord: PreservedSpecimen
- **Type status:** Other material. **Occurrence:** recordedBy: T. Polizei & B. Araujo; individualCount: 1; sex: male; lifeStage: adult; occurrenceID: 01D5CF19-9663-5463-901B-EA9757BB61AA; **Taxon:** genus: Rhagovelia; specificEpithet: lucida; taxonRank: species; scientificNameAuthorship: Gould, 1931; **Location:** stateProvince: Paraná; municipality: Morretes; locality: Afluente do rio Brumado; decimalLatitude: -25.500; decimalLongitude: -48.866; **Identification:** identificationID: Rhagovelia lucida; identifiedBy: C. F. B. Floriano; **Event:** samplingProtocol: "D" Net; year: 2020; month: IV; **Record Level:** institutionCode: CEIOC; basisOfRecord: PreservedSpecimen
- **Type status:** Other material. **Occurrence:** recordedBy: T. Polizei & B. Araujo; individualCount: 1; sex: female; lifeStage: adult; occurrenceID: 3B1107CE-A2A5-57E0-97A0-DB7C1B719A8E; **Taxon:** genus: Rhagovelia; specificEpithet: lucida; taxonRank: species; scientificNameAuthorship: Gould, 1931; **Location:** stateProvince: Paraná; municipality: Morretes; locality: Afluente do rio Brumado; decimalLatitude: -25.500; decimalLongitude: -48.866; **Identification:** identificationID: Rhagovelia lucida; identifiedBy: C. F. B. Floriano; **Event:** samplingProtocol: "D" Net; year: 2020; month: IV; **Record Level:** institutionCode: CEIOC; basisOfRecord: PreservedSpecimen
- **Type status:** Other material. **Occurrence:** recordedBy: T. Polizei & B. Araujo; individualCount: 1; sex: male; lifeStage: adult; occurrenceID: C47B3897-C620-50B6-A512-35943E767C21; **Taxon:** genus: Rhagovelia; specificEpithet: lucida; taxonRank: species; scientificNameAuthorship: Gould, 1931; **Location:** stateProvince: Paraná; municipality: Morretes; locality: Serra da Graciosa, Rio Curva da Ferradura; decimalLatitude: -25.3594; decimalLongitude: -48.8853; **Identification:** identificationID: Rhagovelia lucida; identifiedBy: C. F. B. Floriano; **Event:** samplingProtocol: "D" Net; year: 2020; month: IV; **Record Level:** institutionCode: CEIOC; basisOfRecord: PreservedSpecimen
- **Type status:** Other material. **Occurrence:** recordedBy: T. Polizei & B. Araujo; individualCount: 1; sex: female; lifeStage: adult; occurrenceID: 235A2DC7-8E41-5055-A19D-54DECAA99786; **Taxon:** genus: Rhagovelia; specificEpithet: lucida; taxonRank: species; scientificNameAuthorship: Gould, 1931; **Location:** stateProvince: Paraná; municipality: Morretes; locality: Serra da Graciosa, Rio Curva da Ferradura; decimalLatitude: -25.3594; decimalLongitude: -48.8853; **Identification:** identificationID: Rhagovelia lucida; identifiedBy: C. F. B. Floriano; **Event:** samplingProtocol: "D" Net; year: 2020; month: IV; **Record Level:** institutionCode: CEIOC; basisOfRecord: PreservedSpecimen
- **Type status:** Other material. **Occurrence:** recordedBy: T. Polizei & B. Araujo; individualCount: 3; sex: male; lifeStage: adult; occurrenceID: 416D01D5-DFA2-5171-B0C8-3FD8E4897A11; **Taxon:** genus: Rhagovelia; specificEpithet: lucida; taxonRank: species; scientificNameAuthorship: Gould, 1931; **Location:** stateProvince: Paraná; municipality: Morretes; locality: Rio Burrado; decimalLatitude: -25.5069; decimalLongitude: -48.8853; **Identification:** identificationID: Rhagovelia lucida; identifiedBy: C. F. B. Floriano; **Event:** samplingProtocol: "D" Net; year: 2020; month: IV; **Record Level:** institutionCode: CEIOC; basisOfRecord: PreservedSpecimen
- **Type status:** Other material. **Occurrence:** recordedBy: T. Polizei & B. Araujo; individualCount: 1; sex: female; lifeStage: adult; occurrenceID: BB767742-B25E-57D3-8B78-0129D3629A5B; **Taxon:** genus: Rhagovelia; specificEpithet: lucida; taxonRank: species; scientificNameAuthorship: Gould, 1931; **Location:** stateProvince: Paraná; municipality: Morretes; locality: Rio Burrado; decimalLatitude: -25.5069; decimalLongitude: -48.8853; **Identification:** identificationID: Rhagovelia lucida; identifiedBy: C. F. B. Floriano; **Event:** samplingProtocol: "D" Net; year: 2020; month: IV; **Record Level:** institutionCode: CEIOC; basisOfRecord: PreservedSpecimen
- **Type status:** Other material. **Occurrence:** recordedBy: C. F. B. Floriano & T. Malacarne; individualCount: 1; sex: male; lifeStage: adult; occurrenceID: 0EF8FDA6-56D5-518A-B5A1-D686871C6CC1; **Taxon:** genus: Rhagovelia; specificEpithet: lucida; taxonRank: species; scientificNameAuthorship: Gould, 1931; **Location:** stateProvince: Paraná; municipality: Balsa Nova; locality: São Luiz do Purumã, Rio das Pombas; decimalLatitude: -25.44024; decimalLongitude: -49.74974; **Identification:** identificationID: Rhagovelia lucida; identifiedBy: C. F. B. Floriano; **Event:** samplingProtocol: "D" Net; year: 2017; month: IV; **Record Level:** institutionCode: CEIOC; basisOfRecord: PreservedSpecimen
- **Type status:** Other material. **Occurrence:** recordedBy: T. Polizei & B. Araujo; individualCount: 9; sex: male; lifeStage: adult; occurrenceID: 5FBDCDF5-3E12-52F5-ACB2-09FB95EF80EB; **Taxon:** genus: Rhagovelia; specificEpithet: lucida; taxonRank: species; scientificNameAuthorship: Gould, 1931; **Location:** stateProvince: Paraná; municipality: Morretes; locality: Riacho Iapó; **Identification:** identificationID: Rhagovelia lucida; identifiedBy: C. F. B. Floriano; **Event:** samplingProtocol: "D" Net; year: 2020; month: IV; **Record Level:** institutionCode: CEIOC; basisOfRecord: PreservedSpecimen
- **Type status:** Other material. **Occurrence:** recordedBy: T. Polizei & B. Araujo; individualCount: 3; sex: female; lifeStage: adult; occurrenceID: 2424ABB6-4EF7-5E9B-A22E-CAF69CCC8CFE; **Taxon:** genus: Rhagovelia; specificEpithet: lucida; taxonRank: species; scientificNameAuthorship: Gould, 1931; **Location:** stateProvince: Paraná; municipality: Morretes; locality: Riacho Iapó; **Identification:** identificationID: Rhagovelia lucida; identifiedBy: C. F. B. Floriano; **Event:** samplingProtocol: "D" Net; year: 2020; month: IV; **Record Level:** institutionCode: CEIOC; basisOfRecord: PreservedSpecimen
- **Type status:** Other material. **Occurrence:** recordedBy: T. Polizei & B. Araujo; individualCount: 4; sex: female; lifeStage: adult; occurrenceID: 744CEEE5-724C-53EE-B319-DC7F7044D667; **Taxon:** genus: Rhagovelia; specificEpithet: lucida; taxonRank: species; scientificNameAuthorship: Gould, 1931; **Location:** stateProvince: Paraná; municipality: Morretes; locality: Rio Mãe Catira; decimalLatitude: -25.350000; decimalLongitude: -48.866667; **Identification:** identificationID: Rhagovelia lucida; identifiedBy: C. F. B. Floriano; **Event:** samplingProtocol: "D" Net; year: 2020; month: IV; **Record Level:** institutionCode: CEIOC; basisOfRecord: PreservedSpecimen
- **Type status:** Other material. **Occurrence:** recordedBy: T. Polizei & B. Araujo; individualCount: 5; sex: male; lifeStage: adult; occurrenceID: E664605E-4A81-5369-9929-7CCD8D7D948B; **Taxon:** genus: Rhagovelia; specificEpithet: lucida; taxonRank: species; scientificNameAuthorship: Gould, 1931; **Location:** stateProvince: Paraná; municipality: Morretes; locality: Rio Mãe Catira; decimalLatitude: -25.350000; decimalLongitude: -48.866667; **Identification:** identificationID: Rhagovelia lucida; identifiedBy: C. F. B. Floriano; **Event:** samplingProtocol: "D" Net; year: 2020; month: IV; **Record Level:** institutionCode: CEIOC; basisOfRecord: PreservedSpecimen
- **Type status:** Other material. **Occurrence:** recordedBy: C. F. B. Floriano; individualCount: 100; sex: male; lifeStage: adult; occurrenceID: 37A4BA7C-051A-56B8-8427-5B54481D24A0; **Taxon:** genus: Rhagovelia; specificEpithet: lucida; taxonRank: species; scientificNameAuthorship: Gould, 1931; **Location:** stateProvince: Paraná; municipality: Quatro Barras; locality: Rio Taquari; decimalLatitude: -25.3464; decimalLongitude: -48.9319; **Identification:** identificationID: Rhagovelia lucida; identifiedBy: C. F. B. Floriano; **Event:** samplingProtocol: "D" Net; year: 2020; month: IV; **Record Level:** institutionCode: CEIOC; basisOfRecord: PreservedSpecimen
- **Type status:** Other material. **Occurrence:** recordedBy: C. F. B. Floriano; individualCount: 93; sex: female; lifeStage: adult; occurrenceID: D2290489-BEF6-5D50-AEBC-129FA6677187; **Taxon:** genus: Rhagovelia; specificEpithet: lucida; taxonRank: species; scientificNameAuthorship: Gould, 1931; **Location:** stateProvince: Paraná; municipality: Quatro Barras; locality: Rio Taquari; decimalLatitude: -25.3464; decimalLongitude: -48.9319; **Identification:** identificationID: Rhagovelia lucida; identifiedBy: C. F. B. Floriano; **Event:** samplingProtocol: "D" Net; year: 2020; month: IV; **Record Level:** institutionCode: CEIOC; basisOfRecord: PreservedSpecimen

##### Distribution

Brazil and Argentina.

##### Notes

Previously reported from Paraná, Santa Catarina and Rio Grande do Sul (Bacon 1956, Polhemus 1997, Moreira and Campos 2012, Cordeiro and Moreira 2015).

#### Rhagovelia occulcata

Drake, 1959

F1AF0946-664A-542E-BA01-4B88923AE06F

##### Materials

- **Type status:** Other material. **Occurrence:** recordedBy: R. P. R. Canejo, J. M. S. Rodrigues, M. S. L. Alexandre, L. P. Pereira; individualCount: 26; sex: male; lifeStage: adult; occurrenceID: 19AD677B-A98E-5522-B8A9-B3F09334F7B0; **Taxon:** genus: Rhagovelia; specificEpithet: occulcata; taxonRank: species; scientificNameAuthorship: Drake, 1959; **Location:** stateProvince: Santa Catarina; municipality: Garuva; locality: Rio São João; decimalLatitude: -26.0054; decimalLongitude: -48.8567; **Identification:** identificationID: Rhagovelia occulcata; identifiedBy: N. O. Paiva; **Event:** samplingProtocol: "D" Net; year: 2023; month: X; **Record Level:** institutionCode: CEIOC; basisOfRecord: PreservedSpecimen
- **Type status:** Other material. **Occurrence:** recordedBy: R. P. R. Canejo, J. M. S. Rodrigues, M. S. L. Alexandre, L. P. Pereira; individualCount: 13; sex: female; lifeStage: adult; occurrenceID: 9417E27A-82CB-570C-B97C-B1F2A1C0E5BF; **Taxon:** genus: Rhagovelia; specificEpithet: occulcata; taxonRank: species; scientificNameAuthorship: Drake, 1959; **Location:** stateProvince: Santa Catarina; municipality: Garuva; locality: Rio São João; decimalLatitude: -26.0054; decimalLongitude: -48.8567; **Identification:** identificationID: Rhagovelia occulcata; identifiedBy: N. O. Paiva; **Event:** samplingProtocol: "D" Net; year: 2023; month: X; **Record Level:** institutionCode: CEIOC; basisOfRecord: PreservedSpecimen

##### Distribution

Brazil.

##### Notes

First records from Santa Catarina (Fig. 10C, Fig. 11C). Described from Paraná (Drake 1959).

#### Rhagovelia ochroischion

Nieser & Melo, 1997

5F316D89-E904-532D-AE33-A12F23C8CD47

##### Materials

- **Type status:** Other material. **Occurrence:** recordedBy: C. F. B. Floriano; individualCount: 5; sex: male; lifeStage: adult; occurrenceID: EDEA14F9-0D26-5232-8A4C-22561F2A6E3F; **Taxon:** genus: Rhagovelia; specificEpithet: ochroischion; taxonRank: species; scientificNameAuthorship: Nieser & Melo, 1997; **Location:** stateProvince: Paraná; municipality: Cianorte; locality: Reserva Biológica das Perobas, Córrego Adelaide; decimalLatitude: -23.8472; decimalLongitude: -52.7389; **Identification:** identificationID: Rhagovelia ochroischion; identifiedBy: N. O. Paiva; **Event:** samplingProtocol: "D" Net; year: 2024; month: IV; **Record Level:** institutionCode: CEIOC; basisOfRecord: PreservedSpecimen
- **Type status:** Other material. **Occurrence:** recordedBy: C. F. B. Floriano; individualCount: 12; sex: female; lifeStage: adult; occurrenceID: 3F8C20F5-90FA-5960-B3A4-7C57CB5F26AD; **Taxon:** genus: Rhagovelia; specificEpithet: ochroischion; taxonRank: species; scientificNameAuthorship: Nieser & Melo, 1997; **Location:** stateProvince: Paraná; municipality: Cianorte; locality: Reserva Biológica das Perobas, Córrego Adelaide; decimalLatitude: -23.8472; decimalLongitude: -52.7389; **Identification:** identificationID: Rhagovelia ochroischion; identifiedBy: N. O. Paiva; **Event:** samplingProtocol: "D" Net; year: 2024; month: IV; **Record Level:** institutionCode: CEIOC; basisOfRecord: PreservedSpecimen

##### Distribution

Brazil.

##### Notes

Specimens from Paraná show variations in structure and colouration when compared to the original description. In the material above, the apical portion of the middle femur and most of the fore femur dorsally are yellowish. Additionally, both males and females bear a row of five to seven spines on the hind femur. These are the first records from Paraná and the South Region of Brazil (Fig. 10D-E, Fig. 11C).

#### Rhagovelia robusta

Gould, 1931

0780FE41-D2D0-51E7-960E-3C22C9310708

##### Materials

- **Type status:** Other material. **Occurrence:** recordedBy: C. F. B. Floriano; individualCount: 1; sex: male; lifeStage: adult; occurrenceID: 433E47B7-2710-5904-A394-768B56266F8D; **Taxon:** genus: Rhagovelia; specificEpithet: robusta; taxonRank: species; scientificNameAuthorship: Gould, 1931; **Location:** stateProvince: Paraná; municipality: Tuneiras do Oeste; locality: Reserva Biológica das Perobas, brejo na trilha; decimalLatitude: -23.8883; decimalLongitude: -52.8086; **Identification:** identificationID: Rhagovelia robusta; identifiedBy: C. F. B. Floriano; **Event:** samplingProtocol: "D" Net; year: 2024; month: IV; **Record Level:** institutionCode: CEIOC; basisOfRecord: PreservedSpecimen
- **Type status:** Other material. **Occurrence:** recordedBy: C. F. B. Floriano; individualCount: 7; sex: male; lifeStage: adult; occurrenceID: 431985F7-0774-57F2-8946-D020E3A093DF; **Taxon:** genus: Rhagovelia; specificEpithet: robusta; taxonRank: species; scientificNameAuthorship: Gould, 1931; **Location:** stateProvince: Paraná; municipality: Tuneiras do Oeste; locality: Reserva Biológica das Perobas; decimalLatitude: -23.8797; decimalLongitude: -52.8194; **Identification:** identificationID: Rhagovelia robusta; identifiedBy: C. F. B. Floriano; **Event:** samplingProtocol: "D" Net; year: 2024; month: IV; **Record Level:** institutionCode: CEIOC; basisOfRecord: PreservedSpecimen
- **Type status:** Other material. **Occurrence:** recordedBy: C. F. B. Floriano; individualCount: 7; sex: female; lifeStage: adult; occurrenceID: 3D015527-E554-5019-B86E-41B37C873F26; **Taxon:** genus: Rhagovelia; specificEpithet: robusta; taxonRank: species; scientificNameAuthorship: Gould, 1931; **Location:** stateProvince: Paraná; municipality: Tuneiras do Oeste; locality: Reserva Biológica das Perobas; decimalLatitude: -23.8797; decimalLongitude: -52.8194; **Identification:** identificationID: Rhagovelia robusta; identifiedBy: C. F. B. Floriano; **Event:** samplingProtocol: "D" Net; year: 2024; month: IV; **Record Level:** institutionCode: CEIOC; basisOfRecord: PreservedSpecimen

##### Distribution

Brazil, Paraguay and Argentina.

##### Notes

First records from Paraná (Fig. 10F, Fig. 11C). Previously reported from Santa Catarina and Rio Grande do Sul (Polhemus 1997, Rodrigues et al. 2021).

#### Rhagovelia trepida

Bacon, 1948

CCC061A8-34E9-5C9A-AC43-DA68485AAAB4

##### Materials

- **Type status:** Other material. **Occurrence:** recordedBy: C. F. B. Floriano & T. Malacarne; individualCount: 2; sex: male; lifeStage: adult; occurrenceID: 221B4923-41B9-5240-A69A-18581E530492; **Taxon:** genus: Rhagovelia; specificEpithet: trepida; taxonRank: species; scientificNameAuthorship: Bacon, 1948; **Location:** stateProvince: Santa Catarina; municipality: Blumenau; locality: Parque Nacional da Serra do Itajaí, riacho pequeno ao lado da estrada; **Identification:** identificationID: Rhagovelia trepida; identifiedBy: C. F. B. Floriano; **Event:** samplingProtocol: "D" Net; year: 2017; month: IV; **Record Level:** institutionCode: CEIOC; basisOfRecord: PreservedSpecimen
- **Type status:** Other material. **Occurrence:** recordedBy: T. Roell & T. Polizei; individualCount: 4; sex: male; lifeStage: adult; occurrenceID: 8F22CFD3-02F2-5511-AC08-826575894EE8; **Taxon:** genus: Rhagovelia; specificEpithet: trepida; taxonRank: species; scientificNameAuthorship: Bacon, 1948; **Location:** stateProvince: Santa Catarina; municipality: Rio Negrinho; locality: Rio dos Bugres; decimalLatitude: -26.2772; decimalLongitude: -49.5344; **Identification:** identificationID: Rhagovelia trepida; identifiedBy: I. R. S. Cordeiro; **Event:** samplingProtocol: "D" Net; year: 2020; month: III; **Record Level:** institutionCode: CEIOC; basisOfRecord: PreservedSpecimen
- **Type status:** Other material. **Occurrence:** recordedBy: T. Roell & T. Polizei; individualCount: 9; sex: female; lifeStage: adult; occurrenceID: 2192AA3C-D33C-51A7-A4CF-92C14058CF21; **Taxon:** genus: Rhagovelia; specificEpithet: trepida; taxonRank: species; scientificNameAuthorship: Bacon, 1948; **Location:** stateProvince: Santa Catarina; municipality: Rio Negrinho; locality: Rio dos Bugres; decimalLatitude: -26.2772; decimalLongitude: -49.5344; **Identification:** identificationID: Rhagovelia trepida; identifiedBy: I. R. S. Cordeiro; **Event:** samplingProtocol: "D" Net; year: 2020; month: III; **Record Level:** institutionCode: CEIOC; basisOfRecord: PreservedSpecimen
- **Type status:** Other material. **Occurrence:** recordedBy: C. F. B. Floriano & T. Malacarne; individualCount: 7; sex: male; lifeStage: adult; occurrenceID: C5E7777E-B2E6-5CAE-A5AB-6FB9A5BD6177; **Taxon:** genus: Rhagovelia; specificEpithet: trepida; taxonRank: species; scientificNameAuthorship: Bacon, 1948; **Location:** stateProvince: Santa Catarina; municipality: Blumenau; locality: Parque Nacional da Serra do Itajaí, Parque Natural Municipal Nascentes do Garcia, riacho pequeno; decimalLatitude: -27.052; decimalLongitude: -49.088; **Identification:** identificationID: Rhagovelia trepida; identifiedBy: C. F. B. Floriano; **Event:** samplingProtocol: "D" Net; year: 2017; month: IV; **Record Level:** institutionCode: CEIOC; basisOfRecord: PreservedSpecimen
- **Type status:** Other material. **Occurrence:** recordedBy: C. F. B. Floriano & T. Malacarne; individualCount: 5; sex: female; lifeStage: adult; occurrenceID: 4F328629-6494-59D8-BDDB-59C50A23B6C6; **Taxon:** genus: Rhagovelia; specificEpithet: trepida; taxonRank: species; scientificNameAuthorship: Bacon, 1948; **Location:** stateProvince: Santa Catarina; municipality: Blumenau; locality: Parque Nacional da Serra do Itajaí, Parque Natural Municipal Nascentes do Garcia, riacho pequeno; decimalLatitude: -27.052; decimalLongitude: -49.088; **Identification:** identificationID: Rhagovelia trepida; identifiedBy: C. F. B. Floriano; **Event:** samplingProtocol: "D" Net; year: 2017; month: IV; **Record Level:** institutionCode: CEIOC; basisOfRecord: PreservedSpecimen

##### Distribution

Brazil.

##### Notes

Previously reported from Paraná, Santa Catarina and Rio Grande do Sul (Polhemus 1997, Magalhães et al. 2023).

#### Vellinae

Brullé, 1836

20A93EF7-70FE-595D-8E7E-46EB1954C2D1

#### Oiovelia

Drake & Maldonado-Capriles, 1952

8897B0C2-CAA7-5E25-92ED-C86466DA3EF7

#### Oiovelia brasiliensis

Moreira, Nessimian & Rúdio, 2010

F02CC8E5-6832-51BC-9879-D96F142F6A66

##### Materials

- **Type status:** Other material. **Occurrence:** recordedBy: C. F. B. Floriano & T. Polizei; individualCount: 1; sex: male; lifeStage: adult; occurrenceID: 6B3D53EF-85A4-54E4-9AB2-ACD11E9D2920; **Taxon:** genus: Oiovelia; specificEpithet: brasiliensis; taxonRank: species; scientificNameAuthorship: Moreira, Nessimian & Rúdio, 2010; **Location:** stateProvince: Santa Catarina; municipality: Águas Mornas; decimalLatitude: -27.694; decimalLongitude: -48.825; **Identification:** identificationID: Oiovelia brasiliensis; identifiedBy: C. F. B. Floriano; **Event:** samplingProtocol: "D" Net; year: 2016; month: IX; **Record Level:** institutionCode: CEIOC; basisOfRecord: PreservedSpecimen
- **Type status:** Other material. **Occurrence:** recordedBy: C. F. B. Floriano & T. Polizei; individualCount: 1; sex: male; lifeStage: adult; occurrenceID: C8C90455-7FA3-5C78-81A3-2F3F27D97AAD; **Taxon:** genus: Oiovelia; specificEpithet: brasiliensis; taxonRank: species; scientificNameAuthorship: Moreira, Nessimian & Rúdio, 2010; **Location:** stateProvince: Santa Catarina; municipality: São Bonifácio; locality: Riacho Dona Terezinha, Montanha; decimalLatitude: -27.9088; decimalLongitude: -48.9126; **Identification:** identificationID: Oiovelia brasiliensis; identifiedBy: C. F. B. Floriano; **Event:** samplingProtocol: "D" Net; year: 2016; month: IX; **Record Level:** institutionCode: CEIOC; basisOfRecord: PreservedSpecimen
- **Type status:** Other material. **Occurrence:** recordedBy: C. F. B. Floriano & T. Polizei; individualCount: 2; sex: male; lifeStage: adult; occurrenceID: 391CEE24-4065-5751-9613-0C5DBB25A24D; **Taxon:** genus: Oiovelia; specificEpithet: brasiliensis; taxonRank: species; scientificNameAuthorship: Moreira, Nessimian & Rúdio, 2010; **Location:** stateProvince: Rio Grande do Sul; municipality: Cambará do Sul; locality: Parque Nacional de Aparados da Serra, Córrego Preá; decimalLatitude: -29.17; decimalLongitude: -50.09; **Identification:** identificationID: Oiovelia brasiliensis; identifiedBy: C. F. B. Floriano; **Event:** samplingProtocol: "D" Net; year: 2016; month: IX; **Record Level:** institutionCode: CEIOC; basisOfRecord: PreservedSpecimen

##### Distribution

Brazil and Argentina.

##### Notes

**Figure 12.**
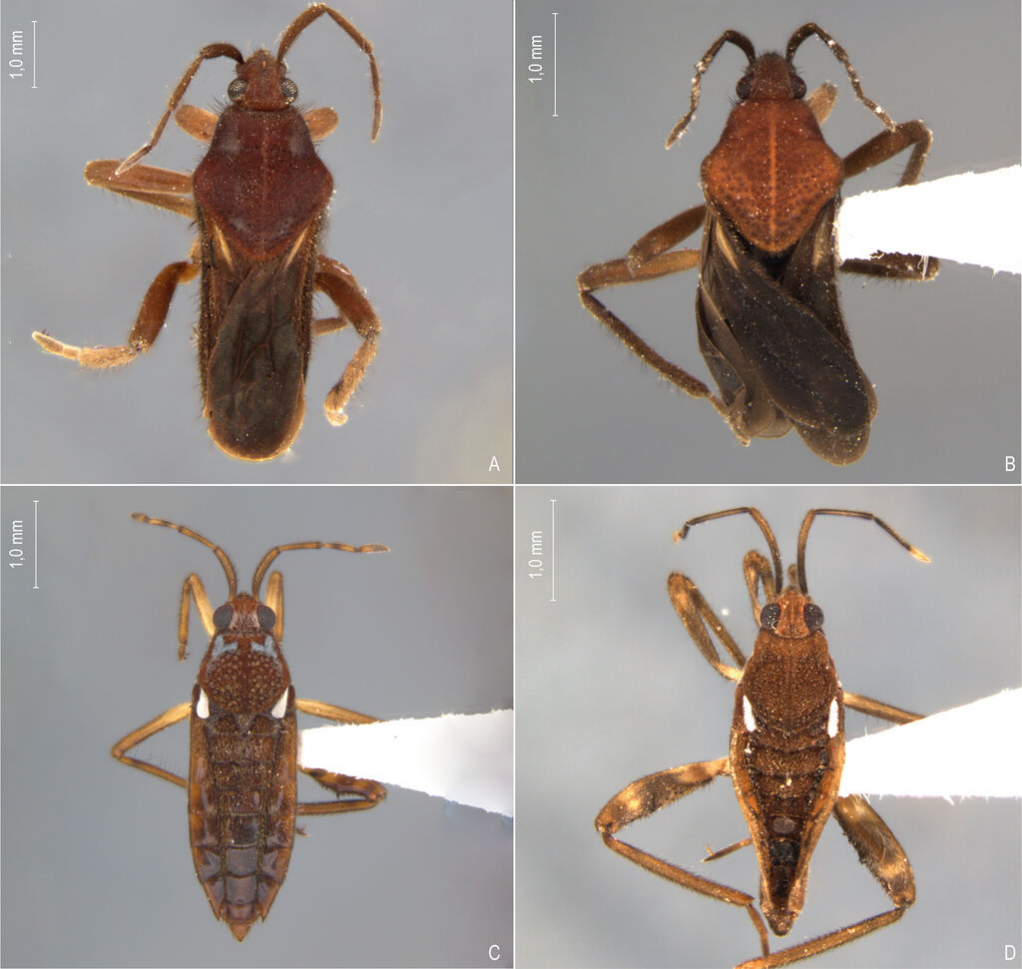
Veliidae from the South Region of Brazil, habitus, dorsal view. **A, B, D** Male; **C.** Female. **A** *Oiovelia braziliensis*; **B** *Oiovelia cunucunumana*; **C** *Stridulivelia astralis*; **D** *Stridulivelia ayacucho*, specimen damaged due to dehydration.

First records from Santa Catarina (Fig. 11B, Fig. 12A). Previously reported from Rio Grande do Sul (Moreira and Campos 2012, Rodrigues et al. 2021).

#### Oiovelia cunucunumana

Drake & Maldonado-Capriles, 1952

58F6484F-6345-54BA-A410-1B2FA75F38C2

##### Materials

- **Type status:** Other material. **Occurrence:** recordedBy: T. Roell & T. Polizei; individualCount: 1; sex: male; lifeStage: adult; occurrenceID: 45D878E9-1518-5E84-9A5F-AA67B996AC91; **Taxon:** genus: Oiovelia; specificEpithet: cunucunumana; taxonRank: species; scientificNameAuthorship: Drake & Maldonado-Capriles, 1952; **Location:** stateProvince: Santa Catarina; municipality: Mafra; locality: Rio Negro; decimalLatitude: -26.2117; decimalLongitude: -49.6286; **Identification:** identificationID: Oiovelia cunucunumana; identifiedBy: C. F. B. Floriano; **Event:** samplingProtocol: "D" Net; year: 2020; month: III; **Record Level:** institutionCode: CEIOC; basisOfRecord: PreservedSpecimen
- **Type status:** Other material. **Occurrence:** recordedBy: C. F. B. Floriano; individualCount: 10; sex: male; lifeStage: adult; occurrenceID: 57B69026-644D-5C8D-BCAC-E0E4EB238877; **Taxon:** genus: Oiovelia; specificEpithet: cunucunumana; taxonRank: species; scientificNameAuthorship: Drake & Maldonado-Capriles, 1952; **Location:** stateProvince: Paraná; municipality: Tuneiras do Oeste; locality: Reserva Biológica das Perobas, Rio Mouro; decimalLatitude: -23.8850; decimalLongitude: -52.8072; **Identification:** identificationID: Oiovelia cunucunumana; identifiedBy: C. F. B. Floriano; **Event:** samplingProtocol: "D" Net; year: 2024; month: IV; **Record Level:** institutionCode: CEIOC; basisOfRecord: PreservedSpecimen
- **Type status:** Other material. **Occurrence:** recordedBy: C. F. B. Floriano; individualCount: 5; sex: male; lifeStage: adult; occurrenceID: B5529779-1C0B-597A-802B-C099869A895B; **Taxon:** genus: Oiovelia; specificEpithet: conucunumana; taxonRank: species; scientificNameAuthorship: Drake & Maldonado-Capriles, 1952; **Location:** stateProvince: Paraná; municipality: Tuneiras do Oeste; locality: Reserva Biológica das Perobas, Rio Mouro; decimalLatitude: -23.8850; decimalLongitude: -52.8072; **Identification:** identificationID: Oiovelia conucunumana; identifiedBy: C. F. B. Floriano; **Event:** samplingProtocol: "D" Net; year: 2024; month: IV; **Record Level:** institutionCode: CEIOC; basisOfRecord: PreservedSpecimen

##### Distribution

Colombia, Venezuela, French Guiana, Brazil, Paraguay and Argentina.

##### Notes

First records from Paraná (Fig. 11B, Fig. 12B). Previously reported from Santa Catarina (Spangler 1986).

#### Platyvelia

Polhemus & Polhemus, 1993

1C9E486D-1B62-5403-8355-89809B90FCC9

#### Platyvelia brachialis

(Stål, 1860)

2FB76E0D-5DD1-5277-8E5C-8F6BE0D57828

##### Materials

- **Type status:** Other material. **Occurrence:** recordedBy: C. F. B. Floriano & T. Malacarne; individualCount: 3; sex: male; lifeStage: adult; occurrenceID: C55A1009-9FBF-5BCB-9CE4-63A54464B522; **Taxon:** genus: Platyvelia; specificEpithet: brachialis; taxonRank: species; scientificNameAuthorship: (Stål, 1860); **Location:** stateProvince: Santa Catarina; municipality: Blumenau; locality: Parque Nacional da Serra do Itajaí, Parque Natural Municipal Nascentes do Garcia, Lagoa Negra; decimalLatitude: -27.0532; decimalLongitude: -49.0878; **Identification:** identificationID: Platyvelia brachialis; identifiedBy: C. F. B. Floriano; **Event:** samplingProtocol: "D" Net; year: 2017; month: IV; **Record Level:** institutionCode: CEIOC; basisOfRecord: PreservedSpecimen
- **Type status:** Other material. **Occurrence:** recordedBy: C. F. B. Floriano & T. Malacarne; individualCount: 1; sex: female; lifeStage: adult; occurrenceID: 3ABAD684-778F-5209-ADB0-3E5BAEC99122; **Taxon:** genus: Platyvelia; specificEpithet: brachialis; taxonRank: species; scientificNameAuthorship: (Stål, 1860); **Location:** stateProvince: Santa Catarina; municipality: Blumenau; locality: Parque Nacional da Serra do Itajaí, Parque Natural Municipal Nascentes do Garcia, Lagoa Negra; decimalLatitude: -27.0532; decimalLongitude: -49.0878; **Identification:** identificationID: Platyvelia brachialis; identifiedBy: C. F. B. Floriano; **Event:** samplingProtocol: "D" Net; year: 2017; month: IV; **Record Level:** institutionCode: CEIOC; basisOfRecord: PreservedSpecimen

##### Distribution

United States, Mexico, Belize, Guatemala, Cuba, Jamaica, Dominican Republic, Haiti, El Salvador, Nicaragua, Grenada, Costa Rica, Panama, Trinidad & Tobago, Colombia, Venezuela, Suriname, Brazil, Peru and Argentina.

##### Notes

Previously reported from Santa Catarina (Polhemus and Polhemus 1993, Rodrigues et al. 2021).

#### Stridulivelia

Hungerford, 1929

7828568C-2196-58B9-9D5F-4D86280818AF

#### Stridulivelia astralis

(Drake & Harris, 1938)

DA930702-EA9A-521B-9C27-30A48A55F55B

##### Materials

- **Type status:** Other material. **Occurrence:** recordedBy: C. F. B. Floriano; individualCount: 2; sex: female; lifeStage: adult; occurrenceID: C386A4A0-A35E-5408-A722-930A1E41906A; **Taxon:** genus: Stridulivelia; specificEpithet: astralis; taxonRank: species; scientificNameAuthorship: (Drake & Harris, 1938); **Location:** stateProvince: Paraná; municipality: Tuneiras do Oeste; locality: Reserva Biológica das Perobas, Lagoa; decimalLatitude: -23.8894; decimalLongitude: -52.8144; **Identification:** identificationID: Stridulivelia astralis; identifiedBy: C. F. B. Floriano; **Event:** samplingProtocol: "D" Net; year: 2024; month: IV; **Record Level:** institutionCode: CEIOC; basisOfRecord: PreservedSpecimen

##### Distribution

Brazil, Paraguay and Argentina.

##### Notes

First record from Paraná (Fig. 11A, Fig. 12C). Previously reported from Rio Grande do Sul by Rodrigues et al. (2021).

#### Stridulivelia ayacucho

Polhemus & Spangler, 1995

08913D7B-05BC-563F-872E-BC79B3FF47D0

##### Materials

- **Type status:** Other material. **Occurrence:** recordedBy: C. F. B. Floriano; individualCount: 1; sex: male; lifeStage: adult; occurrenceID: 8711858B-24AF-5463-8C09-94FFA5BB170C; **Taxon:** genus: Stridulivelia; specificEpithet: ayacucho; taxonRank: species; scientificNameAuthorship: Polhemus and Spangler, 1995; **Location:** stateProvince: Paraná; municipality: Tuneiras do Oeste; locality: Reserva Biológica das Perobas; decimalLatitude: -23.8797; decimalLongitude: -52.8194; **Identification:** identificationID: Stridulivelia ayacucho; identifiedBy: C. F. B. Floriano; **Event:** samplingProtocol: "D" Net; year: 2024; month: IV; **Record Level:** institutionCode: CEIOC; basisOfRecord: PreservedSpecimen
- **Type status:** Other material. **Occurrence:** recordedBy: C. F. B. Floriano; individualCount: 2; sex: female; lifeStage: adult; occurrenceID: ED299F73-BA59-5F0B-84CA-A8D42C2B1F06; **Taxon:** genus: Stridulivelia; specificEpithet: ayacucho; taxonRank: species; scientificNameAuthorship: Polhemus and Spangler, 1995; **Location:** stateProvince: Paraná; municipality: Tuneiras do Oeste; locality: Reserva Biológica das Perobas; decimalLatitude: -23.8797; decimalLongitude: -52.8194; **Identification:** identificationID: Stridulivelia ayacucho; identifiedBy: C. F. B. Floriano; **Event:** samplingProtocol: "D" Net; year: 2024; month: IV; **Record Level:** institutionCode: CEIOC; basisOfRecord: PreservedSpecimen
- **Type status:** Other material. **Occurrence:** recordedBy: C. F. B. Floriano; individualCount: 1; sex: female; lifeStage: adult; occurrenceID: 929C4537-11F9-5900-839E-5BEDEA0DAEE3; **Taxon:** genus: Stridulivelia; specificEpithet: ayacucho; taxonRank: species; scientificNameAuthorship: Polhemus and Spangler, 1995; **Location:** stateProvince: Paraná; municipality: Tuneiras do Oeste; locality: Reserva Biológica das Perobas, Rio Saquarema; decimalLatitude: -23.8725; decimalLongitude: -52.7864; **Identification:** identificationID: Stridulivelia ayacucho; identifiedBy: C. F. B. Floriano; **Event:** samplingProtocol: "D" Net; year: 2024; month: IV; **Record Level:** institutionCode: CEIOC; basisOfRecord: PreservedSpecimen

##### Distribution

Venezuela, Suriname, Guyana, Brazil, Peru, Bolivia, Paraguay and Argentina.

##### Notes

First records from Paraná and the South Region of Brazil (Fig. 11D, Fig. 12A).

## Discussion

Before this study, 61 species of Gerromorpha had been recorded from the South Region of Brazil (Moreira 2025a, Moreira 2025b, Moreira 2025c, Moreira 2025d, Moreira 2025e). Our results include new distribution information for 45 species (Table 1), 36 of which are herein recorded for the first time from at least one of the three southern Brazilian States. Amongst them, *Microvelia lujanana* is recorded for the first time from Brazil, while 14 species are recorded for the first time from the South Region of the country (State abbreviations in parentheses): *Tachygerris adamsoni* (PR), *Microvelia hamadae* (PR), *Mi. hinei* (PR), *Mi. lujanana* (SC, RS), *Mi. picinguaba* (SC), *Mi. sousorum* (PR), *Rheumatobates minutus flavidus* (PR), *Hebrus parvulus* (SC), *Lipogomphus lacuniferus* (SC), *Hydrometra caraiba* (PR), *Rhagovelia brunae* (PR), *Rha. henryi* (SC), *Rha. ochroischion* (PR) and *S. ayacucho* (PR). Our data increase the number of semi-aquatic bugs known from the South Region from 60 to 75.

Our fieldwork included the following protected areas: Parque Nacional Aparados da Serra, Parque Nacional da Serra Geral, Parque Nacional da Serra do Itajaí, Parque Natural Municipal Nascentes do Garcia and Reserva Biológica das Perobas. Several species were collected exclusively within them, including *Hydrometra caraiba*, *H. fruhstorferi*, *Lipogomphus lacuniferus*, *Mesovelia amoena*, *Microvelia hamadae*, *Mi. hinei*, *Mi. picinguaba*, *Mi. sousorum*, *Rheumatobates minutus flavidus*, *Rhagovelia brunae*, *Rha. ochroischion*, *Rha. robusta*, *Platyvelia brachialis*, *Stridulivelia astralis*, *S. ayacucho* and *Tachygerris adamsoni*. This highlights the importance of these sites in maintaining populations of semi-aquatic bugs.

## Supplementary Material

XML Treatment for Gerridae

XML Treatment for Charmatometrinae

XML Treatment for Brachymetra

XML Treatment for Brachymetra albinervus

XML Treatment for Brachymetra albinervus albinervus

XML Treatment for Gerrinae

XML Treatment for Limnogonus

XML Treatment for Limnogonus aduncus

XML Treatment for Limnogonus aduncus aduncus

XML Treatment for Neogerris

XML Treatment for Neogerris lubricus

XML Treatment for Tachygerris

XML Treatment for Tachygerris adamsoni

XML Treatment for Microveliinae

XML Treatment for Microvelia

XML Treatment for Microvelia arca

XML Treatment for Microvelia braziliensis

XML Treatment for Microvelia hamadae

XML Treatment for Microvelia hambletoni

XML Treatment for Microvelia hinei

XML Treatment for Microvelia ioana

XML Treatment for Microvelia limaiana

XML Treatment for Microvelia longipes

XML Treatment for Microvelia lujanana

XML Treatment for Microvelia mimula

XML Treatment for Microvelia minima

XML Treatment for Microvelia novana

XML Treatment for Microvelia picinguaba

XML Treatment for Microvelia pulchella

XML Treatment for Microvelia sousorum

XML Treatment for Microvelia venustatis

XML Treatment for Rhagadotarsinae

XML Treatment for Rheumatobates

XML Treatment for Rheumatobates bonariensis

XML Treatment for Rheumatobates crassifemur

XML Treatment for Rheumatobates crassifemur crassifemur

XML Treatment for Rheumatobates minutus

XML Treatment for Rheumatobates minutus flavidus

XML Treatment for Trepobatinae

XML Treatment for Halobatopsis

XML Treatment for Halobatopsis platensis

XML Treatment for Halobatopsis spiniventris

XML Treatment for Metrobates

XML Treatment for Metrobates plaumanni plaumanni

XML Treatment for Hebridae

XML Treatment for Hebrinae

XML Treatment for Hebrus

XML Treatment for Hebrus parvulus

XML Treatment for Lipogomphus

XML Treatment for Lipogomphus lacuniferus

XML Treatment for Hydrometridae

XML Treatment for Hydrometrinae

XML Treatment for Hydrometra

XML Treatment for Hydrometra argentina

XML Treatment for Hydrometra caraiba

XML Treatment for Hydrometra fruhstorferi

XML Treatment for Mesoveliidae

XML Treatment for Mesoveliinae

XML Treatment for Mesovelia

XML Treatment for Mesovelia amoena

XML Treatment for Mesovelia bila

XML Treatment for Veliidae

XML Treatment for Rhagoveliinae

XML Treatment for Rhagovelia

XML Treatment for Rhagovelia brunae

XML Treatment for Rhagovelia henryi

XML Treatment for Rhagovelia lucida

XML Treatment for Rhagovelia occulcata

XML Treatment for Rhagovelia ochroischion

XML Treatment for Rhagovelia robusta

XML Treatment for Rhagovelia trepida

XML Treatment for Vellinae

XML Treatment for Oiovelia

XML Treatment for Oiovelia brasiliensis

XML Treatment for Oiovelia cunucunumana

XML Treatment for Platyvelia

XML Treatment for Platyvelia brachialis

XML Treatment for Stridulivelia

XML Treatment for Stridulivelia astralis

XML Treatment for Stridulivelia ayacucho

## Figures and Tables

**Table 1.** Checklist of Gerromorpha from the South Region of Brazil. PR: Paraná; SC: Santa Catarina; RS: Rio Grande do Sul; N: New record; P: Previously recorded; - No record.

**Taxa**	**PR**	**SC**	**RS**	**References**
** Gerridae **				
** Charmatometrinae **				
***Brachymetra* Mayr, 1865**				
*B. albinervus albinervus* (Amyot & Serville, 1843)	N	N	P	Rodrigues et al. (2021)
*B. furva* Drake, 1957	P	-	-	Rodrigues et al. (2012)
** Gerrinae **				
***Limnogonus* Stål, 1868**				
*L. aduncus aduncus* Drake & Harris, 1933	P	P	-	Moreira et al. (2011b), Moreira and Campos (2012), Rodrigues et al. (2012)
*L. ignotus* Drake & Harris, 1934	-	P	P	Moreira et al. (2011b)
*L. profugus* Drake & Harris, 1930	-	-	P	Rodrigues et al. (2021)
***Neogerris* Matsumura, 1913**				
*N. lubricus* (White, 1879)	-	N	P	Rodrigues et al. (2021)
***Tachygerris* Drake, 1957**				
*T. adamsoni* (Drake, 1942)	N	-	-	
** Halobatinae **				
***Halobates* Eschscholtz, 1822**				
*H. micans* Eschscholtz, 1822	P	P	-	Andres et al. (2003), Dias and Lopes (2009)
** Microveliinae **				
***Husseyella* Herring, 1955**				
*H. halophila* Drake, 1958	-	P	-	Drake (1958a), Floriano et al. (2024)
***Microvelia* Westwood, 1834**				
*M. arca* Drake, 1958	-	P	-	Drake (1958a)
*M. braziliensis* McKinstry, 1937	N	P	P	Drake and Plaumann (1955), Rodrigues et al. (2021)
*M. hamadae* dos Santos, Rodrigues, Couceiro & Moreira, 2021	N	-	-	
*M. hambletoni* Drake, 1951	N	P	N	Drake (1951)
*M. hinei* Drake, 1920	N	-	-	
*M. inannana* Drake & Hottes 1952	-	P	-	Drake and Plaumann (1955)
*M. ioana* Drake & Hottes, 1952	-	P	N	Magalhães et al. (2021)
*M. limaiana* Drake, 1951	N	P	-	Drake (1951)
*M. longipes* Uhler, 1894	N	P	-	Drake and Plaumann (1955)
*M. lujanana* Drake, 1951	-	N	N	Drake (1951)
*M. mimula* White, 1879	N	P	P	Drake and Hussey (1951), Rodrigues et al. (2021)
*M. minima* Drake, 1952	N	P	N	Drake (1952a)
*M. novana* Drake & Plaumann, 1955	N	P	-	Drake and Plaumann (1955)
*M. picinguaba* Moreira & Barbosa, 2011	-	N	-	
*M. pulchella* Westwood, 1834	N	P	P	Drake and Plaumann (1955), Rodrigues et al. (2021)
*M. sousorum* dos Santos, Rodrigues, Couceiro & Moreira, 2021	N	-	-	
*M. stellata* Kirkaldy, 1902	-	P	-	Drake and Plaumann (1955)
*M. venustatis* Drake & Harris, 1933	N	P	-	Drake and Plaumann (1955)
***Xiphovelopsis* Floriano & Moreira, 2024**				
*X. lacunana* (Drake & Plaumann, 1953)	-	P	-	Polhemus (1977), Floriano et al. (2024)
** Rhagadotarsinae **				
***Rheumatobates* Bergroth, 1892**				
*R. bonariensis* (Berg, 1898)	N	P	P	Hungerford (1954), Neri et al. (2005), Secretaria do Meio Ambiente e Infraestrutura do Estado do Rio Grande do Sul (2014), Rodrigues et al. (2021)
*R. crassifemur crassifemur* Esaki, 1926	N	-	P	Rodrigues et al. (2021)
*R. minimus* Drake, 1958	-	P	-	Polhemus and Spangler (1989)
*R. minutus flavidus* Drake & Harris, 1942	N	-	-	
** Trepobatinae **				
***Halobatopsis* Bianchi, 1896**				
*H. platensis* (Berg, 1879)	P	-	P	Nieser (1970), Moreira and Campos (2012), Secretaria do Meio Ambiente e Infraestrutura do Estado do Rio Grande do Sul (2014), Rodrigues et al. (2021)
*H. spiniventris* Drake & Harris, 1936	P	P	P	Nieser and Melo (1999), Moreira and Campos (2012), Cordeiro and Moreira (2015), Rodrigues et al. (2021)
***Metrobates* Uhler, 1871**				
*M. plaumanni plaumanni* Hungerford, 1951	P	P	-	Hungerford (1951), Armisén et al. (2022)
*M. vigilis* Drake, 1958	-	P	-	Drake (1958b)
***Ovatametra* Kenaga, 1942**				
*O. gualeguay* Bachmann, 1966	-	-	P	Rodrigues et al. (2021)
*O. thaumana* Drake, 1959	P	-	-	Drake (1959)
** Hebridae **				
** Hebrinae **				
***Hebrus* Curtis, 1833**				
*H. limnaeus* Drake & Chapman, 1958	-	P	-	Drake and Chapman (1958)
*H. parvulus* Stål, 1860	-	N	-	
*H. plaumanni* Porter, 1952	-	P	-	Porter (1952)
***Lipogomphus* Berg, 1879**				
*L. lacuniferus* Berg, 1879	-	N	-	
** Hydrometridae **				
** Hydrometrinae **				
***Hydrometra* Latreille, 1797**				
*H. argentina* Berg, 1879	P	P	P	Neri et al. (2005), University of California, Berkeley (2009), Secretaria do Meio Ambiente e Infraestrutura do Estado do Rio Grande do Sul (2014), Rodrigues et al. (2021)
*H. caraiba* Guérin-Méneville, 1856	N	-	-	
*H. fruhstorferi* Hungerford & Evans, 1934	P	P	N	Drake (1952b), Cordeiro and Moreira (2015)
*H. sztolcmani* Jaczewski, 1928	P	-	P	Jaczewski (1928a), Secretaria do Meio Ambiente e Infraestrutura do Estado do Rio Grande do Sul (2014)
** Mesoveliidae **				
** Mesoveliinae **				
***Mesovelia* Mulsant & Rey, 1852**				
*M. amoena* Uhler, 1894	P	N	-	Jaczewski (1928b)
*M. bila* Jaczewski, 1928	P	P	N	Jaczewski (1928b), Floriano et al. (2017)
*M. mulsanti* White, 1879	P	P	P	Jaczewski (1928b), Neering (1954), Rodrigues et al. (2021)
** Veliidae **				
** Rhagoveliinae **				
***Rhagovelia* Mayr, 1865**				
*R. brunae* Magalhães & Moreira, 2016	N	-	-	
*R. henryi* Polhemus, 1997	-	N	-	
*R. janeira* Drake, 1953	P	P	P	Bacon (1956), Nieser and Melo (1997), Polhemus (1997), Nieser and Polhemus (1999), Cordeiro and Moreira (2015)
*R. lucida* Gould, 1931	P	P	P	Bacon (1956), Polhemus (1997), Moreira and Campos (2012), Cordeiro and Moreira (2015)
*R. novana* Drake & Plaumann, 1955	-	-	P	Museo Civico di Rovereto (2010)
*R. occulcata* Drake, 1959	P	N	-	Drake (1959)
*R. ochroischion* Nieser & Melo, 1997	N	-	-	
*R. paulana* Drake, 1953	-	P	-	Bacon (1956)
*R. plaumanni* Polhemus, 1997	P	P	P	Polhemus (1997)
*R. rivulosa* Polhemus & Polhemus, 1985	-	P	P	Polhemus and Polhemus (1985), Nieser and Melo (1999), Rodrigues et al. (2021)
*R. robusta* Gould, 1931	N	P	P	Polhemus (1997), Rodrigues et al. (2021)
*R. sabrina* Drake, 1958	P	P	-	Nieser and Polhemus (1999)
*R. sooretama* Moreira, Nessimian & Rúdio, 2010	P	-	-	Moreira and Campos (2012)
*R. thaumana* Drake, 1958	-	P	P	Drake (1958a), Moreira and Campos (2012)
*R. trepida* Bacon, 1948	P	P	P	Polhemus (1997), Magalhães et al. (2023)
*R. zela* Drake, 1959	-	P	-	Drake (1959)
** Veliinae **				
***Callivelia* Polhemus 2021**				
*C. bipunctata* (Rodrigues, Moreira, Nieser, Chen & Melo, 2014)	-	-	P	Rodrigues et al. (2021)
***Oiovelia* Drake & Maldonado-Capriles, 1952**				
*O. brasiliensis* Moreira, Nessimian & Rúdio, 2010 (Fig. 12)	-	N	P	Moreira and Campos (2012), Rodrigues et al. (2021)
*O. cunucunumana* Drake & Maldonado-Capriles, 1952 (Fig. 12)	N	P	-	Spangler (1986)
***Paravelia* Breddin, 1898**				
*P. bachmanni* Rodrigues & Moreira, 2016	-	P	-	Rodrigues and Moreira (2016)
*P. bromelicola* Rodrigues & Moreira, 2016	-	P	-	Rodrigues and Moreira (2016)
*P. capixaba* Moreira, Nessimian and Rúdio, 2010	-	-	P	Rodrigues et al. (2021)
***Platyvelia* Polhemus & Polhemus, 1993**				
*P. brachialis* (Stål, 1860)	-	P	-	Polhemus and Polhemus (1993), Rodrigues et al. (2021)
***Steinovelia* Polhemus & Polhemus, 1993**				
*S. virgata* (White, 1879)	-	P	P	Polhemus and Polhemus (1993), Moreira et al. (2020), Heiss et al. (2022)
***Stridulivelia* Hungerford, 1929**				
*S. astralis* (Drake & Harris, 1938) (Fig. 12)	N	-	P	Rodrigues et al. (2021)
*S. ayacucho* Polhemus & Spangler, 1995 (Fig. 12)	N	-	-	
